# The decision uncertainty toolkit: Risk measures and visual outputs to support decision making during public health crises

**DOI:** 10.1371/journal.pone.0332522

**Published:** 2025-10-01

**Authors:** Megan Wiggins, Marie Varughese, Ellen Rafferty, Sasha van Katwyk, Christopher McCabe, Jeff Round, Erin Kirwin

**Affiliations:** 1 Institute of Health Economics, Edmonton, Alberta, Canada; 2 Department of Mathematical and Statistical Sciences, University of Alberta, Edmonton, Alberta, Canada; 3 Faculty of Medicine and Dentistry, University of Alberta, Edmonton, Canada; 4 School of Public Health, University of Alberta, Edmonton, Canada; 5 Centre for Public Health and Queens Management School, Queens University Belfast, Belfast, Northern Ireland, United Kingdom; 6 Health Organisation, Policy, and Economics (HOPE), School of Health Sciences, University of Manchester, Manchester, United Kingdom; IST: Universidade de Lisboa Instituto Superior Tecnico, PORTUGAL

## Abstract

**Background:**

During public health crises such as the COVID-19 pandemic, decision-makers relied on infectious disease models to evaluate policy options. Often, there is a high degree of uncertainty in the evidence base underpinning these models. When there is increased uncertainty, the risk of selecting a policy option that does not align with the intended policy objective also increases; we term this decision risk. Even when models adequately capture uncertainty, the tools used to communicate their outcomes, underlying uncertainty, and associated decision risk have often been insufficient. Our aim is to support infectious disease modellers and decision-makers in interpreting and communicating decision risk when evaluating multiple policy options.

**Methods:**

We developed the Decision Uncertainty Toolkit by adapting methods from health economics and infectious disease modelling to improve the interpretation and communication of uncertainty. Specifically, we developed a quantitative measure of decision risk as well as a suite of risk visualizations. We refined the toolkit contents based on feedback from early dissemination through conferences and workshops.

**Results:**

The Decision Uncertainty Toolkit: (i) adapts and extends existing health economics methods for characterization, estimation, and communication of uncertainty to infectious disease modelling, (ii) introduces a novel risk measure that quantitatively captures the downside risk of policy alternatives, (iii) provides visual outputs for dissemination and communication of uncertainty and decision risk, and (iv) includes instructions on how to use the toolkit, standard text descriptions and examples for each component. The use of the toolkit is demonstrated through a hypothetical example.

**Conclusion:**

The Decision Uncertainty Toolkit improves existing methods for communicating infectious disease model results by providing additional information regarding uncertainty and decision risk associated with policy alternatives. This empowers decision-makers to consider and evaluate decision risk more effectively when making policy decisions. Improved understanding of decision risk can improve outcomes in future public health crises.

## Introduction

During public health crises such as the COVID-19 pandemic, decision-makers relied on infectious disease models to predict and estimate the impact of policy alternatives on policy objectives, including health outcomes and health system activity levels. Inevitably there is uncertainty in the evidence base informing any infectious disease model, and this uncertainty can be reported in the model outputs and the predictions based upon them [1–6]. How uncertainty in infectious disease model results is communicated to non-modellers was highlighted as a key area requiring improvement in a study that explored the complex relationships between models, decision-making, the media and the public during the COVID-19 pandemic in the United Kingdom [7]. The study noted the nature of the pandemic required rapid decision-making, however communicating uncertainty in model results can be time consuming and, as a result, is not always considered in the decision-making process. How to most effectively communicate the nuances of model uncertainty to non-modellers is an active area of research [8,9].

One way to make infectious disease model results more understandable to a broad audience is to present them using summary statistics, such as the mean or median, irrespective of how uncertainty is handled in the model. The Imperial College COVID-19 Response Team during the COVID-19 pandemic used this approach, as did many others [3,10,11]. While these summary statistics can help simplify the communication of results, they can also mask important aspects of uncertainty. When decision-makers (including those responsible for briefing them) are presented with mean or median outputs only, understandably, they often assume symmetry in the distribution of both the potential benefits and downside risks. The inclusion of percentile ranges along with the mean or median outcomes is another common approach and can help to illustrate uncertainty visually [12–14]. However, policy outcomes (such as hospitalizations or number of cases) usually do not follow symmetric probability distributions, and even disaggregated percentile-based reporting can mask important asymmetries in the distribution of uncertainty. For example, the non-linear dynamic of community transmission means that even a small degree of uncertainty in the predicted effectiveness of a policy option can lead to large differences in the uncertainty in predicted outcomes.

A direct implication of uncertainty in model outputs is that there is a risk of making the wrong decision – selecting a policy option where the observed outcome does not best align with the intended policy objective. For example, if the policy objective is that hospitalizations remain below the available hospital capacity, there is a risk that the implemented policy fails to keep hospitalisations below this threshold. We define this as decision risk: the risk that the outcome of the chosen policy diverges from the policy objective. Decision risk can increase as uncertainty grows, and can differ greatly between policy alternatives. When uncertainty is poorly measured or communicated, managing decision risk becomes even more challenging for decision-makers.

Economic and infectious disease models share many common features, including: (i) comparing the impact of different policy options on outcomes important to decision-makers and (ii) reflecting uncertainty in model parameter estimates through multiple model runs using different parameter sets (e.g., probabilistic sensitivity, uncertainty, or Bayesian inference analysis) [15,16]. Building on these methods, health economists have developed tools and visualization techniques to represent uncertainty in model outputs and convey the associated decision risk to decision-makers [15,17–19]. These visuals aid decision-making by presenting model outputs in relation to the stated policy objective, often presented as a predefined threshold [19–21].

We report the development of a suite of tools to support both infectious disease modellers and decision-makers in effectively communicating and interpreting decision risk when evaluating multiple policy alternatives. The Decision Uncertainty Toolkit (DUT): (i) adapts and extends existing health economics methods for the characterization, estimation, and communication of uncertainty and decision risk to infectious disease modelling, (ii) introduces a novel risk measure that quantitatively captures decision risk in infections disease model outputs, (iii) provides visual outputs for dissemination and communication of uncertainty and decision risk, and (iv) includes instructions on how to use the toolkit, as well as standard text descriptions and examples for each toolkit component.

## Methods

### Premise

The DUT was developed based on two key premises:

**Models can be used to produce quantitative estimates of decision uncertainty.** During a public health crisis, decision-makers must make decisions with the best available evidence, which is inevitably incomplete. During the COVID-19 pandemic, decision-makers relied on infectious disease models to predict and estimate the impact of policy alternatives on health and system outcomes [22–24]. Models built based on incomplete and/or dynamically changing evidence produce uncertain results. Infectious disease modellers can quantitatively capture some of this parameter uncertainty using current methods such as probabilistic sensitivity, uncertainty, or Bayesian inference analysis. However, effectively communicating these results to decision-makers has been emphasised as a key area requiring improvement [7]. Although using summary statistics and percentile ranges to communicate model results is common, as it can help simplify the communication, this approach can also mask important aspects of uncertainty and decision risk associated with different policy alternatives [3,10–14].

**Decisions can be defined in relation to stated policy objectives.** For example, a policy objective may be to ensure that the number of individuals requiring hospitalization remains below the available hospital capacity. The stated policy objective establishes the criteria for differentiating between policy successes and failures. In this case, exceeding hospital capacity is more undesirable than not exceeding it. Therefore, the stated policy objective acts as a threshold against which risks can be evaluated. Risk can be measured in terms of the probability of an undesirable outcome occurring (i.e., exceeding the policy objective), the magnitude of deviations from the stated policy objective, and the duration of these deviations.

Building upon these premises, we developed the DUT with the intention for it to be used to evaluate the impact of policy alternatives on outcomes compared to a pre-defined baseline scenario. The baseline scenario is typically defined as maintaining the status quo or a scenario where no mitigation policies are implemented (i.e., a ‘do nothing’ or ‘existing policy’ scenario). The toolkit aims to visually present the decision risk associated with various policy alternatives, and to estimate the magnitude of harm associated with the decision risk for each decision alternative.

### Engagement with modellers and decision-makers

We engaged with modellers, policy makers and decision-makers to develop the DUT, through early dissemination of the work in progress at conferences, meetings, and organized workshops. Two workshops were conducted in February and March 2024, targeting infectious disease modellers (n = 9) and decision-makers (n = 7) separately. Decision maker participants held executive, analyst, and policy roles within provincial and federal government agencies. The infectious disease modeller workshop included modellers who had used the toolkit components prior to the session and provided feedback on intuitiveness, interpretability, and usefulness. Details about the sessions, including distributed materials, are available via the Supplementary Material. Participants in the infectious disease modellers workshop were given access to the code for the toolkit beforehand, allowing them to test the code with their own model outputs. Feedback on the functionality and usability of the code was collected during the modellers’ workshop. These engagements aimed to obtain feedback from multiple perspectives on each toolkit component.

## Results

### Risk measure

We developed a novel measure of risk that can be applied to infectious disease model outputs. The risk measure quantifies the downside risk of a given policy option by capturing the probability of not achieving the stated policy objective, the magnitude of deviations from the stated policy objective, and the duration of deviations from the policy objective.

The expected risk of each policy alternative can be calculated using the outputs from multiple model runs with different input parameter sets, (See Box 1). The decision threshold is defined as *D*_*t*_, which is determined by the policy objective, and can be a maximum or a minimum. The total number of model runs is defined as *N*, while *t* *=* *t*_*min*_*… t*_*max*_ defines the simulation time over which the expected risk is calculated, and *O*_*nt*_ defines the model outcome result for simulation run *n* at time *t*.

**Box 1. Risk measure formula**

$Expected\;Risk=\left\{ \begin{array}{c} \frac{\left[\sum_{n=\text{1}}^{N}\sum_{t=t_{min}}^{t_{max}}(\text{max}\left(D_{t},O_{nt}\right)-D_{t})\right]}{N}\;if\;D_{t}\;is\;a\;maximum\\ \frac{\left[\sum_{n=\text{1}}^{N}\sum_{t=t_{min}}^{t_{max}}{(D}_{t}-\text{min}\left(D_{t},O_{nt}\right))\right]}{N}\;if\;D_{t}\;is\;a\;minimum \end{array}\right.\;$

*Where n = 1…N are the number of model runs in the analysis, t = t*_*min*_*… t*_*max*_ *is the simulation time,* $O_{nt}$ *is the model outcome result for simulation run n at time t, and D*_*t*_ *is the decision threshold at a given time (the stated policy objective).*

*The Expected Risk value calculation implies the following assumptions: (i)* ***distribution neutrality of deviations from D***_***t***_*: a certain risk of exceeding D*_*t*_ *by 10 has the same value as a risk distributed equally of an exceedance of either 0 or 20; and (ii)* ***linearity in cumulative risk****: an exceedance of 1 over 100 days has the same value as an exceedance of 10 over 10 days.*

The expected risk is calculated over a pre-defined time range (*t* *=* *t*_*min*_*…t*_*max*_), which should be selected to capture important features of the decision problem, such as the maximum duration for which any of the policy options being considered might be adopted. The decision threshold (*D*_*t*_) can be either a maximum, such as a maximum hospital capacity, or a minimum, such as a target number of vaccinations to be delivered. The decision threshold (*D*_*t*_) can also have different values for different time periods if, for example, the hospital capacity is expected to change over time. To ensure a consistent basis for comparison, it is important that all decision threshold (*D*_*t*_) values, the total number of simulations *(N)*, and the time range (*t* *=* *t*_*min*_*…t*_*max*_) are consistent across the baseline scenario and each of the policy alternatives being compared.

The expected risk calculation evaluates the model outcomes for each simulation run relative to the decision threshold (i.e., the policy objective) over the specified time range. If the threshold *D*_*t*_ is a maximum, for each simulation run (*n*), the magnitude of any deviation (*O*_*nt*_) exceeding the threshold (*D*_*t*_) is summed at each time point within the range (*t*). If the threshold *D*_*t*_ is a minimum, it instead sums the amount by which each run falls below the threshold at each time point within the range. These summed deviations are then averaged across all simulation runs (*N*). This average value accounts for both the magnitude of deviations (i.e., how large the deviations are) and also the probability (i.e., how often deviations occur), providing an interpretable estimate of the risk of failing to meet the policy objective.

The expected risk values for each policy alternative are challenging to interpret independently. A more intuitive understanding can be achieved by using a pre-defined baseline scenario as a comparator and calculating the percent change in risk relative to the baseline scenario. We defined this as the policy risk impact, which can be calculated for each policy alternative as described in Box 2.

**Box 2. Policy risk impact formula**

${Policy\;risk\;impact}_{Policy\;i}=\frac{{Expected\;risk}_{Policy\;i}-{Expected\;risk}_{Baseline}}{{Expected\;risk}_{Baseline}}$

*Where i is the policy option indicator (e.g., Policy A or Policy 1); Expected* *risk*_*Policy i*_ *and Expected risk*_*Baseline*_ *are calculated used the formula in* *Box 1*.

## Visuals

We developed a series of visual outputs that build upon those used in health economics. These visuals are designed to facilitate the intuitive and direct interpretation of model outputs and associated decision risk by infectious disease modellers and decision-makers (see Box 3). The visual and numerical presentation of decision risk are designed as complementary outputs to support decision-makers’ deliberations.

**Box 3. Visual outputs**

**Table:** 

**Time-outcome fan plot with embedded decision threshold:** The trajectory of the outcome is summarized over time and plotted using the mean value for a given policy alternative. Uncertainty is characterized by shading the 50% and 95% credible intervals (calculated as 25^th^ and 75^th^ percentiles and 2.5^th^ and 97.5^th^ percentiles, respectively). The decision threshold is shown directly on the plot to provide a clear reference point for interpreting the outcome values.	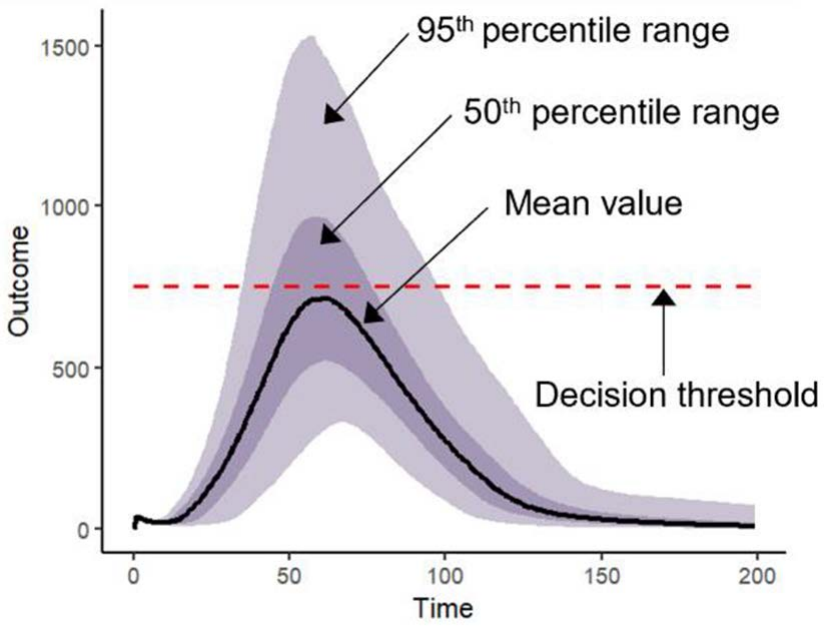
**Probability density plots with risk shading:** The probability density of the highest (or lowest if the threshold is a minimum) projected outcome across simulation runs is plotted for a given policy alternative. The decision threshold is shown directly on the plot as a vertical line. The area under the probability density curve where the threshold value is exceeded is shaded to visually display the downside risk of the policy alternative.	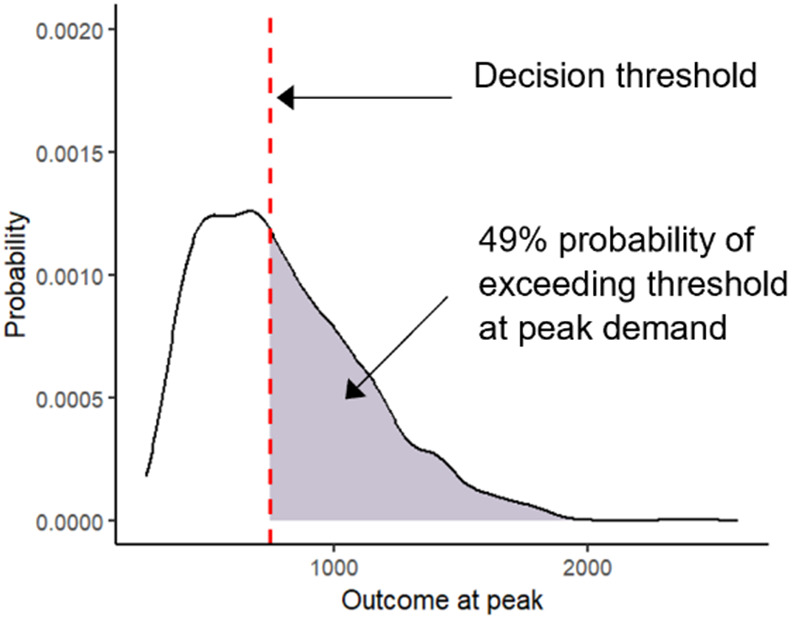
**Raincloud plots:** The probability densities of the highest (or lowest if the threshold is a minimum) projected outcome across simulation runs are plotted for each policy alternative alongside corresponding box plots, which indicate the mean and 50^th^ percentile range. These plots are presented collectively on a single graph to facilitate visual comparison of the policy alternatives. The decision threshold is shown directly on the plot as a vertical line to provide a clear reference point for interpreting the outputs.	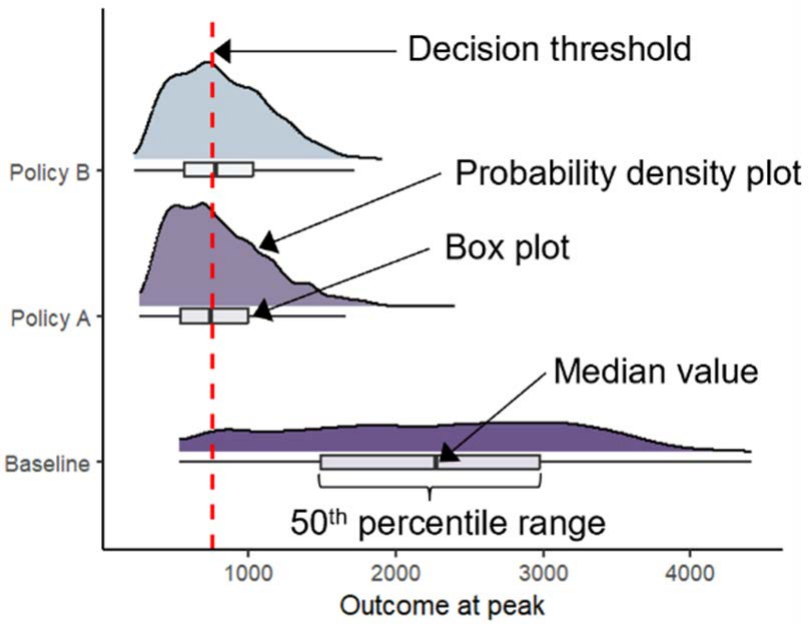
**Temporal probability density plots:** The probability density of the highest (or lowest if the threshold is a minimum) projected outcome across simulation runs is plotted in the center of the graph for a given policy alternative. Above and below, the probability density of the outcome at specified time points relative to the time of the highest (or lowest) project outcome is plotted to visually illustrate how uncertainty, and therefore risk, changes over time. The decision threshold is shown directly on the plot as a vertical line to provide a clear reference point for interpreting the outputs.	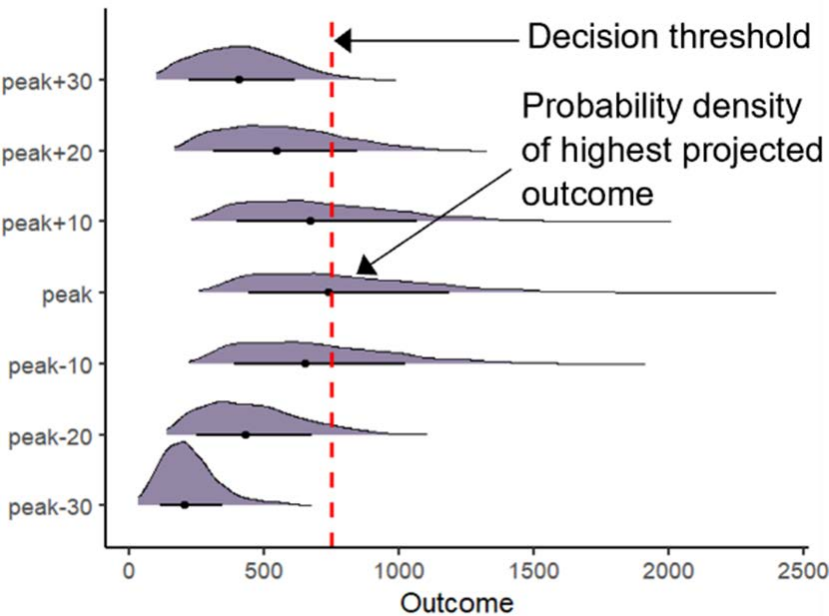

### Example scenario

To demonstrate the application of the DUT in the workshops, we considered a hypothetical scenario involving a decision-maker tasked with choosing between policy alternatives for managing COVID-19 in 2020. Each policy option was expected to impact the number of individuals requiring hospitalization; therefore, the outcome of interest was hospital demand, defined as the expected hospital census (i.e., the current number of people hospitalized). The policy objective was to ensure that hospital demand remained below the available daily hospital capacity of 750 patients, which defined the decision threshold. We considered a baseline scenario that represented the expected outcome in the absence of any intervention (i.e., taking no action) and two hypothetical policy alternatives: (i) Policy A – close schools and (ii) Policy B – mandatory masking. We considered a time range of 200 days starting from time zero, corresponding to the first day of policy implementation. We used the model outputs for 1,727 simulation runs that incorporated parameter uncertainty for the baseline scenario and each policy option. Note that these model outputs were modified from existing work solely to demonstrate the use case for the DUT. They do not represent actual data or outcomes, and the number of simulation runs is simply a byproduct of modifying the original work.

### Presenting results using common methods from published literature

**Fig 1:**
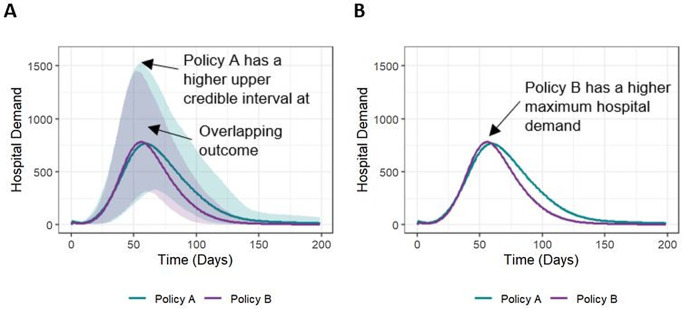
Common presentation of model outcomes over time. This figure illustrates a common presentation of model outputs. The mean (solid line) with 95% credible intervals (shading) of 1,727 simulations of hospital demand is displayed for two policy alternatives: Policy A – close schools and Policy B – mandatory masking. (A) shows the overlapping distributions of hospital demand, while (B) shows the median hospital demand for each policy.

Fig 1 shows a graphical presentation of model outputs for Policy A and Policy B, using common methods from published literature [3,10–14]. In Fig 1A, the overlapping distributions of hospital demand for Policy A and Policy B indicate uncertainty in the probability of either policy being preferred, but the relative probability of different outcomes in each shaded area is unclear. When decision-makers are presented with mean (or median) outputs, as in Fig 1B, this uncertainty and the associated risk are not communicated. While this may simplify communication of the model results, failure to communicate uncertainty can mask the decision risk associated with each policy, influencing how decision-makers understand, advocate for, and ultimately choose between policy options.

Consider the mean values plotted in Fig 1B and presented in Table 1. In Table 1, the magnitude of capacity exceedance is calculated by taking the greater of zero (no exceedance) and the difference between the maximum or peak hospital demand and the daily hospital capacity of 750 patients. The probability of exceeding capacity is calculated as the number of simulation runs in the probabilistic sensitivity analysis where the threshold is exceeded for one or more days, divided by the total number of simulation runs (1,727). The number of demand days is calculated by summing the daily hospital demand across the model time frame for each of the 1,727 simulation runs.

**Table 1:** Common presentation of model output measures.

Measure	Policy A	Policy B
**Maximum hospital demand, Mean (95% CI)**	767 (333 − 1,533)	781 (313 − 1,452)
**Magnitude of capacity exceedance, Mean (95% CI)**	17 (0 - 783)	31 (0 - 702)
**Probability of exceeding capacity (%)**	49%	53%
**Number of demand days, Mean (95% CI)**	48,393 (15,194 − 106,582)	39,809 (12,187 − 82,522)

**Caption:** 95% CI refers to the 95% credible intervals calculated as the 2.5^th^ and 97.5^th^ percentiles of the output distribution.

Policy A results in a lower maximum hospital demand, and the expected magnitude of capacity exceedance at the maximum hospital demand is also lower, which would be preferred. Hospital capacity was exceeded in 49% of the simulation runs with Policy A and 53% of the simulation runs with Policy B. Therefore, hospital capacity is more likely to be exceeded under Policy B. However, the hospital demand under Policy B peaks and falls more rapidly than under Policy A, resulting in fewer total days of demand (‘demand days’, the sum of the daily hospital demand across the entire model time frame).

These common measures highlight the potential for decision uncertainty, with different policy options appearing preferable based on different output measures. Furthermore, by ignoring the distribution of uncertainty, decision-makers cannot readily appreciate the trade-off between the expected hospital demand (the mean value) and the risk of exceeding hospital capacity. For example, the upper 95% credible interval for maximum hospital demand in Table 1 shows that Policy A and Policy B have a 2.5% probability of exceeding hospital capacity by 783 and 702 beds or more, respectively. Even though the mean results indicate that Policy A would result in a lower maximum hospital demand, the consequences of exceeding hospital capacity by a larger amount due to uncertainty in Policy A may be more severe. This highlights the need to capture not only the probability of failing to meet a policy target but also the magnitude of the excess if the policy target is missed.

### Results presentation using the decision uncertainty toolkit

**Fig 2:**
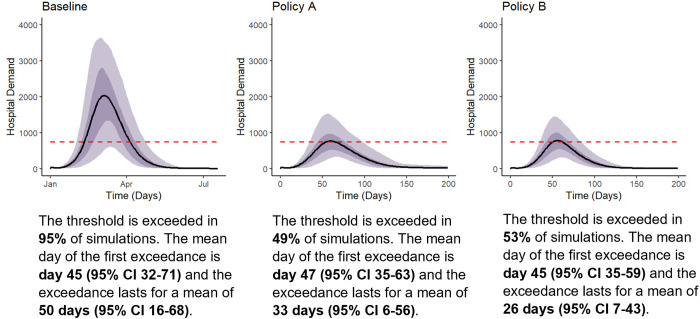
Decision Uncertainty Toolkit: Risk measure and time-outcome fan plots. This figure presents model outputs using the Decision Uncertainty Toolkit, including the time-outcome fan plots. They display the mean (solid line) with 50% credible intervals (dark shading) and 95% credible intervals (light shading) of 1,727 simulations. The decision threshold (or policy objective) is shown with a dashed red line.

Table 2 and Fig 2 present the model outputs using the DUT. In Table 2, we see that Policy B’s expected risk is lower than Policy A’s expected risk, indicating that Policy B is preferred. This is because hospital demand under Policy B peaks and falls more rapidly than under Policy A. As a result, the duration of time when hospital capacity is exceeded is shorter under Policy B. In addition, the cumulative magnitude of exceedances across all simulation runs is lower under Policy B. Therefore, Policy B has a lower expected risk.

**Table 2:** Decision Uncertainty Toolkit outputs: Risk measure.

	Baseline	Policy A	Policy B
**Expected risk**	45,513	3,862	3,267
**Policy risk impact**	–	−91.5%	−92.8%

**Caption:** This table presents model outputs using the Decision Uncertainty Toolkit, including the expected risk and policy risk impact for the baseline scenarios and the policy alternatives (Policy A – close schools and Policy B – mandatory masking).

While the individual expected risk values for each policy may be challenging to interpret independently, a more intuitive understanding can be achieved using a relative comparator. In the second row of Table 2, the policy risk impact compares the risk associated with each policy to the baseline scenario. It is interpreted as the percent change in risk relative to the baseline scenario. Specifically, the risk of exceeding hospital capacity for Policy A and Policy B is reduced by 91.5% and 92.8%, respectively, compared to the baseline scenario. This indicates that Policy B is preferred.

Fig 2 displays time-outcome fan plots. These plots visually illustrate the uncertainty in hospital demand for each policy and facilitate assessment in relation to hospital capacity (the decision threshold). They can be used to assess the probability of hospital demand exceeding capacity and to understand the expected magnitude, timing, and duration of any exceedances. While a direct comparison of the plots in Fig 2 for Policy A and Policy B may not readily identify the preferred policy option, presenting these plots alongside Table 2 provides decision-makers with a quantitative measure of the risk associated with each policy, and a visual representation of that uncertainty for additional context. These plots can be further supplemented with captions describing the uncertainty in the timing and duration of threshold exceedances, as given for Fig 2.

**Fig 3:**
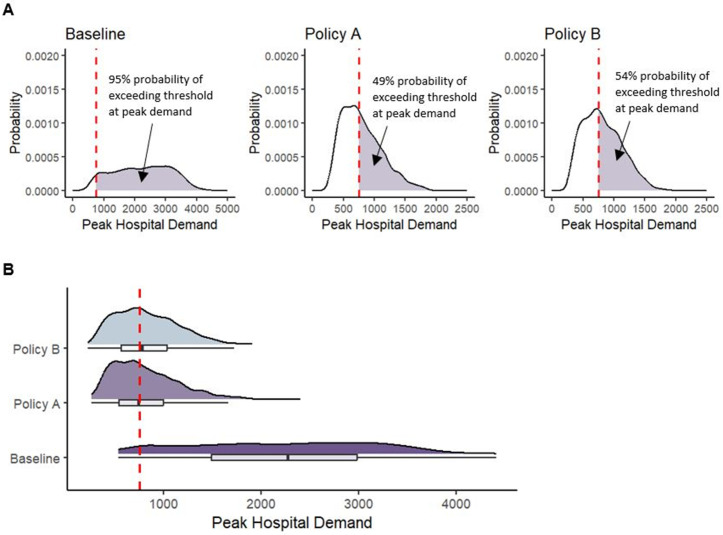
Decision Uncertainty Toolkit outputs: Probability density and raincloud plots. This figure presents model outputs using the Decision Uncertainty Toolkit. Probability density plots of the peak hospital demand are shown in (A) with the shaded area indicating the downside risk. Note the x-axis limits are different for the Baseline and Policy A and B. A raincloud plot showing the probability densities of peak hospital demand, alongside corresponding box plots indicating the mean and 50^th^ percentile range, for each scenario is given in (A). The decision threshold (or policy objective) is indicated using a dashed red line in all figures.

Another consideration for decision-makers is evaluating the severity of the situation at its expected peak. We define the peak as the highest hospital demand observed in each simulation run. Fig 3 represents model outputs using the DUT. Fig 3A shows the probability density plots for hospital demand at its projected peak across all simulation runs for each policy with the shaded area indicating the downside risk. These figures quantify and visually display how likely it is that the hospital demand at its projected peak will exceed hospital capacity, capturing the downside risk associated with the projected peak hospital demand. As shown in Fig 3B, a raincloud plot can complement the probability density plots by showing the same probability densities for each policy alternative alongside corresponding box plots (indicating the mean and 50th percentile range) on a single plot. This presentation offers an intuitive and direct visual comparison of the severity of the situation at its expected peak for each policy alternative.

Fig 3A shows a 49% probability of exceeding hospital capacity at the projected peak for Policy A compared to 54% for Policy B. At the projected peak of hospital demand across all simulation runs, Policy B has a higher probability of exceeding hospital capacity. However, hospital demand under Policy B peaks and falls more rapidly than under Policy A, resulting in a shorter period above capacity. Additionally, the cumulative magnitude of exceedances across all simulation runs is lower under Policy B. Therefore, Policy B has a lower expected risk value, as shown in Fig 1A. While Policy B has a higher downside risk at its peak, it is more favourable than Policy A when considering the expected risk values, given the lower cumulative magnitude of all exceedances across the entire time horizon. Decision-makers must weigh these nuanced risks, in arriving at their decisions, and the DUT provides them with tools to do so effectively.

**Fig 4:**
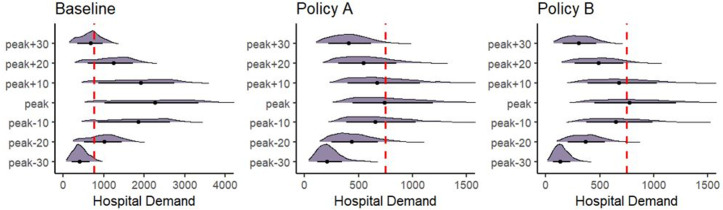
Decision Uncertainty Toolkit outputs: Temporal probability density plots. This figure presents the model outputs using the temporal probability density plots from the Decision Uncertainty Toolkit for the Baseline and Policy A and B. The decision threshold (or policy objective) is shown with a dashed red line.

Decision-makers should also consider how risk changes over the modelled time range. Fig 4 shows the temporal probability density plots for each policy. The probability densities in the center of each plot represent the peak hospital demand (the same as is shown in Fig 2). Above and below the peak probability densities in each plot are the probability densities of hospital demand at different time points relative to the peak. Here, we show the hospital demand every ten days for up to 30 days before and after peak hospital demand. In Fig 4, a comparison of Policy A and Policy B shows that at the peak of hospital demand and 10 days before and after the peak, the preferred policy option is not immediately evident. However, looking at the distributions 20 and 30 days before and after the peak hospital demand, the risk of exceeding hospital capacity with Policy B is lower than with Policy A. This is indicated by the density plots for those time periods sitting further to the left of the policy target. As in Fig 2, Fig 4 also indicates that the risk of exceeding hospital capacity peaks and falls more rapidly with Policy B.

The DUT also includes an R Package ‘DUToolkit’ [25], which provides all required code to generate toolkit outputs, instructions on how to use the toolkit, and standard text descriptions for each toolkit component (Supplementary Material).

## Discussion

We developed the DUT to support both infectious disease modellers and decision-makers in effectively communicating and interpreting decision risk when evaluating multiple policy alternatives. The toolkit extends and contributes to existing methods for communicating infectious disease model results by providing additional key information regarding uncertainty and decision risk. The toolkit consists of: (i) a novel risk measure, which quantitatively captures the downside risk of policy alternatives, and (ii) a series of visuals for communicating uncertainty and decision risk to be used alongside the novel risk measure. We demonstrated the use of the toolkit through a hypothetical example.

We obtained feedback on the DUT through early dissemination at conferences, meetings, and organized workshops, leading to several key improvements. These engagement sessions also highlighted the importance of continuing discussions aimed at addressing the challenges associated with communicating model uncertainty to policymakers and decision-makers. The DUT is intended to be a living repository that can be further developed and expanded based on ongoing feedback and suggestions. We anticipate that the DUT will expand and grow alongside developments in infectious disease modelling and health economics, changes in the needs of decision makers, and through innovation and collaboration with other researchers.

The importance of uncertainty in the evidence base underpinning infectious disease models was highlighted during a rapidly evolving public health crisis such as COVID-19 [1,5,26] leading to increased decision risk, which was not always well communicated using common reporting tools. While common methods of reporting infectious disease model outputs often describe uncertainty visually through uncertainty bands [12–14] use of these visuals alone may not provide decision-makers with a meaningful measure of decision risks, leaving them effectively unaware of critical information when making policy decisions. Including a quantifiable risk measure along with a broad range of data visualizations provides a way for them to assess decision risk by considering the probability and magnitude of undesirable outcomes. This is important in infectious disease modelling, where model results should include uncertainty due to unknowns and/or variability in model inputs.

### Strengths and limitations

The primary advantage of the DUT is its direct alignment with a stated policy objective. Model outputs and decision risks are interpreted and visually represented in relation to this objective, offering a clearer and more meaningful way to communicate uncertainty. This approach ensures that non-technical audiences can interpret the results as part of their decision-making. The risk measure summarizes risk in a single value, measured relative to a stated policy objective. This makes the risk value abstract and challenging to interpret when considering a single policy in isolation. For example, a risk estimate of 3,267 for Policy B in our hypothetical example has little meaning when presented independently. However, presenting risk values for multiple policy alternatives as a percent change in risk relative to a baseline provides a straightforward and meaningful way of communicating risk, particularly for non-technical audiences. This risk measure also ensures the three critical aspects of risk are accurately captured: (i) the probability of the policy alternative not meeting the specified policy objective, (ii) the magnitude of deviations from the stated objective, and (iii) the duration of these deviations.

The toolkit is designed to accommodate a broad range of outputs that are important to both infectious disease modellers and decision-makers, which is an important consideration given the varying purposes and outputs of infectious disease models. However, not all the visual outputs in the toolkit will be ideally suited to all model results. For example, if the results for one or more policy options being compared have highly skewed distributions with very long tails, a raincloud plot may not be ideal as it could obscure focus from the densest parts of the distributions. Adjusting axis limits and adding annotations can help contextualize the graph, but careful consideration should be given to how to communicate these results.

Moreover, infectious disease modellers and decision-makers often have different information needs. Through our engagement workshops, we found that decision-makers were interested in the fan plots and standard descriptions, whereas infectious disease modellers wanted flexibility to extract additional information, such as the probability of exceeding different threshold values and the expected timing of threshold exceedances. We designed the DUT to offer a variety of different outputs in a modular format, allowing users to select the ones that best align with their specific needs. In addition, outputs from the toolkit, such as the probability of exceeding different threshold values and the expected timing of threshold exceedances, can be integrated with other types of charts or tables not included in the toolkit to facilitate communication.

### Considerations for implementation

The consequences associated with different magnitudes and durations of deviations from a stated policy objective are situation-specific and depend on the policies and outcomes being studied. As a result, these cannot be incorporated into a generic risk measure and we caution against using the expected risk values independently. This is important in light of the key assumptions associated with the risk measure as outlined in Box 1, and in particular when the assumptions do not align to the decision maker’s attitudes. Rather, the risk measure should always be interpreted alongside other components of the DUT (such as time outcome fan plots and probability density plots) or other available tools to ensure the full decision risk context is understood.

There are many ways to address situations in which the assumptions outlined in Box 1 do not hold. For example, in situations where the decision maker is sensitive to the distribution of deviations from the threshold *D*_*t*_, the raincloud and temporal density plots can support the assessment of alternatives with different cumulative and temporal risk profiles, beyond the information contained in the risk measure. In cases where the linearity in cumulative risk assumption does not hold, one option would be to calculate the risk measure at different values of *D*_*t*_ and compare values visually or through weighting.

Another consideration is that time preferences may be important when making a policy decision. For instance, a policy that leads to deviations from the threshold sooner might be less favourable than a policy causing deviations later in time, even if the magnitude of the deviations is larger. To incorporate time preference into the risk measure, one approach would be to assign different weights to deviations from the threshold for different time periods. For example, if the risk measure was applied to capture exceedances in the number of infections but an effective antiviral was anticipated to be available in one month, decision makers could choose to weight exceedances in the first month with higher weights than those after one month. However, careful consideration must be given to determining the cutoffs for each time period and the weights to be assigned, in particular if applying discounting for time preference under one year, and considering relative benefits of applying varying threshold values for *D*_*t*_ (which can already vary over time) versus weighting [27].

In infectious disease modelling, different types of model simulations may result in outputs where some model runs are more or less likely than others. Modellers use various methods to account for this, such as calculating a log-likelihood for each simulation run. Incorporating this information into the expected risk calculations may be desirable rather than assuming all simulation runs are equally likely. This can be done by weighting each simulation run within the risk measure.

There may also be more than one outcome that is important to decision-makers. Returning to the example in the previous section, it might be important to consider overall mortality alongside hospital demand when making a policy decision. The expected risk values could be calculated separately for each outcome (i.e., hospital demand and mortality) and policy alternative. This could also be done for different thresholds within the same outcome, for example, a daily hospital census of 750 vs 1000, as suggested above to address concerns around the distribution of deviations from the threshold. These values could then be presented in a single table (see the Supplementary Material for an example). This granular view would enable decision-makers to make transparent trade-offs to decide which outcomes they deem are the most important and choose a policy alternative accordingly. Future work could explore methods for aggregating expected risk values across different outcomes that matter to decision-makers. One approach could be to assign weights to each outcome. However, careful consideration must be given to determining the weights, as they will likely be situation-specific, contingent on the policies and outcomes under consideration, and may differ by decision-maker.

Similarly, decision makers may be interested in spatial comparisons of policy alternatives, for example, where hospital capacity varies within different areas of a larger jurisdiction. In such cases, it may be most appropriate to specify separate models and risk calculations for each of the smaller areas under various scenarios, so that an optimal combination of area-specific policies could be adopted. Future research could expand the DUT to explicitly include spatial factors. Future research could also consider methods to relax the assumptions (distribution neutrality of deviations from *D*_*t*_ and linearity in cumulative risk) specified in Box 1.

A final consideration is that risk values generated by different models should not be directly compared when evaluating policy alternatives. Differences in underlying structures, assumptions, and methods of incorporating empirical evidence and uncertainty can lead to variations in risk values, even for the same baseline scenario [28]. The policy risk impact, calculated as percent changes in risk relative to a baseline scenario can only be meaningfully interpreted across models when policy alternatives are treated similarly in each model.

## Conclusion

We developed the DUT to support both infectious disease modellers and decision-makers in effectively interpreting and communicating decision risk when evaluating multiple policy alternatives. The DUT improves upon common infectious disease model outputs by providing additional outputs regarding uncertainty and decision risk associated with policy alternatives. These outputs empower decision-makers to better evaluate risks when making policy decisions. Improved understanding of decision risk could improve outcomes in future public health crises.

## Supporting information

S1 File Supplemental material. (PDF)
