# Supplementary material for: The decision uncertainty toolkit: Risk measures and visual outputs to support decision making during public health crises
Source: PLoS One. 2025 Oct 1;20(10):e0332522. doi: 10.1371/journal.pone.0332522 (PMC12488006; doi:10.1371/journal.pone.0332522)
Supplement: S1 File — (PDF) [file pone.0332522.s001.pdf]

Decision Uncertainty Toolkit Supplementary Material

Contents

SM1. PDF of DUToolkit Package Website

SM2. Workshop Slide Decks

SM3. Example: Presentation of Expected Risk Across Multiple Outcomes

Note.
Data required to replicate manuscript results can be downloaded at:
https://github.com/IHECA/Decision-Uncertainty-Toolkit

Package website: https://dut.ihe.ca/
CRAN package: https://cran.r-project.org/web/packages/DUToolkit/index.html

DUToolkit

The DUToolkit package provides a suite of tools and visualization for the characterization, estimation, and communication of parameter uncertainty and decision risk. The package is designed to evaluate the impact of policy alternatives on outcomes compared to baseline (i.e., counterfactual analysis), leveraging model outputs from uncertainty analysis.

During public health crises such as the COVID-19 pandemic, decision-makers relied on models to predict and estimate the impact of various policy alternatives on health outcomes. Often, there is a high degree of uncertainty in the evidence base underpinning these models. When there is increased uncertainty, the risk of selecting a policy option that does not align with the intended policy objective also increases; we term this decision risk. Even when models adequately capture uncertainty, the tools used to communicate their outcomes, underlying uncertainty, and the associated decision risk are important to mitigate decisions to adopt sub-optimal policies and/or critical health technologies.

Installation

You can install the 'DUToolkit' package from CRAN with the following command in the console:

```
#> Installing package into 'C:/Users/mwiggins/AppData/Local/Temp/R
#> (as 'lib' is unspecified)
#> Warning: package 'DUToolkit' is not available for this version
#>
#> A version of this package for your version of R might be availa
#> see the ideas at
#> https://cran.r-project.org/doc/manuals/r-patched/R-admin.html#I
```

```
#>                      Baseline Intervention 1
#> Risk                "23078"  "2007"
#> Policy risk impact "-"      "-91%"
```

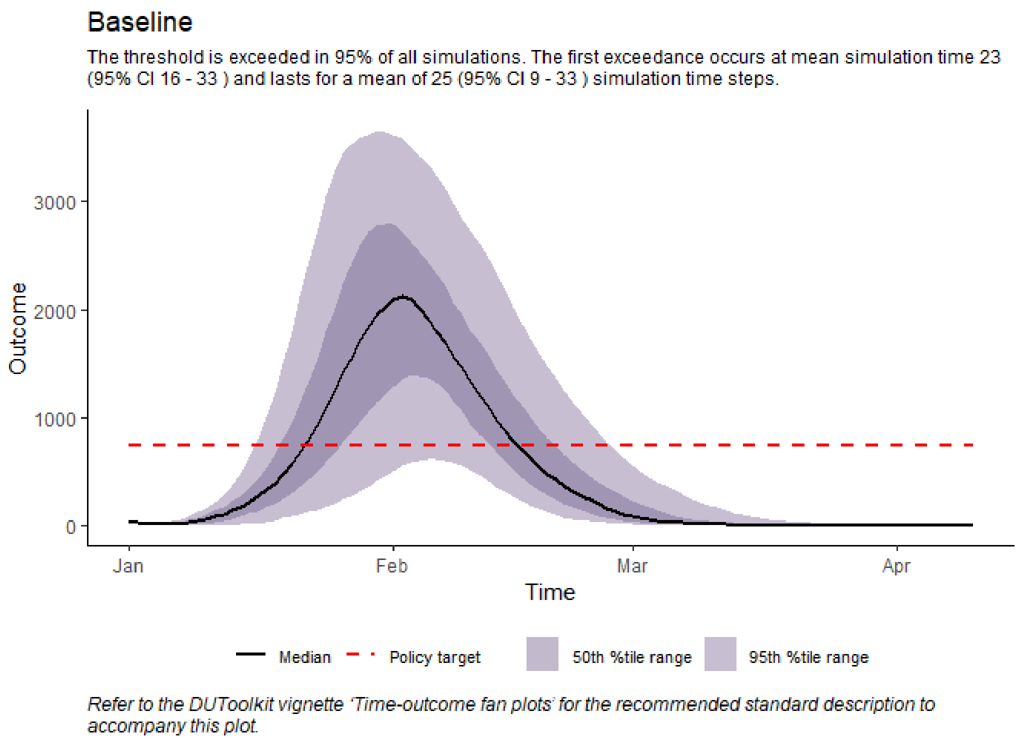


```
#>   N  outcome     i_time
#> 1 1 4207.443 2021-01-26
#> 2 2 1681.521 2021-02-01
#> 3 3 2539.177 2021-02-04
#> 4 4 2969.721 2021-01-31
#> 5 5 3073.741 2021-02-05
#> 6 6 1520.144 2021-02-08
```

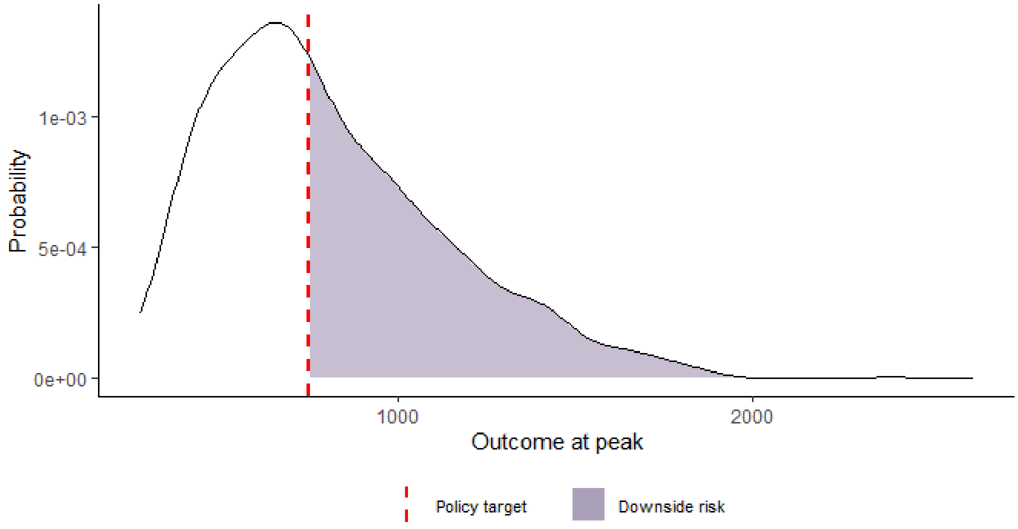

Refer to the DUToolkit vignette 'Probability density plots with risk shading' for the recommended standard description to accompany this plot.

```
#>     750   1000   2000
#> 0.9494 0.8887 0.5895

#>                    Baseline Intervention 1
#> Risk               "1501"   "157"
#> Policy risk impact "-"      "-90%"
```

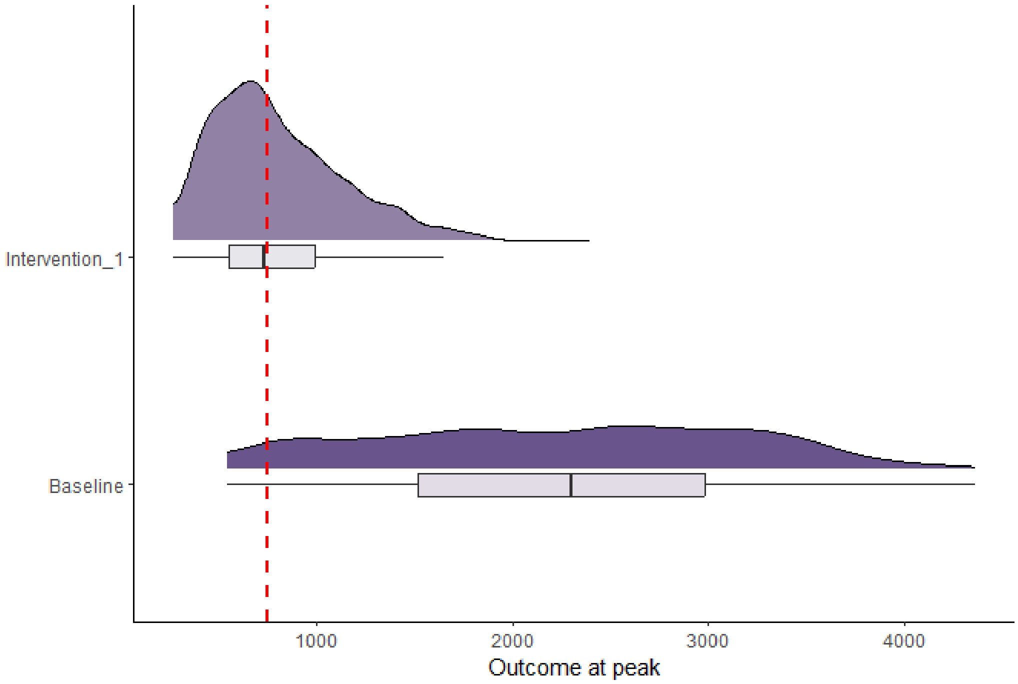

Refer to the DUToolkit vignette 'Raincloud plots' for the recommended standard description to accompany this plot.

```
#> 2 peak 1681.521
#> 3 peak 2539.177
#> 4 peak 2969.721
#> 5 peak 3073.741
#> 6 peak 1520.144
```

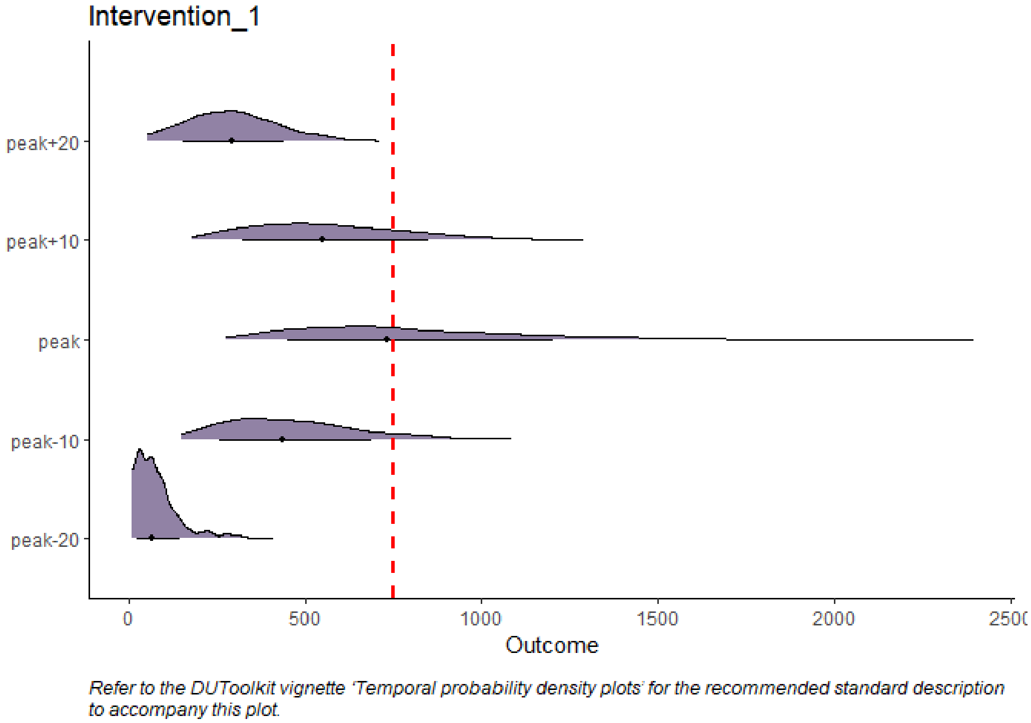


```
#>   time_step   n      q1  median    mean      q3
#> 1   peak-20 813   90.32  136.99  157.21  210.21
#> 2   peak-10 813  738.29 1013.14 1005.52 1260.49
#> 3      peak 813 1520.14 2300.14 2247.85 2982.81
#> 4   peak+10 813  884.80 1246.00 1211.20 1548.80
#> 5   peak+20 813  247.77  326.34  338.76  418.55
```

Acknowledgments

We would like to thank everyone whom we engaged with including workshop participants for their feedback on the Decision Uncertainty Toolkit.

License

Citation

Citing DUToolkit

Developers

Megan Wiggins
Author, maintainer

Marie Betsy Varughese
Author

Ellen Rafferty
Author

Sasha van Katwyk
Author

Christopher McCabe
Author

Jeff Round
Author

Erin Kirwin
Author

Institute of Health Economics
Copyright holder, author

Canadian Network for Modelling Infectious Diseases
Funder

Introduction to DUToolkit

During public health crises such as the COVID-19 pandemic, decision-makers rely on models to predict and estimate the impact of various policy alternatives on health outcomes. Often, there is a high degree of uncertainty in the evidence base underpinning these models. When there is increased uncertainty, the risk of selecting a policy option that does not align with the intended policy objective also increases; we term this decision risk. Even when models adequately capture uncertainty, the tools used to communicate their outcomes, underlying uncertainty, and the associated decision risk are important to mitigate decisions to adopt sub-optimal policies and/or critical health technologies.

The DUToolkit package provides a suite of tools and visualizations for the characterization, estimation, and communication of parameter uncertainty and decision risk. The package is designed to evaluate the impact of policy alternatives on outcomes compared to a pre-defined baseline scenario. The baseline scenario is typically defined as maintaining the status quo or a scenario where no mitigation policies are implemented (i.e. a ‘do nothing’ or ‘existing policy’ scenario). DUToolkit leverages model outputs from uncertainty analysis techniques, such as probabilistic sensitivity analysis, general uncertainty analysis, or Bayesian inference, to support decision-making.

Getting started

The DUToolkit functions fall into five main categories:

- ***Calculating risk:*** includes `calculate_risk()` and `tabulate_risk()`
- ***Time-outcome fan plot:*** includes `plot_fan()` and `calculate_time()`
- ***Probability density plots with risk shading:*** includes `get_max_min_values()`, `plot_density()`, `calculate_threshold_probs()`, and `calculate_max_min_risk()`

`get_relative_values()`, `plot_temporal()`, and `sum_stats_temporal()`

Synthetic data

The DUToolkit package includes pre-loaded synthetic model outputs stored in the R object `psa_data`, which serve as an example dataset. This dataset represents a hypothetical scenario where a decision-maker is selecting between two policies related to COVID-19 in 2020: (i) Baseline – do nothing/current state and (ii) Intervention 1 – close schools. Each policy is expected to impact the number of individuals in the hospital. Hospital capacity has a maximum upper bound, which is the decision threshold.

Data format

The DUToolkit functions require model outputs from multiple simulation runs using different parameter sets (e.g., probabilistic sensitivity analysis, general uncertainty analysis, or Bayesian inference). These outputs must follow a standardized format, as follows:

1. A list of `data.frames` *(Required)*
    - The list must contain one `data.frame` for each policy alternative.
    - Each `data.frame` must have:
        - A **first column** representing model time, either as numeric values, (e.g., 1, 2, 3, …) or as dates in R **Date format** (e.g., 2021-01-01, 2021-01-02, …) with class = “Date”.
        - **Subsequent columns** containing predicted outputs for each simulation run at the corresponding time points (e.g., if there are 100 simulations, there will be 101 columns in the `data.frame`).
    - To ensure a consistent basis for comparison, the model time in the first column should be **identical across all policy alternatives** (i.e., the first column in every `data.frame` should contain the same values).

```
# example data.frame with date in first column
head(psa_data$Baseline[, 1:5])
#>          date        1        2        3        4
#> 2  2021-01-01 37.23075 36.13261 36.62189 36.85947
#> 4  2021-01-02 30.84229 27.20223 28.65276 29.45438
#> 6  2021-01-03 27.77702 21.21132 23.64767 25.18233
#> 8  2021-01-04 29.58525 17.42107 20.96771 23.48235
#> 10 2021-01-05 40.12541 15.36282 20.08010 25.14026
#> 12 2021-01-06 60.30618 14.74307 21.08585 32.24770
```

2. A list of vectors containing weights *(Optional)*

    - Some simulation runs may be more or less likely than others. Various methods can account for this, such as calculating a log-likelihood for each simulation run and converting it into a weight. Users must choose the most appropriate method for their specific scenario.
    - Each **vector** in the list corresponds to a specific policy alternative and contains the **weights** assigned to each simulation run.
    - Each weight vector must have:
        - The **same number of elements** as the number of simulation run columns in the corresponding output `data.frame` (i.e., all columns **except** the first column).
        - The **order of weights** must match the order of simulation run columns in the corresponding `data.frame`.

On this page

Synthetic data

Data format

Infectious Diseases . 2.0.8.

Function reference

Pre-loaded data

`psa_data`
Sample PSA data

Calculating Risk

`calculate_risk()`
Calculate risk measures

`tabulate_risk()`
Output risk measures to table

Time-outcome fan plots

`plot_fan()`
Generate time-outcome fan plots

`calculate_time()`
Calculate time of threshold exceedance

Probability density plots with risk shading

`get_max_min_values()`
Finds peak (or lowest) model output values

`plot_density()`
Generate density plots

`calculate_max_min_risk()`
Calculate risk measures at peak (or minimum)

`calculate_threshold_probs()`
Calculate threshold exceedance probabilities

Generate raincloud plot

Temporal probability density plots

`get_relative_values()`

Find output values at time points relative to the peak (or minimum)

`plot_temporal()`

Generate temporal probability density plots

`sum_stats_temporal()`

Calculates summary statistics at specified time points relative to the peak (or minimum)

On this page

Pre-loaded data
Calculating Risk
Time-outcome fan plots
Probability density plots with risk shading
Raincloud plots
Temporal probability density plots

Calculating Risk

Decisions can be defined via stated policy objectives. In our example the policy objective is to ensure that the number of individuals requiring hospitalization remains below the available hospital capacity. The stated policy objective establishes the criteria for differentiating between policy successes and failures. For example, exceeding hospital capacity is more undesirable than not exceeding it. Therefore, the stated policy objective acts as a threshold against which risks can be evaluated. Risk can be measured in terms of the probability of an undesirable outcome occurring (i.e., exceeding the policy objective), the magnitude of deviations from the stated policy objective, and the duration of these deviations.

Formula

The expected risk for each policy alternative can be estimated using the outputs from multiple model runs with different input parameter sets, as outlined in the box below. Additionally, a weighted version of this formula, which accounts for simulation runs with different weights, is provided.

The expected risk is calculated over a pre-defined time range ($t = t_{\mathrm{min}} \ldots t_{\mathrm{max}}$), which should be selected to capture important features of the decision problem, such as the maximum duration for which any of the policy options being considered might be adopted. The decision threshold (D_{t}) can be either a maximum, such as a maximum hospital capacity, or a minimum, such as a target number of vaccinations to be delivered. The decision threshold (D_{t}) can also have different values for different time periods if, for example, the hospital capacity is expected to change over time. To ensure a consistent basis for comparison, it is important that all decision threshold (D_{t}) values, the total number of simulations (N), and the time range ($t = t_{\mathrm{min}} \ldots t_{\mathrm{max}}$) are consistent across the baseline scenario and each of the policy alternatives being compared.

$$\text{Expected Risk} = \begin{cases} \text{[illegible]} \\ \frac{1}{N}\sum_{n=1}^{N}\sum_{t=t_{\min}}^{t_{\max}}\left(D_t - \min(D_t, O_{nt})\right) & \text{if } D_t \text{ is a minimum} \end{cases}$$

Where $n = 1 \ldots N$ are the number of simulations runs in the analysis, $t = t_{\text{min}} \ldots t_{\text{max}}$ is the simulation time over which the expected risk is calculated, O_{nt} is the model outcome result for simulation run n at time t, and D_{t} is the decision threshold at a given time (the stated policy objective), which can be a maximum or a minimum.

The Expected Risk value calculation implies the following assumptions: (i) ***distribution neutrality of deviations from Dt:*** a certain risk of exceeding Dt by 10 has the same value as a risk distributed equally of an exceedance of either 0 or 20; and (ii) ***linearity in cumulative risk:*** an exceedance of 1 over 100 days has the same value as an exceedance of 10 over 10 days.

Risk measure formula with weights

$$\text{Expected Risk} = \begin{cases} \frac{1}{N}\sum_{n=1}^{N} W_n \times \sum_{t=t_{\min}}^{t_{\max}}\left(\max(D_t, O_{nt}) - D_t\right) & \text{if } D_t \text{ is a maximum} \\ \frac{1}{N}\sum_{n=1}^{N} W_n \times \sum_{t=t_{\min}}^{t_{\max}}\left(D_t - \min(D_t, O_{nt})\right) & \text{if } D_t \text{ is a minimum} \end{cases}$$

Where $n = 1 \ldots N$ are the number of simulations runs in the analysis, $t = t_{\text{min}} \ldots t_{\text{max}}$ is the simulation time, O_{nt} is the model outcome result for simulation run n at time t, D_{t} is the decision threshold at a given time (the stated policy objective), and W_{n} is the weight value for simulation run n.

Calculating risk with DUToolkit

First, we calculate the expected risk for each policy alternative using the `calculate_risk()` function. The policy objective is to ensure that hospital demand remains below the available daily hospital capacity of 750 patients, which defines the decision threshold. We consider a baseline scenario that represented the expected outcome in the absence of any intervention (i.e., taking no action) and a hypothetical policy alternative (Intervention 1 – close schools). We considered a time range of 100 days starting from 2021-01-01, corresponding to the first day of policy implementation. We use the model outputs for 814 simulation runs that incorporate parameter uncertainty for the baseline scenario and Intervention 1.

```
tmax <- max(psa_data$Intervention_1[, 1]) # maximum simulation tim
Dt <- c(rep(750, length(tmin:tmax))) # decision threshold vector
Dt_max <- TRUE # indicates the threshold values are maximums

# calculate risk measure
risk_measures_list <- calculate_risk(psa_data, tmin, tmax, Dt, Dt_

risk_measures_list
#> $Baseline
#> [1] 23078.21
#>
#> $Intervention_1
#> [1] 2007.469
```

The expected risk values for each policy alternative are challenging to interpret independently. A more intuitive understanding can be achieved by using a pre-defined baseline scenario as a comparator and calculating the percent change in risk relative to the baseline scenario. We defined this as the policy risk impact, which can be calculated for each policy alternative using the `tabulate_risk()` function.

```
## generate risk table
risk_table <- tabulate_risk(risk_measures_list,
  n_s = length(risk_measures_list)
)

risk_table
#>                    Baseline   Intervention 1
#> Risk               "23078.21" "2007.47"
#> Policy risk impact "-"        "-91.30%"
```

Sharing outputs

In most cases, the risk table should not be presented alone but should be accompanied by the plots described in the other vignettes to provide additional context.

We also recommend the following standard description for presenting the risk table to decision-makers. We provide the standard description in paragraph and bullet point form for ease of use.

probability of the scenario *exceeding*[1] the specified policy target (i.e., how likely it is), the **magnitude** of the *exceedance*[2] from the target, and the **length of time** the *exceedance*[3] is likely to last. Higher risk values indicate a greater risk that the scenario will not achieve the policy objective.

Interpretation of the risk value is more intuitive using a relative comparator. The policy risk impact in the second row of the table compares the risk associated with each intervention to the baseline scenario. The policy risk impact is interpreted as the percent change in risk relative to the baseline scenario. For example, the expected risk of *exceeding*[4] the policy target in Intervention 1 is reduced by 91.30% relative to the baseline scenario.

Standard description bullet points

The expected risk values in the first row of the table above captures:

- The **probability** of the scenario *exceeding*[5] the specified policy target (i.e., how likely it is).
- The **magnitude** of the *exceedance*[6] from the target.
- The **length of time** the *exceedance*[7] is likely to last.
- Higher risk values indicate a greater risk that the scenario will not achieve the policy objective.

The policy risk impact in the second row of the table:

- Compares the risk associated with each intervention to the baseline scenario.
- It is interpreted as the percent change in risk relative to the baseline scenario.
- For example, the expected risk of *exceeding*[8] the policy target in Intervention 1 is reduced by 91.5% relative to the baseline scenario.

Formula

Calculating risk with DUToolkit

Developed by Megan Wiggins, Marie Betsy Varughese, Ellen Rafferty, Sasha van Katwyk, Christopher McCabe, Jeff Round, Erin Kirwin, Institute of Health Economics, Canadian Network for Modelling Infectious Diseases .

Site built with pkgdown 2.0.8.

Time-outcome fan plots

The DUToolkit includes a series of visual outputs that build upon those used in health economics. These visuals are designed to facilitate the intuitive and direct interpretation of model outputs by infectious disease modellers and decision-makers. The visual and numerical presentation of decision risk are designed as complementary outputs to support decision-makers' deliberations.

Time-outcome fan plots are the first of these visuals. In these plots, the trajectory of the outcome is summarized over time and plotted using the mean value for a given policy alternative. Uncertainty is characterized by shading the 50% and 95% credible intervals (calculated as 25th and 75th percentiles and 2.5th and 97.5th percentiles, respectively). The decision threshold is shown directly on the plot to provide a clear reference point for interpreting the outcome values.

We generate the fan plots for each policy alternative using the `plot_fan()` function.

```
# define inputs
tmin <- min(psa_data$Intervention_1[, 1]) # minimum simulation tim
tmax <- max(psa_data$Intervention_1[, 1]) # maximum simulation tim
Dt <- c(rep(750, length(tmin:tmax))) # decision threshold vector
Dt_max <- TRUE # indicates the threshold values are maximums

# generate fan plots
fan_plots <- plot_fan(psa_data, tmin, tmax, Dt, Dt_max)

## example plot
fan_plots$Baseline
```

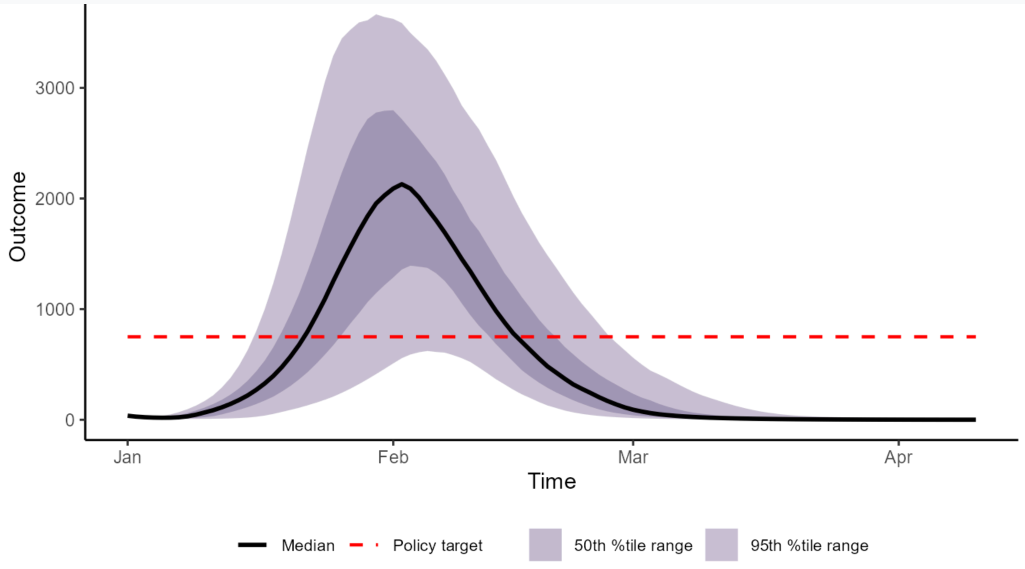

Refer to the DUToolkit vignette 'Time-outcome fan plots' for the recommended standard description to accompany this plot.

All plotting functions in the DUToolkit return ggplot2 objects. You can adjust/customize the plots after they have been generated (ggplot2 cheat sheet).

```
# customize plots
## add fixed y-axis limits and change the label of the y-axis
fan_plots <- lapply(fan_plots, function(x) {
  x + ggplot2::ylim(0, 4000) + ggplot2::labs(y = "Hospital Demand"
})

## remove subtitle and caption
fan_plots <- lapply(fan_plots, function(x) {
  x + ggplot2::labs(subtitle = NULL, caption = NULL)
})

## example plot
fan_plots$Baseline
```

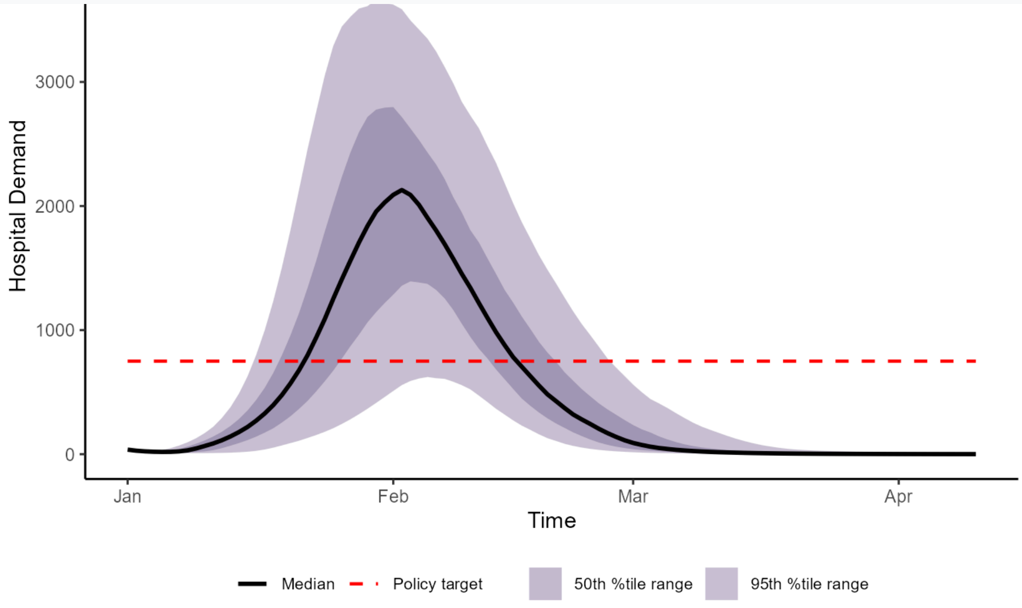

The `calculate_time()` function is used within `plot_fan()` to determine the uncertainty in the timing and duration of threshold exceedances, which are then displayed in the plot subtitle. Additionally, `calculate_time()` can be used independently to generate results in a tabular format without creating fan plots. Its outputs include:

1. Percent of simulations in which the threshold is exceeded (or not met if the threshold is a minimum).
2. Mean simulation time of the first exceedance and 95th percentile range.
3. Mean duration of the first exceedance and 95th percentile range.
4. If the first column of the model output passed to the function is a Date, the mean date of the first and last exceedance.

```
# Find mean and 95%CI of time and duration of first violation of t
time_outcomes_list <- calculate_time(psa_data, tmin, tmax, Dt, Dt_
time_outcomes_list$Baseline
#>     percent_sims time_first 2.5% 97.5% duration_first 2.5% 97.5
#> out    0.9532595   22.88516   16    35       25.03484    9    3
#>      date_last
#> out 2021-02-17
```

hospital demand) for each policy and facilitate assessment in relation to the decision threshold (hospital capacity). They can be used to visually assess the probability of exceeding the threshold and to understand the expected magnitude, timing, and duration of any exceedances. These plots should be presented alongside the risk measures to provide decision-makers with a quantitative measure of the risk associated with each policy, alongside a visual representation of uncertainty for additional context.

We also recommend the following standard description for presenting the time-outcome fan plots to decision-makers. We provide the standard description in paragraph and bullet point form for ease of use.

Standard description

These graphs visually displays the uncertainty surrounding the scenario's **probability** of *exceeding*[1] the specified policy target. They indicate not only the degree of uncertainty but also provides insight into the **magnitude** by which the intervention is likely to *exceed*[2] the target (through percentile shading) and the anticipated **duration** of the *exceedance*[3].

The **magnitude** and the **length of time** that the shaded areas *extends beyond*[4] the policy target (dashed red line) signifies the risk that the scenario will not achieve the policy objective. More shaded area *above*[5] the dashed red line for longer periods indicates a higher risk of not achieving the policy objective.

Standard description bullet points

These graphs visually display:

- The uncertainty surrounding the scenario's **probability** of *exceeding*[6] the specified policy target.
- They also provides insight into the **magnitude** by which the intervention is likely to *exceed*[7] the target (through percentile shading) and the anticipated **duration** of the *exceedance*[8].
- The **magnitude** and the **length of time** that the shaded areas *extends beyond*[9] the policy target (dashed red line) signifies the risk

indicates a higher risk of not achieving the policy objective.

On this page

Standard description

Standard description bullet points

Developed by Megan Wiggins, Marie Betsy Varughese, Ellen Rafferty, Sasha van Katwyk, Christopher McCabe, Jeff Round, Erin Kirwin, Institute of Health Economics, Canadian Network for Modelling Infectious Diseases .

Probability density plots with risk shading

Another important factor for decision-makers is assessing the severity of the situation at its expected peak (or minimum). To do this, we plot the probability density of the highest (or lowest if the threshold is a minimum) projected outcome across simulation runs for a given policy alternative. The decision threshold is shown directly on the plot as a vertical line. The area under the probability density curve where the threshold value is exceeded is shaded to visually display the downside risk of the policy alternative.

In our example, we define the peak as the highest hospital demand observed in each simulation run. First, we find these values using the `get_max_min_values()` function.

```
# define inputs
tmin <- min(psa_data$Intervention_1[, 1]) # minimum simulation tim
tmax <- max(psa_data$Intervention_1[, 1]) # maximum simulation tim
Dt <- c(rep(750, length(tmin:tmax))) # decision threshold vector
Dt_max <- TRUE # indicates the threshold values are maximums

## find peak values
peak_values_list <- get_max_min_values(psa_data, tmin, tmax, Dt_ma

head(peak_values_list$Baseline)
#>   N  outcome     i_time
#> 1 1 4207.443 2021-01-26
#> 2 2 1681.521 2021-02-01
#> 3 3 2539.177 2021-02-04
#> 4 4 2969.721 2021-01-31
#> 5 5 3073.741 2021-02-05
#> 6 6 1520.144 2021-02-08
```

We then use the `plot_density()` function to generate the probability density plots.

```
# calculate risk measure
risk_measures_list <- calculate_risk(psa_data, tmin, tmax, Dt, Dt_

# generate density plots
density_plots <- plot_density(
  peak_values_list, D,
  Dt_max, risk_measures_list
)

## example plot
density_plots$Intervention_1
```

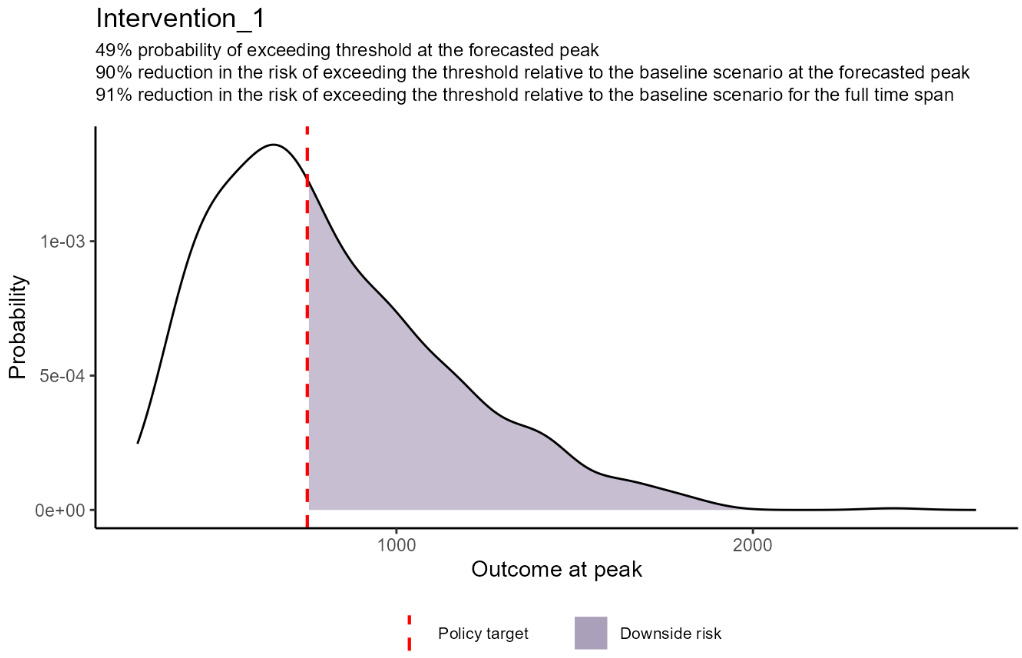

Refer to the DUToolkit vignette 'Probability density plots with risk shading' for the recommended standard description to accompany this plot.

All plotting functions in the DUToolkit return ggplot2 plot objects. You can adjust/customize the plots after they have been generated (ggplot2 cheat sheet).

```
# customize plots
## add fixed x/y-axis limits and change the label of the x-axis
density_plots <- lapply(density_plots, function(x) {
  x + ggplot2::ylim(0, 0.002) + ggplot2::xlim(0, 4500) +
    ggplot2::labs(x = "Hospital demand at peak")
})
```

```
  x + ggplot2::labs(subtitle = NULL, caption = NULL)
})

## example plot
density_plots$Intervention_1
```

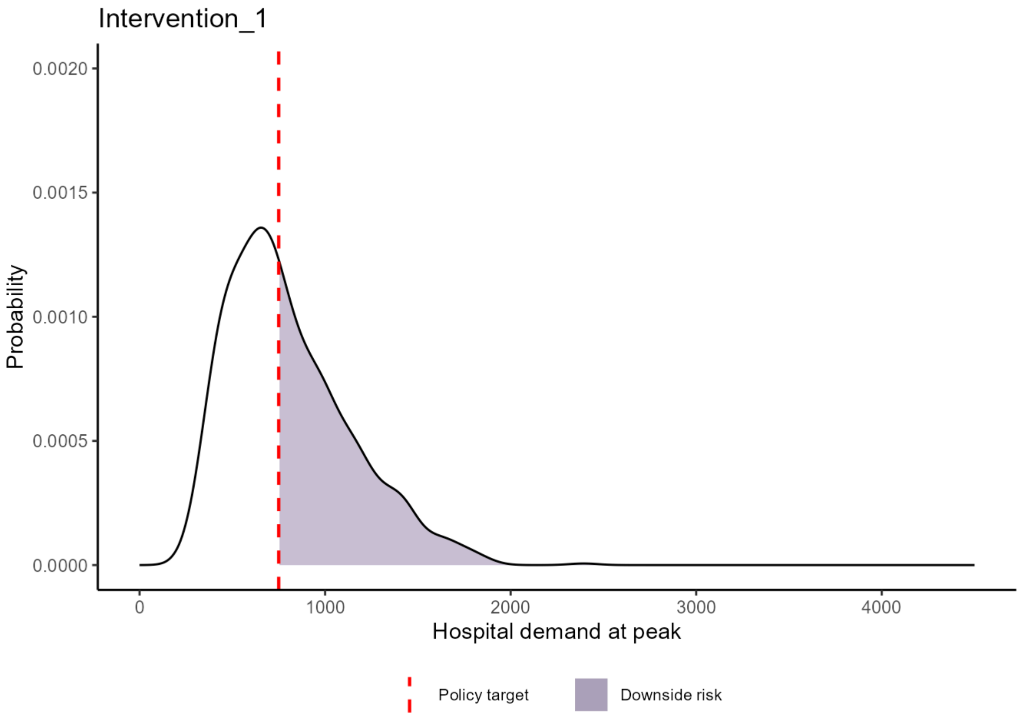

The `calculate_max_min_risk()` function is used within `plot_density()` to calculate the risk measures at the peak values (or lowest values if the threshold is a minimum), which is then displayed in the plot subtitle. Additionally, `calculate_max_min_risk()` can be used independently to generate results without creating density plots.

```
# calculate risk measures at peak values
peak_risk <- calculate_max_min_risk(peak_values_list, D, Dt_max)

# generate risk table dataframe
peak_risk_table <- tabulate_risk(peak_risk, n_s = length(peak_risk
peak_risk_table
#>                       Baseline  Intervention 1
#> Risk                  "1500.79" "156.52"
#> Policy risk impact "-"       "-89.57%"
```

threshold(s) using a Riemann sum approach.

```
# define vector of threshold values
Dp <- c(750, 1000, 2000)

# calculate probability that peak value is > specified threshold v
peak_probs <- calculate_threshold_probs(peak_values_list, Dp, Dt_m

peak_probs$Baseline
#>    750   1000   2000
#> 0.9494 0.8887 0.5895
```

Sharing outputs

The proability density plots further supplement the risk table and the time-outcome fan plots by quantifying and visually displaying how likely it is that the outcome at its projected peak will exceed the threshold, capturing the downside risk associated with the projected peak outcome.

We also recommend the following standard description for presenting the probability density plots to decision-makers. We provide the standard description in paragraph and bullet point form for ease of use.

Standard description

These probability density graphs show the distribution of the *highest*[1] forecasted *outcome*[2] (i.e., the *peak*[3]). The red dashed line indicates the policy target. The shaded area indicates how likely it is that the *outcome*[4] at its forecasted *peak*[5] will *exceed*[6] the policy target, or simply, the amount of downside risk. A larger shaded area means more downside risk.

Standard description bullet points

These probability density graphs show:

- The distribution of the *highest*[7] forecasted *outcome*[8] (i.e., the *peak*[9]).
- The red dashed line indicates the policy target.
- The shaded area indicates how likely it is that the *outcome*[10] at its forecasted *peak*[11] will *exceed*[12] the policy target (i.e, the amount of

On this page

Standard description

Standard description bullet points

Developed by Megan Wiggins, Marie Betsy Varughese, Ellen Rafferty, Sasha van Katwyk, Christopher McCabe, Jeff Round, Erin Kirwin, Institute of Health Economics, Canadian Network for Modelling Infectious Diseases .

Raincloud plots

The probability densities of the highest (or lowest if the threshold is a minimum) projected outcome across simulation runs are plotted for each policy alternative alongside corresponding box plots, which indicate the mean and 50th percentile range. These plots are presented collectively on a single graph to facilitate visual comparison of the policy alternatives. The decision threshold is shown directly on the plot as a vertical line to provide a clear reference point for interpreting the outputs.

We use the `plot_raincloud()` function to generate the raincloud plots.

```
# define inputs
tmin <- min(psa_data$Intervention_1[, 1]) # minimum simulation tim
tmax <- max(psa_data$Intervention_1[, 1]) # maximum simulation tim
Dt_max <- TRUE # indicates the threshold values are maximums
D <- 750 # single threshold value for the peak

## find peak values
peak_values_list <- get_max_min_values(psa_data, tmin, tmax, Dt_ma

# generate raincloud plot
raincloud_plot <- plot_raincloud(peak_values_list, D)

raincloud_plot
```

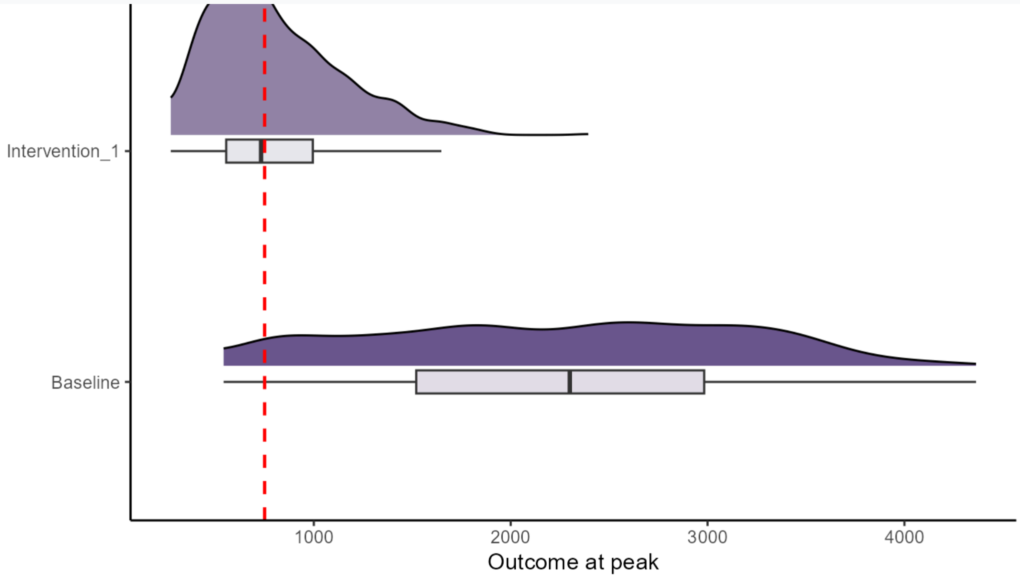

Refer to the DUToolkit vignette 'Raincloud plots' for the recommended standard description to accompany this plot.

Sharing outputs

The raincloud plot can supplement the probability density plots by providing an intuitive and direct visual comparison of the severity of the situation at its expected peak for each policy alternative on a single plot.

We also recommend the following standard description for presenting the raincloud plots to decision-makers. We provide the standard description in paragraph and bullet point form for ease of use.

Standard description

This graph shows the distribution of the *highest*[1] forecasted *outcome*[2] (i.e., the *peak*[3]) for each scenario alongside a corresponding box plot which indicates the mean and 50th percentile range. All interventions are displayed on a single graph to allow for visual comparison.

The red dashed line indicates the policy target. The more distribution area located to the *right*[4] of the policy target line, the greater the probability that the *outcome*[5] at its forecasted *peak*[6] will *surpass*[7] the policy target.

Standard description bullet points

This graph shows:

- A corresponding box plot for each scenario which indicates the mean and 50th percentile range.
- The red dashed line indicates the policy target.
- The more distribution area located to the *right*[11] of the policy target line, the greater the probability that the *outcome*[12] at its forecasted *peak*[13] will *surpass*[14] the policy target.

On this page

Standard description

Standard description bullet points

Temporal probability density plots

Decision-makers may also want to consider how risk changes over the modelled time range. To do this, we plot the probability densities over time. The probability density of the highest (or lowest if the threshold is a minimum) projected outcome across simulation runs is plotted in the center of the graph for a given policy alternative. Above and below, the probability density of the outcome at specified time points relative to the time of the highest (or lowest) project outcome is plotted to visually illustrate how uncertainty, and therefore risk, changes over time. The decision threshold is shown directly on the plot as a vertical line to provide a clear reference point for interpreting the outputs.

First, we find the model output value at the specified time points relative to the peak value for each simulation run using the `get_relative_values()` function. In our example, we will find the hospital demand every ten days for up to 20 days before and after the peak hospital demand.

```
# define inputs
tmin <- min(psa_data$Intervention_1[, 1]) # minimum simulation tim
tmax <- max(psa_data$Intervention_1[, 1]) # maximum simulation tim
Dt_max <- TRUE # indicates the threshold values are maximums
D <- 750 # single threshold value for the peak
t_s <- 20 # total number of time steps from the peak
t_ss <- 10 # time step increments to move in

## find peak values
peak_values_list <- get_max_min_values(psa_data, tmin, tmax, Dt_ma

# find values for temporal density plots
peak_temporal_list <- get_relative_values(psa_data, peak_values_li

head(peak_temporal_list$Baseline[[1]])
#>   time  outcome N
#> 1 peak 4207.443 1
#> 2 peak 1681.521 2
```

```
#> 6 peak 1520.144 6
```

We then use the `plot_temporal()` function to generate the temporal probability density plots.

```
# generate peak temporal density plots
peak_temporal_plots <- plot_temporal(peak_temporal_list, D)

## example plot
peak_temporal_plots$Baseline
```

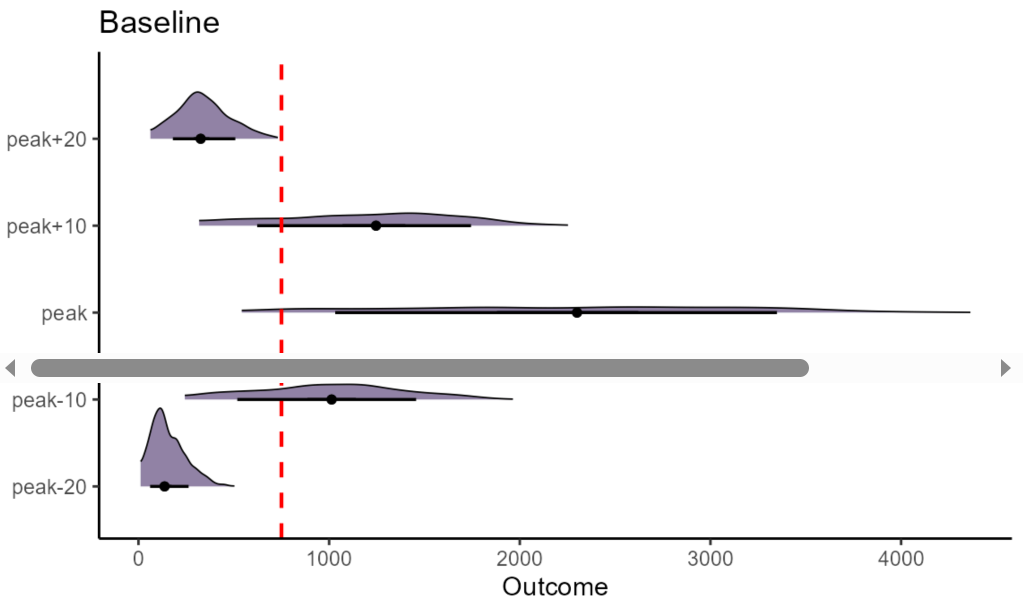

Refer to the DUToolkit vignette 'Temporal probability density plots' for the recommended standard description to accompany this plot.

The `sum_stats_temporal()` function can be used to calculate summary statistics (n, mean, median, and IQR) for the model output values at the time step relative to the peak value.

```
# generate summary statistics for peak temporal data
stats_peak_temporal <- sum_stats_temporal(peak_temporal_list)

stats_peak_temporal$Baseline
#>   time_step   n      q1  median    mean      q3
#> 1   peak-20 813   90.32  136.99  157.21  210.21
#> 2   peak-10 813  738.29 1013.14 1005.52 1260.49
#> 3      peak 813 1520.14 2300.14 2247.85 2982.81
```

Sharing outputs

The probability density plotted in the center of the peak temporal plot (labelled 'peak') is the same probability density that is plotted with `plot_density()` function and `plot_raincloud()` function. The temporal probability density plots can further supplement these probability density plots by showing how uncertainty changes over time for each scenario.

We also recommend the following standard description for presenting the temporal probability density plots to decision-makers. We provide the standard description in paragraph and bullet point form for ease of use.

Standard description

These graph visually illustrates how uncertainty changes over time by showing the distributions of the forecasted *outcome*[1] over time. The distribution in the center of the plot represents the *peak*[2] *outcome*[3]. The distributions above and below show the outcome at different time points relative to the *peak*[4], both forward (above) and backward (below) in time.

The red dashed line indicates the policy target. A greater area to the *right*[5] of this line means a higher risk of *outcome*[6] *exceeding*[7] the policy target at the specified time. Scenarios where the area to the *right*[8] of the policy target is more quickly reduced as you move away from the *peak*[9] result in a shorter periods of time at higher risk of not achieving the policy target.

Standard description bullet points

These graphs visually illustrates how uncertainty changes over time:

- The distribution in the center of the plot represents the *peak*[10] *outcome*[11].
- The distributions above and below show the outcome at different time points relative to the *peak*[12], both forward (above) and backward (below) in time.
- The red dashed line indicates the policy target.

- Scenarios where the area to the *right*[16] of the policy target is more quickly reduced as you move away from the *peak*[17] result in a shorter time frame of higher uncertainty.

On this page

Standard description

Standard description bullet points

SM2. Workshop Slide Decks

The Decision Uncertainty Toolkit – Modeler Workshop

February 9, 2024

Institute of Health Economics

Megan Wiggins, Marie Betsy Varughese, Ellen Rafferty, Jeff Round, Sasha van Katwyk, Erin Kirwin

Thank you to Nicole Oak for helping us organize this workshop!

IHE INSTITUTE OF HEALTH ECONOMICS ALBERTA CANADA

Funding Acknowledgements

- Canadian Network for Modelling Infectious Diseases (CANMOD)
- One Society Network

CANMOD
Canadian Network for Modelling Infectious Diseases
Réseau canadien de modélisation des maladies infectieuses
Strengthening the community of people who are making and using infectious disease models to improve public health both inside and outside of Canada

One Society Network
Interdisciplinary Pandemic Impact Modelling

Agenda

Time	Agenda Item	Lead
8:00 – 8:10 AM MT	Welcome & Introductions	Betsy
8:10 – 8:20	Icebreaker Activity	
8:20 – 8:35	Overview of the toolkit	
8:35 – 8:55	Toolkit content (i.e., risk measures and graphical components)	
8:55 – 9:10	Coding (i.e., using sample data and your own readme file)	
9:10 – 9:45	Trying out the toolkit with participant presentations (10-15 mins per person)	All
9:45 – 9:55	Descriptions and other information	
9:55 – 10:00	Wrap-up	Betsy

Introductions & Ice-Breaker Activity (10 min)

- Your name and role?

- What are your areas of expertise/interest/research background?

- What is your favorite food and why?

Overview of the Toolkit (15 min)

- The Aim: To characterize, visualize, and communicate decision risk for infectious disease models

- ‘Decision Uncertainty Toolkit’ developed through engagement with infectious disease modellers and decision makers
  - Visualization tools
  - New measures of risk
  - R bookdown document with standard description text and codes (living repository)

Working Draft Shared on GitHub

Models were an important part of decision making during the COVID-19 response internationally

- The explicit use models for decision making on such a large scale.
- Huge opportunity for evidence-informed decision making.
- Not all decision makers were prepared to interface with modellers, and vice-versa.
- When there are gaps in the communication of model assumptions and uncertainty, the results of models are difficult to interpret for decision makers.

Why are we doing this? The Problem

- Means do not provide information about outcome uncertainty and therefore risk.
- Important for skewed distributions such as cases, hospitalizations, or deaths.
- Essentially, decision makers can be unaware of the risk associated with alternative policy options, and/or without tools to consider this risk.

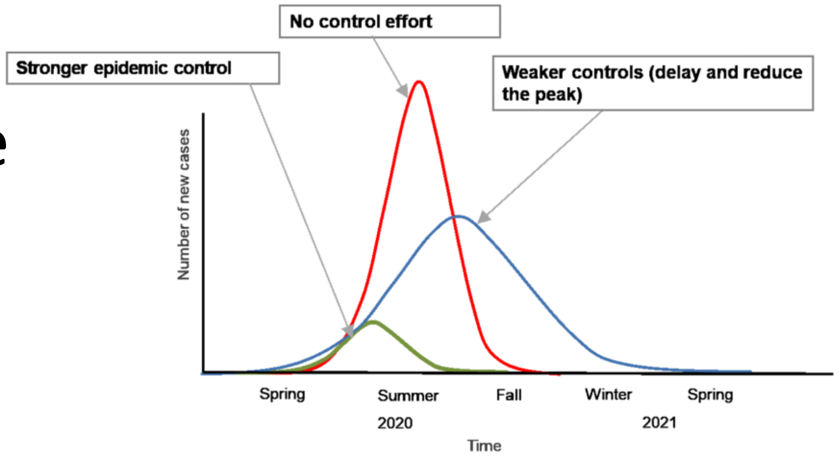

https://www.canada.ca/en/public-health/services/publications/diseases-conditions/covid-19-using-data-modelling-inform-public-health-action-april-28-2020.html

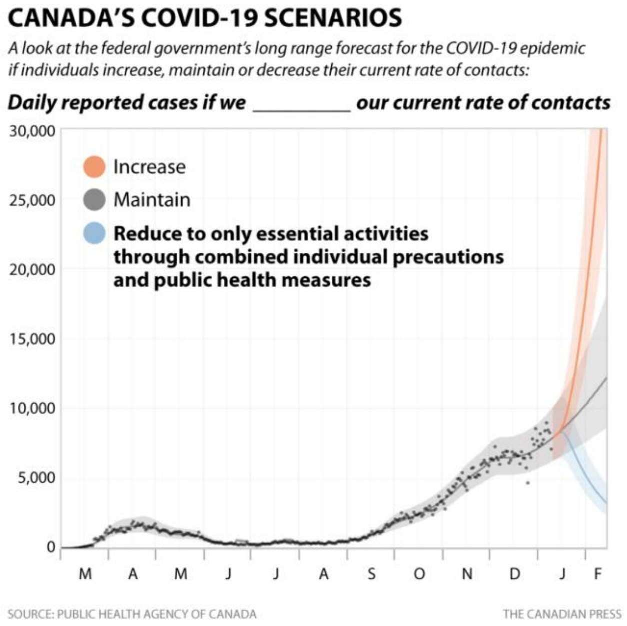

https://www.cbc.ca/news/politics/phac-modelling-covid19-1.5874530

Some Types of Modeling Uncertainty

- Structural assumptions i.e., SIR or SEIR , how many vaccine compartments? - model selection methods (Portet, 2020)

- Parameter assumptions: i.e., one-way sensitivity, probabilistic sensitivity analysis or PSA, partial rank correlation coefficient (PRCC) methods etc. of known, unknown, and/or estimated parameters

- Decision uncertainty: e.g., scenarios, what should we do? i.e., mean trajectory of scenario A is under a threshold, but 95% UCL is high versus scenario B with a trajectory that is just over the threshold, but 95% UCL is not as high compared to scenario A?

Decision Uncertainty Toolkit

- Visualizations
- Risk Measure
- Descriptions (or Interpretations)

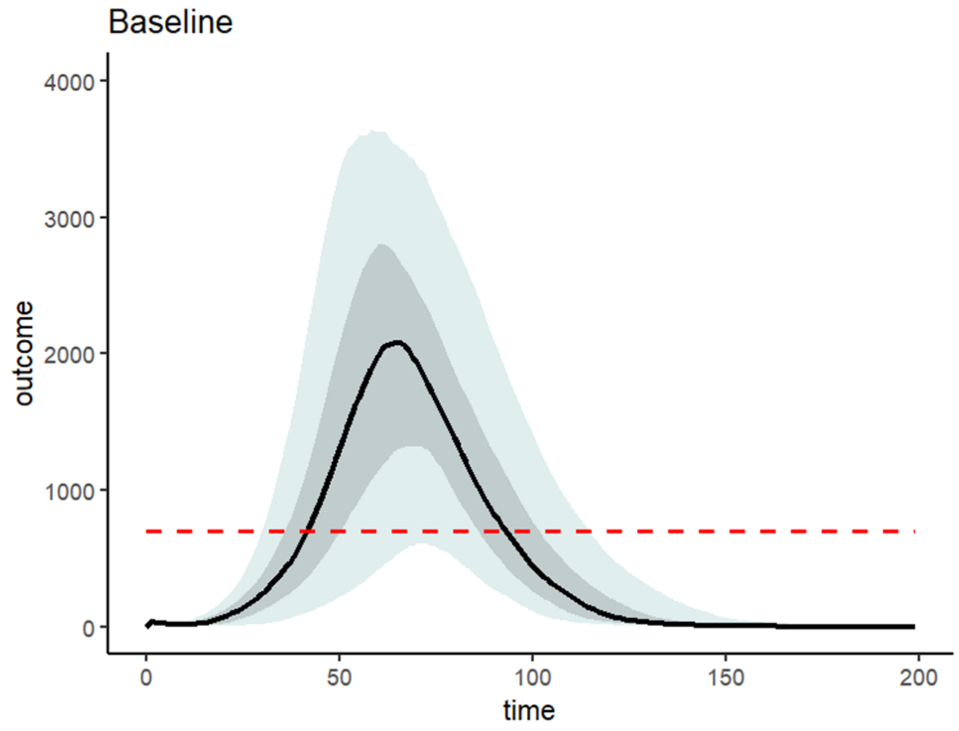

Risk measure formula

For each scenario the expected risk measure is calculated as follows:

$$Expected\ Risk = \begin{cases} \dfrac{\sum_{n=1}^{N} W_n \times \sum_{t=t_{min}}^{t_{max}} (\max(D_t, O_{nt}) - D_t)}{N} & if\ D_t\ is\ a\ maximum \\ \dfrac{\sum_{n=1}^{N} W_n \times \sum_{t=t_{min}}^{t_{max}} (D_t - \min(D_t, O_{nt}))}{N} & if\ D_t\ is\ a\ minimum \end{cases}$$

IHE INSTITUTE OF HEALTH ECONOMICS ALBERTA CANADA

Part 1: Visualizations

Example output:

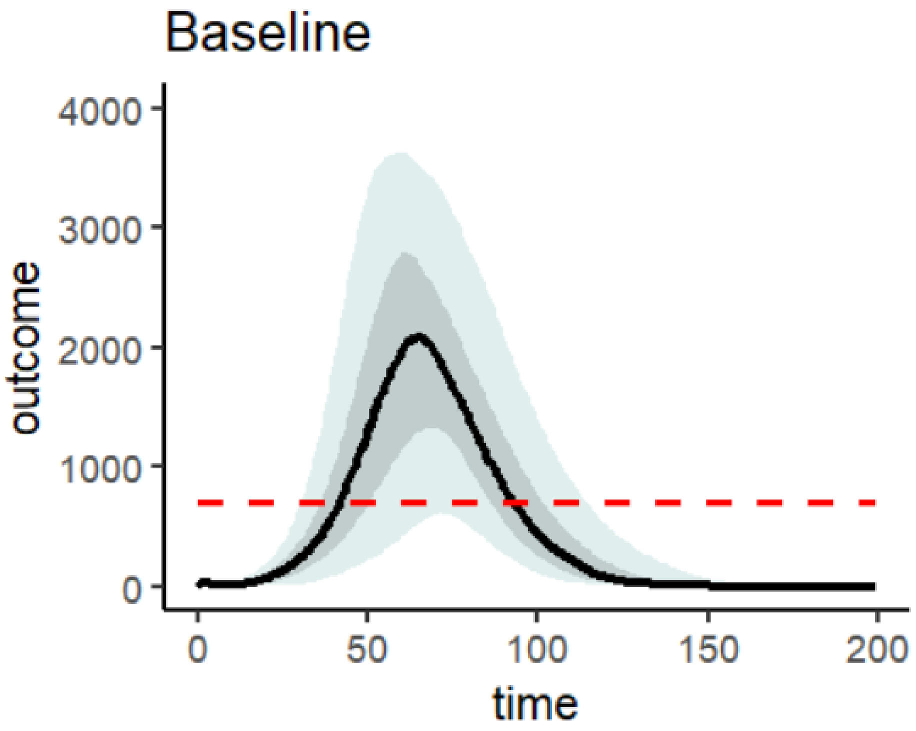

Example output:

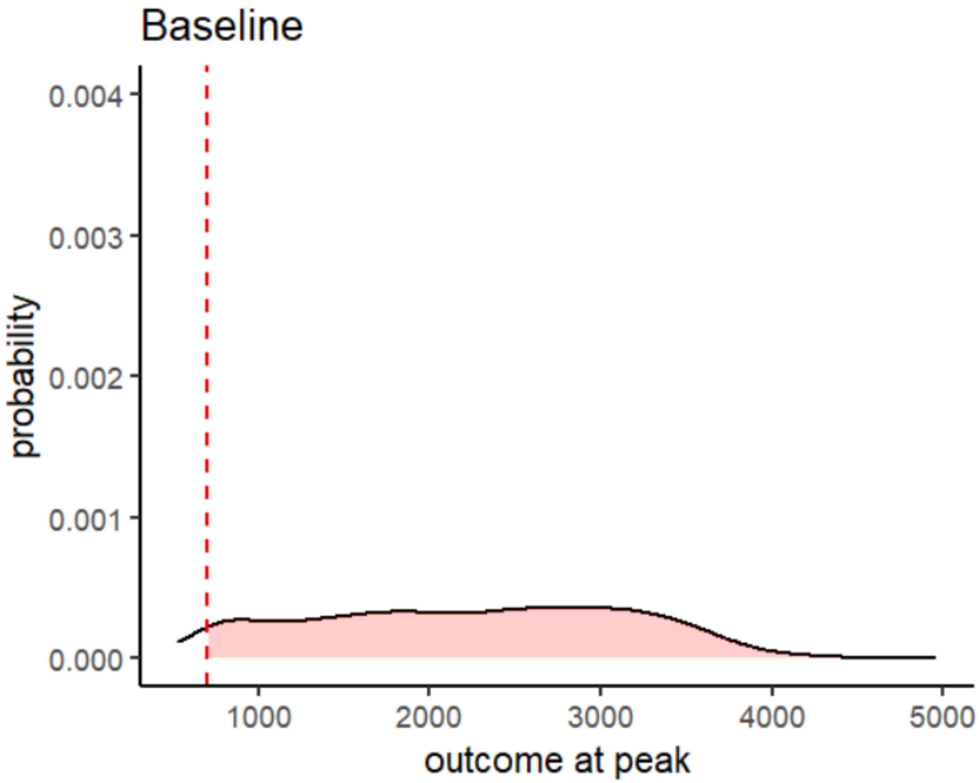

Part 2: Risk Measures I

Risk measure formula

For each scenario the expected risk measure is calculated as follows:

$$Expected\ Risk = \begin{cases} \frac{\sum_{n=1}^{N} W_n \times \sum_{t=t_{min}}^{t_{max}} (\max(D_t, O_{nt}) - D_t)}{N} & if\ D_t\ is\ a\ maximum \\ \frac{\sum_{n=1}^{N} W_n \times \sum_{t=t_{min}}^{t_{max}} (D_t - \min(D_t, O_{nt}))}{N} & if\ D_t\ is\ a\ minimum \end{cases}$$

Where:

- *n = 1…N* is the number of simulation runs
- $t = t_{min} \ldots t_{max}$ is the simulation time
- O_{nt} is the observed outcome for simulation run *n* at time *t*
- D_t is the decision threshold at time *t*
- W_n is the weight assigned to simulation run *n*

Note: *If Dt is a maximum decision threshold, the expected risk indicates the risk of* ***exceeding*** *the threshold value; If Dt is a minimum decision threshold, the expected risk indicated the risk of* ***falling under*** *the threshold value.*

- Work with decision makers to define policy thresholds that can be used to measure risk **across scenarios**
- Quantify distance from threshold and probability of exceeding the threshold (i.e., the first formula)
- Risk evaluated for a specified time interval

Part 2: Risk Measures II

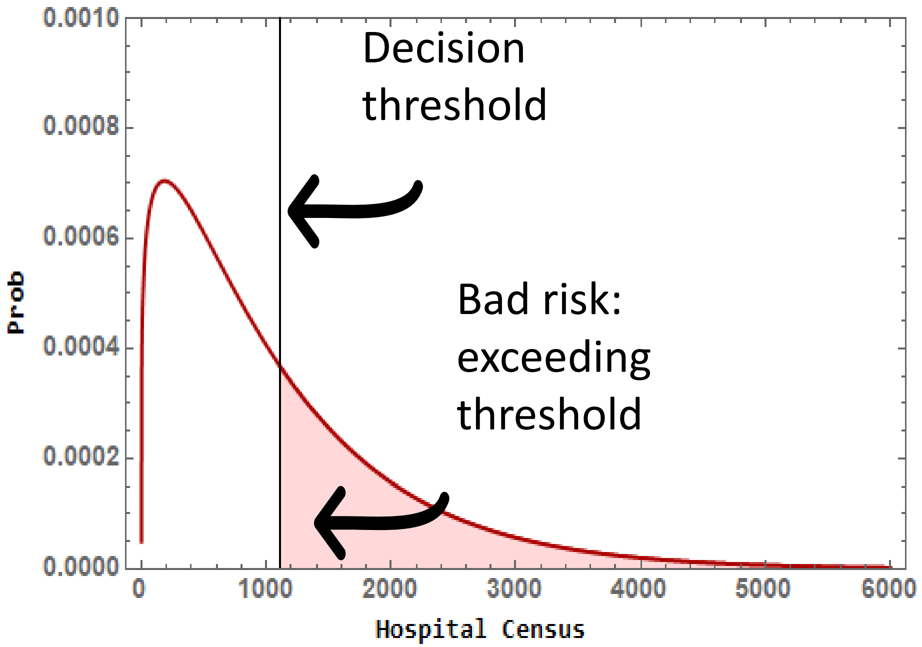

- Interpretation of risk value is easier with a relative comparator (ex. what does a value of 370 mean?)
  - Define a 'baseline' comparator and calculate relative values
  - Ex. baseline risk is 500, scenario risk is 370
  - (370-500)/500 = -0.26, **so risk is reduced by 26%**

Part 3: Descriptions

Descriptions of toolkit elements

- Descriptions of toolkit elements
- Descriptions of approaches to uncertainty
  - Standard descriptions and examples
  - Aim to use common or example language that has been supported by decision makers and modellers through engagement

From GitHub:

Standard description text for risk table

We recommend the following standard description for presenting the risk table above to decision makers:

The expected risk values in the first row of the table above captures the probability of the intervention surpassing the specified policy target (i.e., how likely it is), the magnitude of the exceedance from target, and the length of time the exceedance is likely to last. Higher risk values indicate a greater risk that the intervention will not meet the policy objective.

Interpretation of the risk value is more intuitive using a relative comparator. The policy risk impact in the second row of the table compares the risk associated with each intervention to the baseline scenario. The policy risk impact is interpreted as the percent change in risk relative to the baseline scenario. For example, the expected risk of exceeding the policy target in Intervention 1 is reduced by 90.0% relative to the baseline scenario.

Example: Decision Scenario

Decision maker is selecting between multiple policies:

A. Baseline – do nothing
B. Intervention 1 – e.g., close schools
C. Intervention 2 – e.g., mandatory masking
D. Intervention 3 – e.g., close schools + mandatory masking

Policy target: keep hospitalizations under 700 (capacity maximum)

Note: All these graphs are synthetic simulations and used for illustration purposes only

Decision Uncertainty Toolkit Example Plots

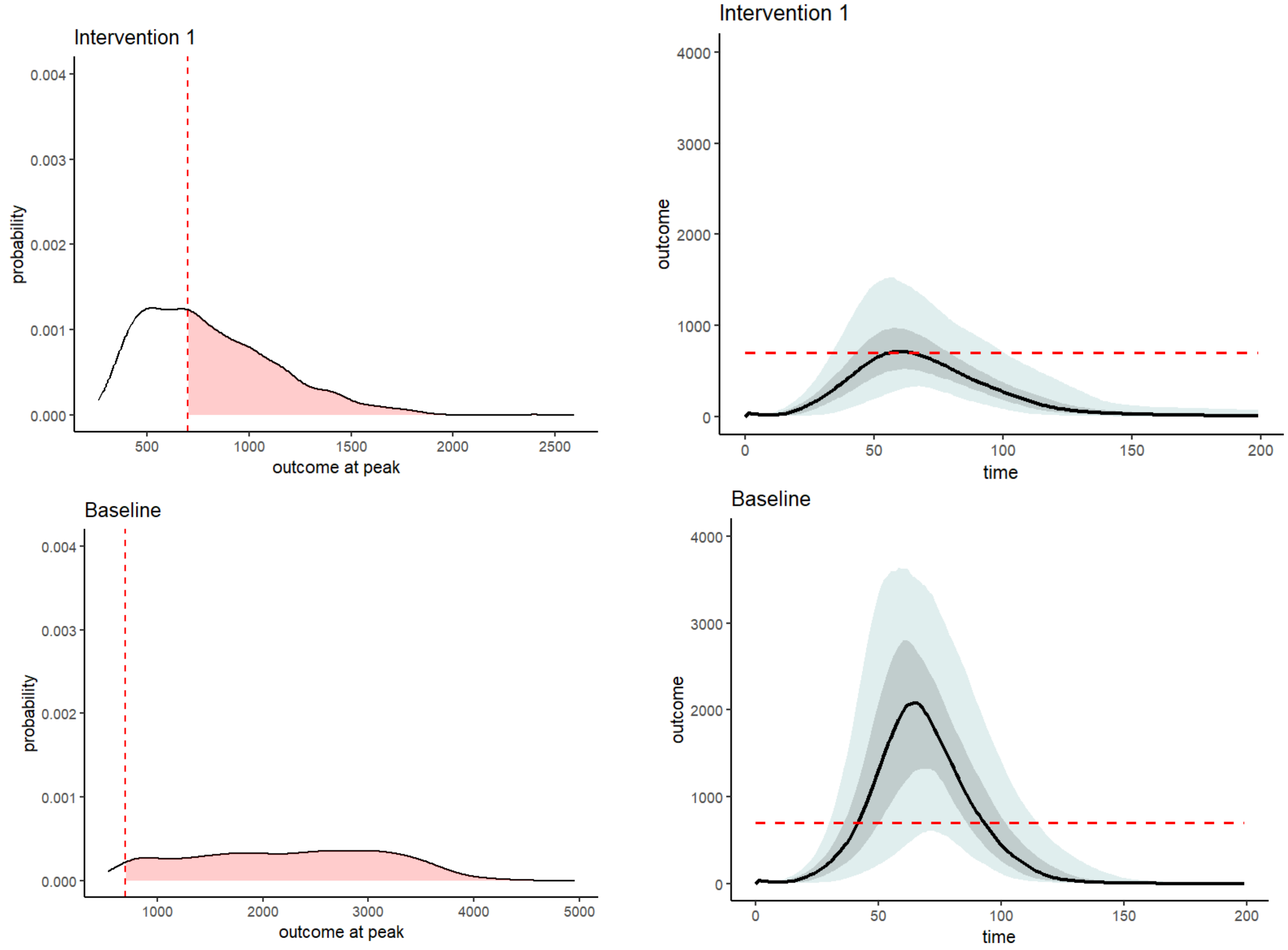

IHE INSTITUTE OF HEALTH ECONOMICS ALBERTA CANADA

Decision Uncertainty Toolkit Example Plots

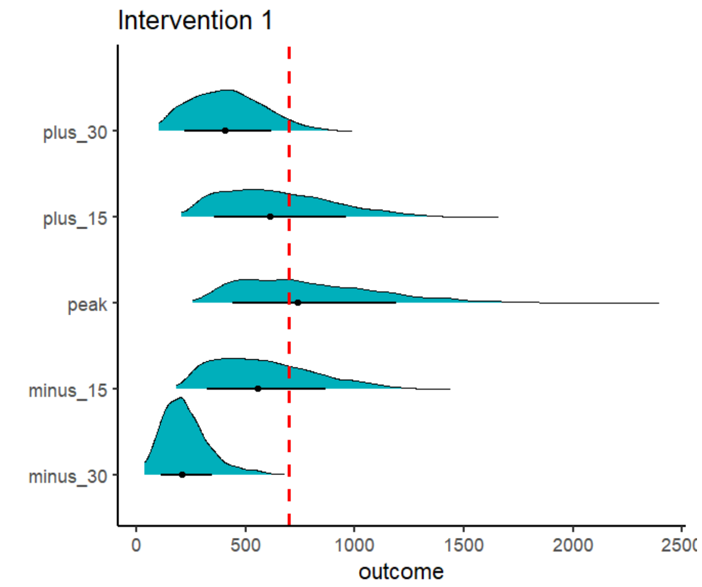

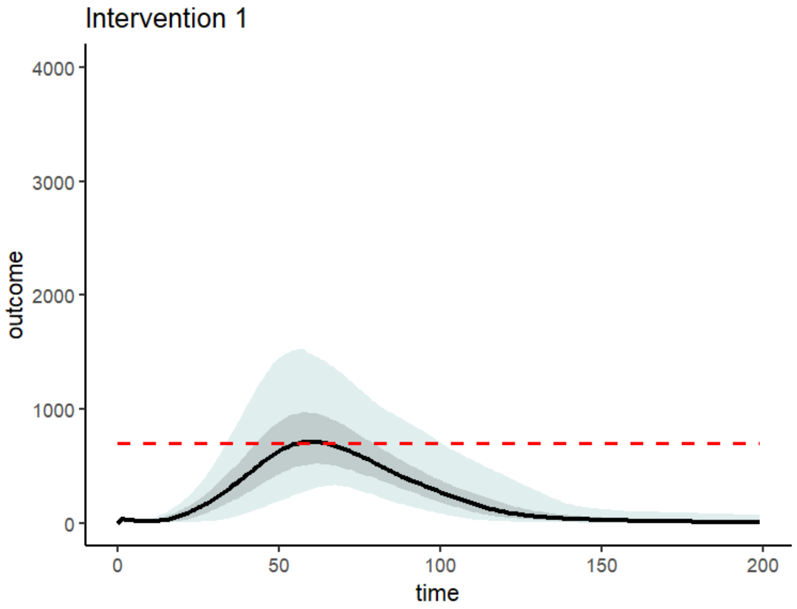

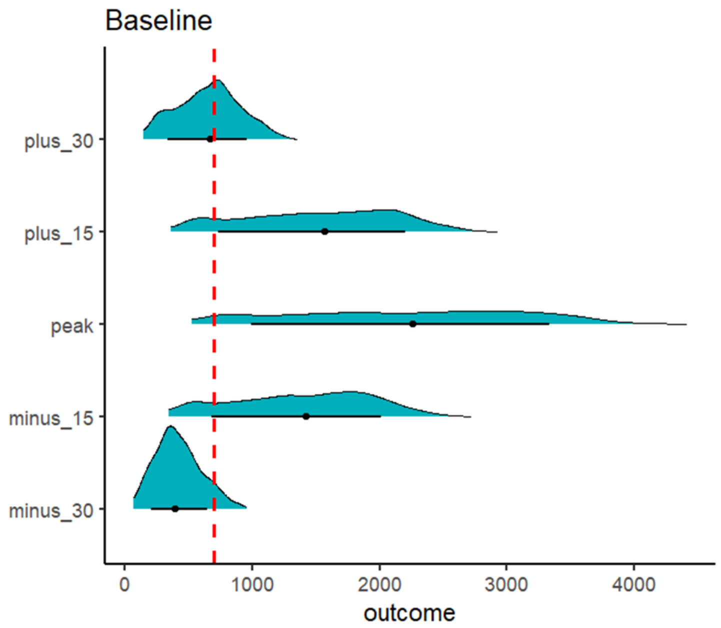

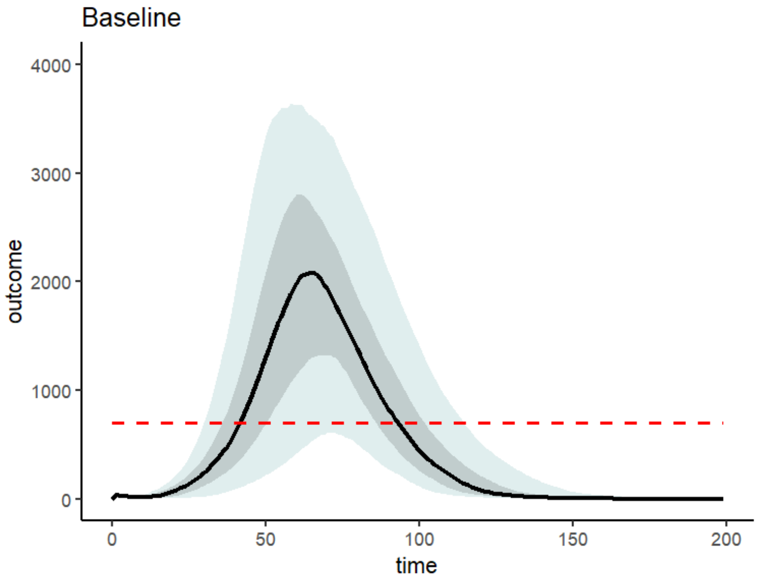

Decision Uncertainty Toolkit: Expected Risk

	Baseline	Intervention 1	Intervention 2	Intervention 3
Expected risk	47,661	4,777	4,035	1,374
Policy risk impact	-	-90%	-92%	-97%

- Time range: 0 to 199 days
- Relative comparison to baseline model (no intervention)
- Interpretation of expected risk is relative to baseline
- e.g., for Intervention 1 - (4777 – 47661)/47661 = -90%

Decision Uncertainty Toolkit: Expected Risk

	Baseline	Intervention 1	Intervention 2	Intervention 3
Risk	47,661	4,777	4,035	1,374
Policy risk impact	-	-90%	-92%	-97%

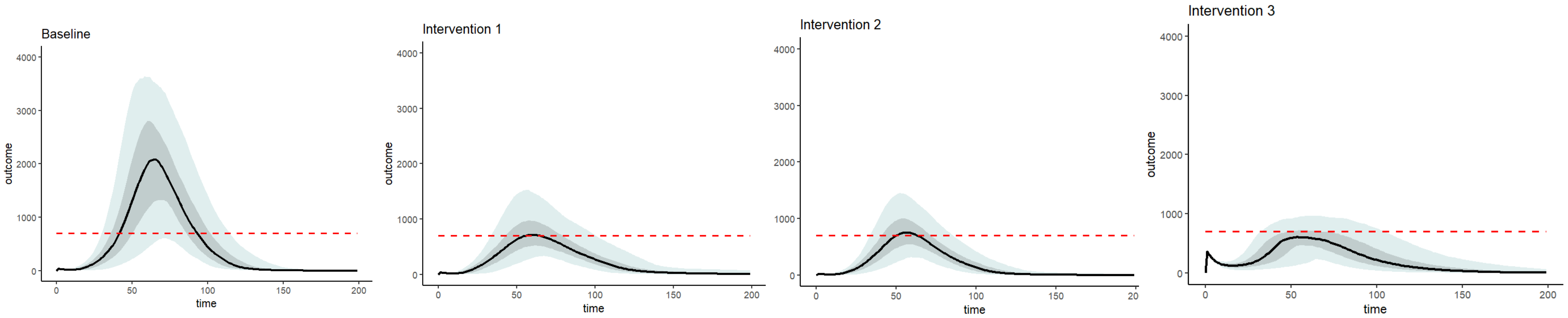

Decision Uncertainty Toolkit: Expected Risk

Decision Threshold = 700

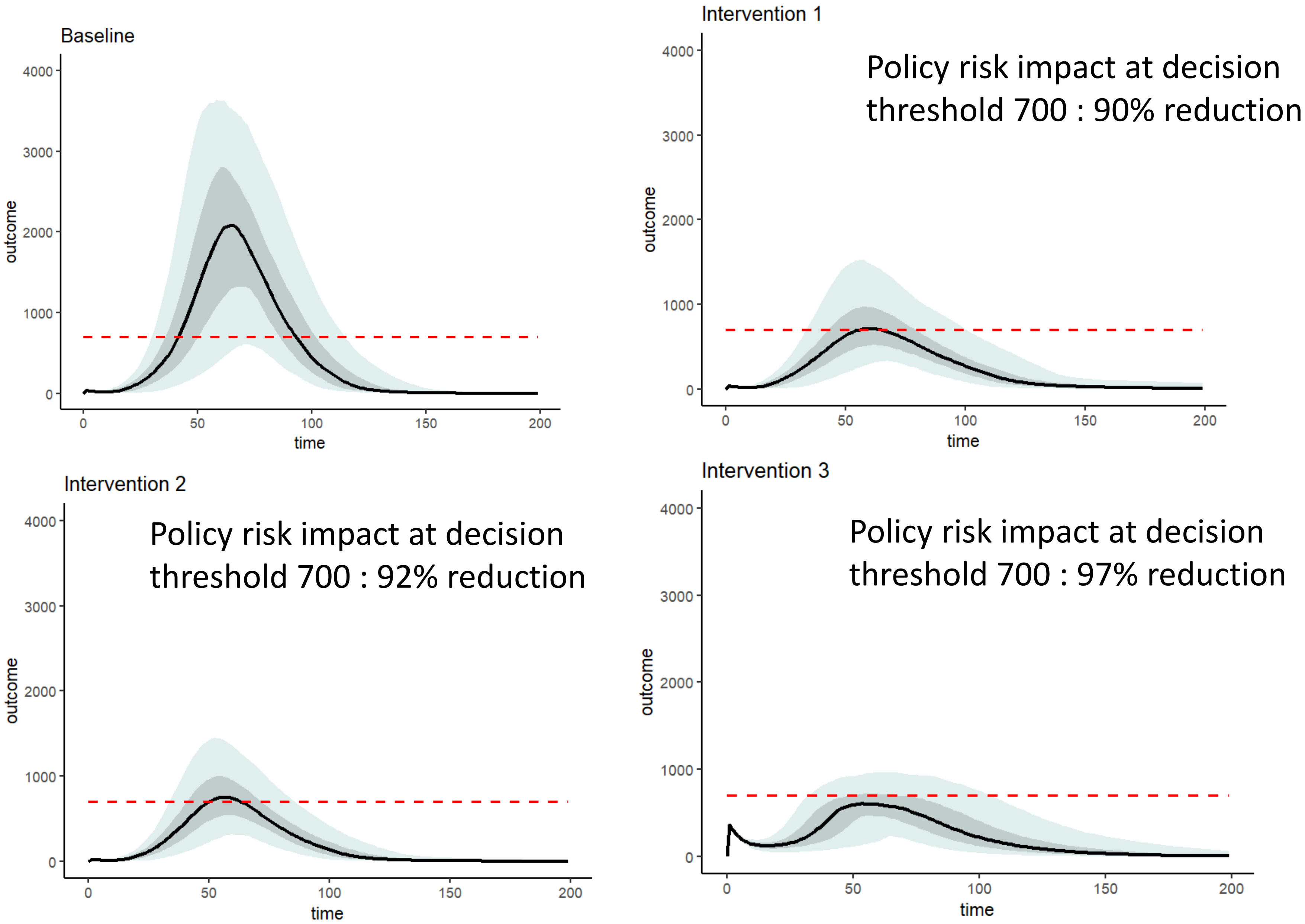

INSTITUTE OF HEALTH ECONOMICS
ALBERTA CANADA

Discussion: Toolkit content (15 min)

- Any thoughts, questions, or clarifications for the risk measure?
- Do you see using the weights feature to help adjust for expected risk?
- In terms of graphs, can you describe which types would you use when communicating uncertainty? E.g., fan plots, density plots, and raincloud plots (including temporal type)?
- In which order would you present the risk measure and graphs to best communicate uncertainty?

Discussion: Toolkit Coding (15 min)

- Are there thoughts, questions, or clarifications about the coding for the toolkit?
- How did you find using the code? How long did it take to learn how it works?
- Did you get errors? If so, what types of errors?
- How was it using the sample data? Was that communicated clearly in the readme file?
- Is there anything we can add or improve in the readme file?

Discussion: Trying out the Toolkit (35 min)

- Presentations (10-15 min each)

Discussion: Toolkit Standard Descriptions (10 min)

Decision Uncertainty Toolkit: Expected Risk

	Baseline	Intervention 1	Intervention 2	Intervention 3
Expected risk	47,661	4,777	4,035	1,374
Policy risk impact	-	-90%	-92%	-97%

Standard description for decision makers:

The expected risk values in the first row of the table above captures the probability of the intervention ***surpassing*** the specified policy target (i.e., how likely it is), the magnitude of the ***exceedance*** from target, and the length of time the ***exceedance*** is likely to last. Higher risk values indicate a greater risk that the intervention will not achieve the policy objective.

Interpretation of the risk value is more intuitive using a relative comparator. The policy risk impact in the second row of the table compares the risk associated with each intervention to the baseline scenario. The policy risk impact is interpreted as the percent change in risk relative to the baseline scenario. For example, the expected risk of ***exceeding*** the policy target in Intervention 1 is reduced by 90.0% relative to the baseline scenario.

Thoughts?

Decision Uncertainty Toolkit: Fan Plots

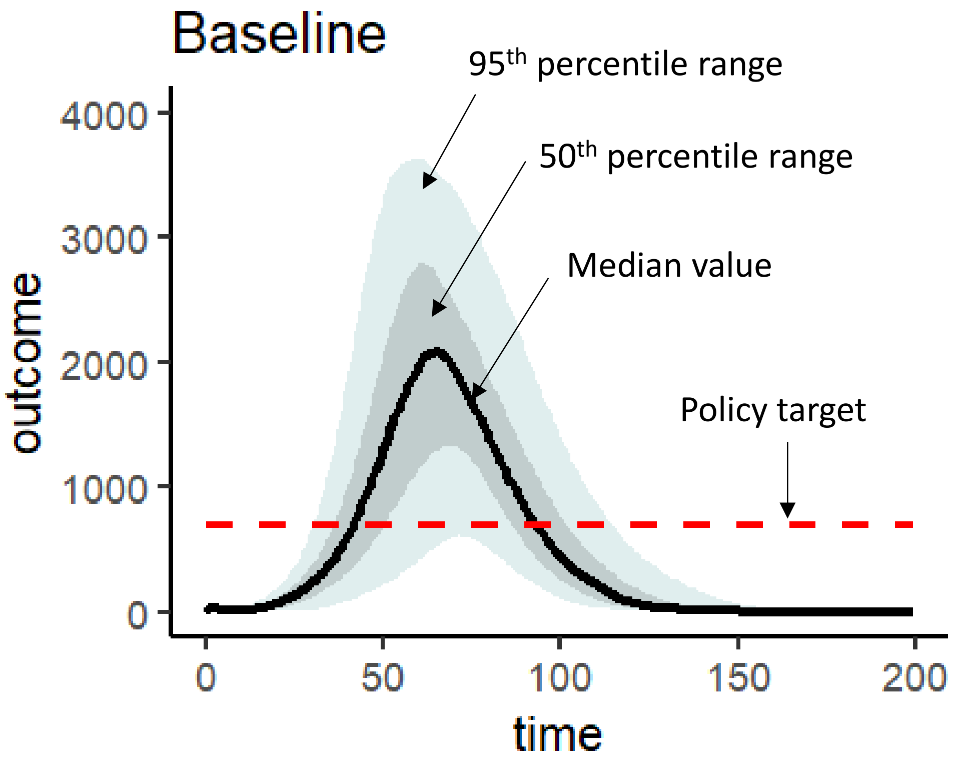

Plot elements:

- The red dashed line represents the policy target
- The solid black line shows the median outcome value
- The dark shaded area covers the 25 to 75 percentile range (where 50% of the outcome values fall)
- The lighter shade covers the 2.5 to 97.5 percentile range (where 95% of the outcome values fall)

Standard description for decision makers:

This graph visually displays the uncertainty surrounding the intervention's probability of surpassing the specified policy target. It indicates not only the degree of uncertainty but also provides insight into the magnitude by which the intervention is likely to ***exceed*** the target (through the use of percentile shading) and the anticipated duration of the ***exceedance***.

The magnitude and the length of time that the shaded areas ***extend beyond*** the policy target (dashed red line) signify the risk that the intervention will not achieve the policy objective. More shaded area ***above*** the dashed red line for longer periods indicates a higher risk of not achieving the policy objective.

Do you like the annotations on the chart itself and the text description here or maybe just one of the two?

INSTITUTE OF HEALTH ECONOMICS
ALBERTA CANADA

Decision Uncertainty Toolkit: Risk shading

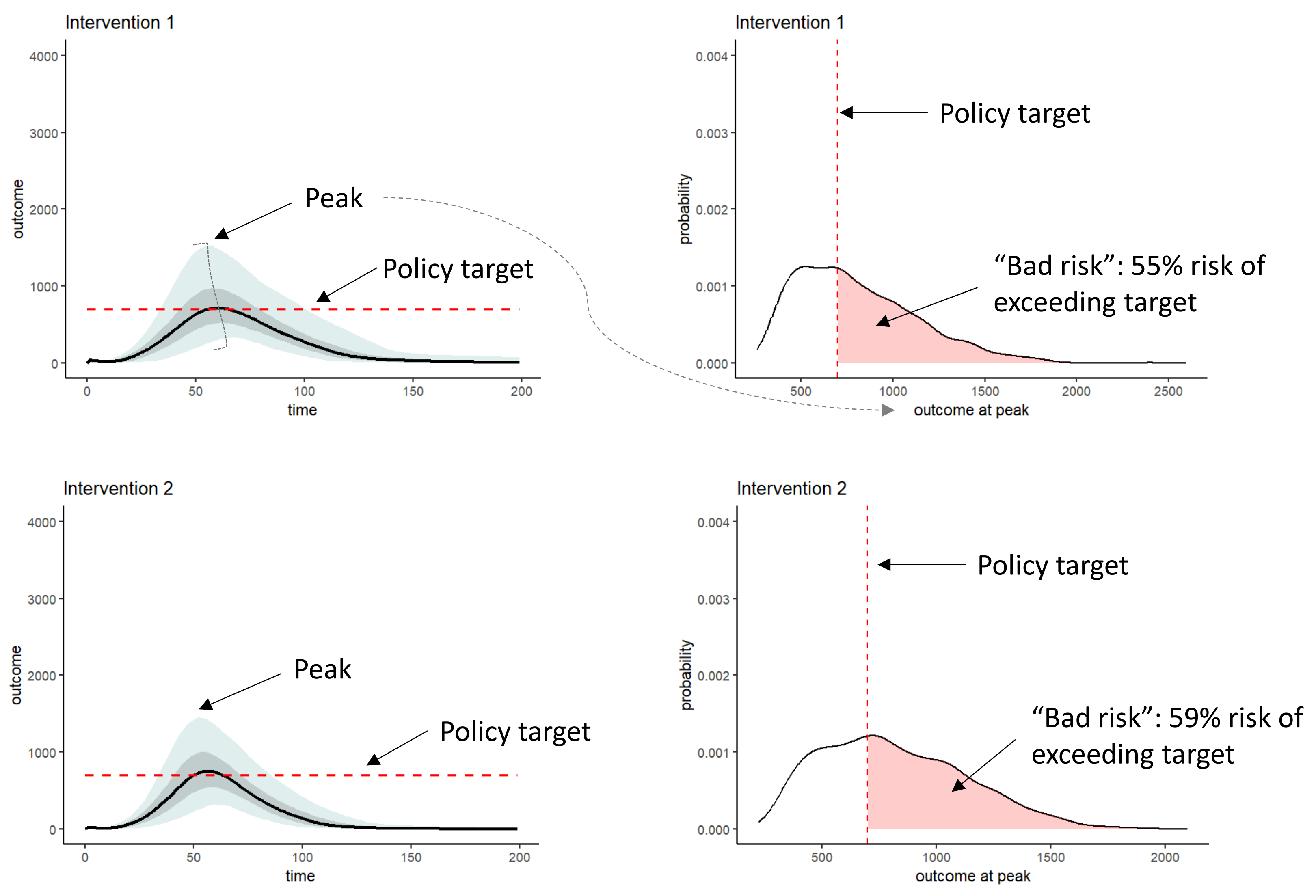

Standard description for decision makers:
These density graphs show the distribution of the ***highest*** forecasted *outcome** (i.e., ***the peak***). The red dashed line indicates the policy target. The shaded area indicates how likely it is that the *outcome** at its forecasted ***peak*** will ***surpass*** the policy target, or simply, the amount of "bad risk." A larger shaded area means more "bad risk."

**insert outcome description (ex. hospital census, # of total infections)*

Trying to illustrate density graph is a distribution at the peak

What do you think of using the dotted line?

INSTITUTE OF HEALTH ECONOMICS
ALBERTA CANADA

Decision Uncertainty Toolkit: Temporal plots

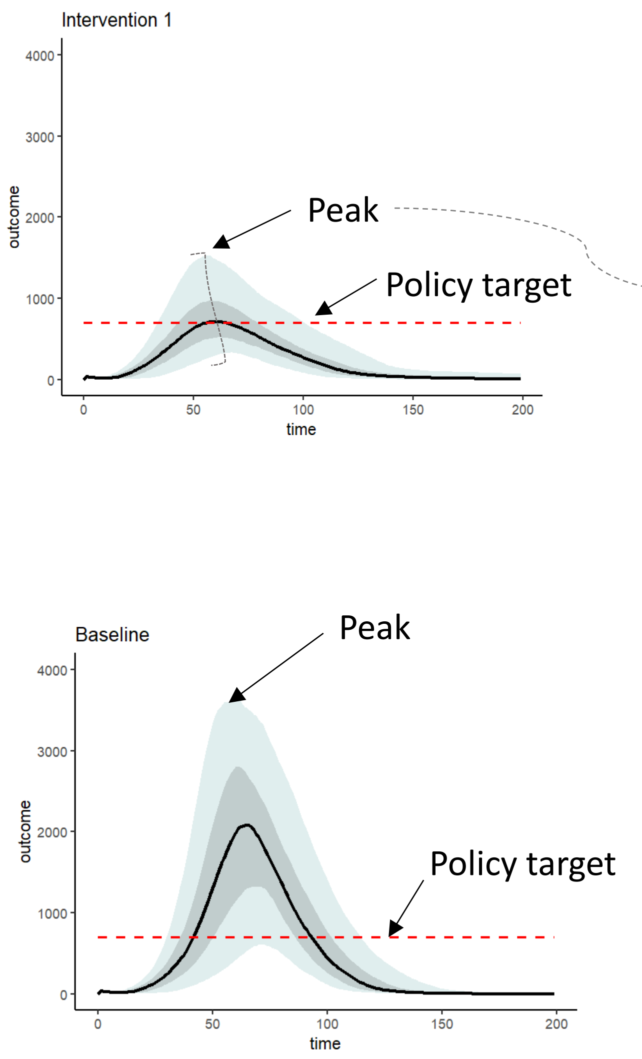

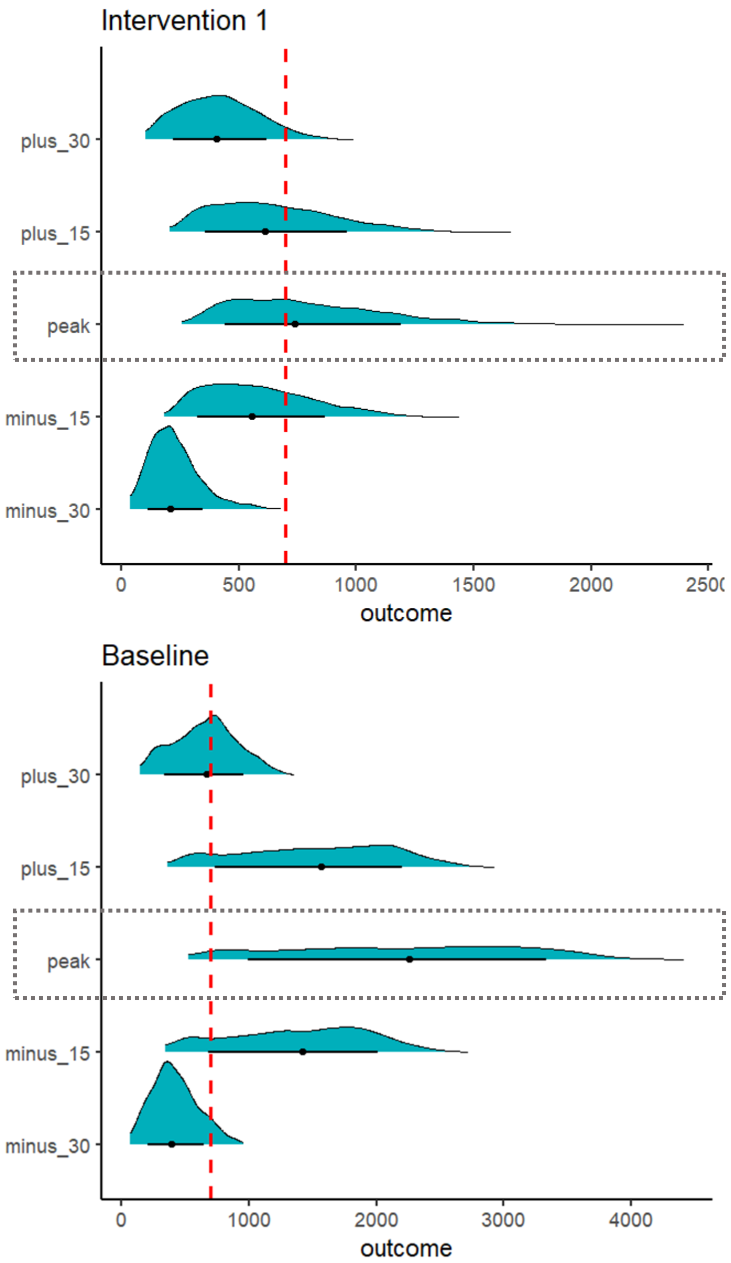

Standard description for decision makers:
This graph visually illustrates how uncertainty changes over time by showing the distributions of the forecasted *outcome** over time. The distribution in the center of the plot represents the ***peak*** *outcome**. The distributions above and below show the *outcome** at different time points relative to the ***peak***, both forward (above) and backward (below) in time.

The red dashed line indicates the policy target. A greater area to the ***right*** of this line means a higher chance of the *outcome** ***exceeding*** the policy target at the specified time. Scenarios where the area to the ***right*** of the policy target is quickly reduced as you move away from the ***peak*** result in a shorter timeframe of higher uncertainty.

**insert outcome description (ex. hospital census, # of total infections)*

INSTITUTE OF HEALTH ECONOMICS
ALBERTA CANADA

Discussion: Toolkit Standard Descriptions

- Do you feel you could explain the risk measure and graphs to others with the help of the standard descriptions?

Thank-you

mvarughese@ihe.ca
mwiggins@ihe.ca
noak@ihe.ca

The Decision Uncertainty Toolkit – Decision Maker Workshop

March 22, 2024

Institute of Health Economics

Megan Wiggins, Marie Betsy Varughese, Ellen Rafferty, Jeff Round, Sasha van Katwyk, Erin Kirwin

Thank you to Nicole Oak for helping us organize this workshop!

INSTITUTE OF HEALTH ECONOMICS
ALBERTA CANADA

Funding Acknowledgements

- Canadian Network for Modelling Infectious Diseases (CANMOD)
- One Society Network (NSERC)

CANMOD

Canadian Network for Modelling Infectious Diseases

Réseau canadien de modélisation des maladies infectieuses

Strengthening the community of people who are making and using infectious disease models to improve public health both inside and outside of Canada

One Society Network

Interdisciplinary Pandemic Impact Modelling

Agenda

Time	Agenda Item	Lead
8:30 – 8:40 AM MT	Welcome & Introductions	Betsy
8:40 – 8:50	Icebreaker Activity	
8:50 – 9:10	Overview of the toolkit	
9:10 – 9:30	Toolkit content (i.e., risk measures and graphical components)	
9:30 – 9:45	Visualization and Risk Measure	
9:45 – 10:00	Standard Descriptions	
10:00 – 10:15	Communication of model uncertainty	
10:15 – 10:30	Wrap-up & Questions	

Introductions & Ice-Breaker Activity (10 min)

- Your name and role?
- What are your areas of expertise/interest/research background?
- What is your favorite food and why?

Overview of the Toolkit (20 min)

- The Aim: To characterize, visualize, and communicate decision risk for infectious disease models

- 'Decision Uncertainty Toolkit' developed through engagement with infectious disease modelers and decision makers
  - Visualization tools
  - New measures of risk
  - R bookdown document with standard description text and codes (living repository)

Working Draft Shared on GitHub

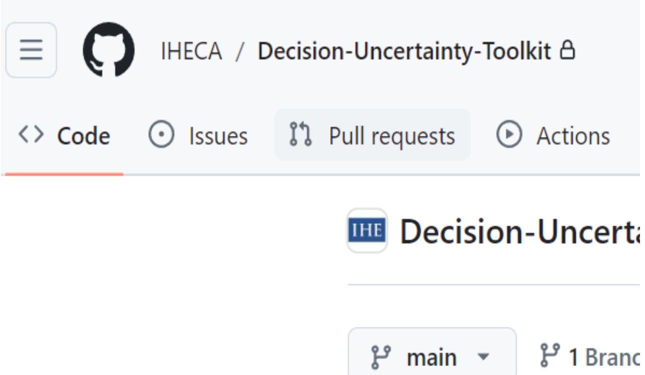

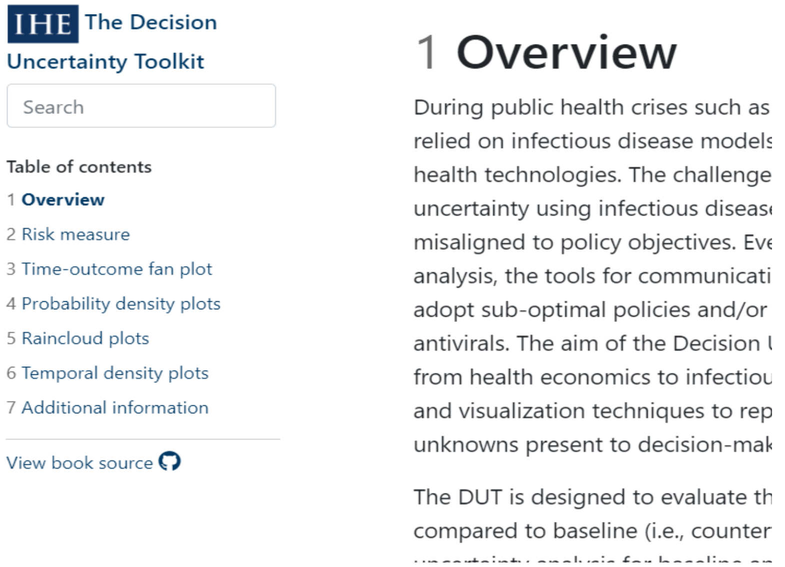

INSTITUTE OF HEALTH ECONOMICS
ALBERTA CANADA

Infectious Disease Models

- Statistical & mathematical methods
- Models that capture disease dynamics generally use mathematical modelling
  - Understand disease dynamics e.g., transmission, vaccine, in-host, vector-borne, zoonotic etc.
  - Assess impact of interventions on a simulated population of people e.g., hospitalizations, disease burden, and death
  - Estimate model parameters that cannot be estimated from data

Deterministic Mathematical Model

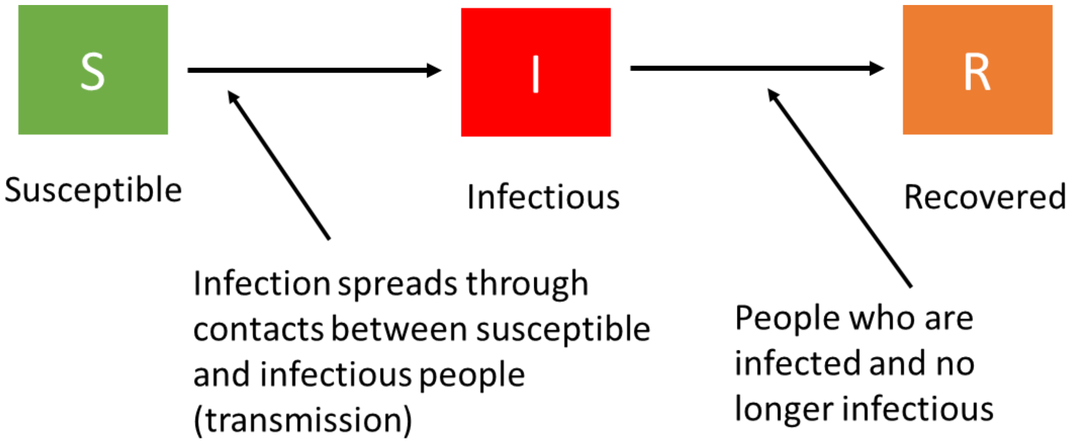

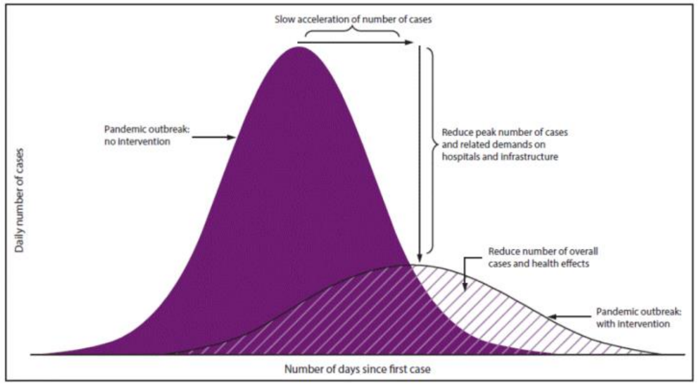

Developed by the CDC, this graphic has been used frequently to explain COVID-19 prevention and response.

Models were an important part of decision making during the COVID-19 response internationally

- The explicit use models for decision making on such a large scale.

- Huge opportunity for evidence-informed decision making.

- Not all decision makers were prepared to interface with modelers, and vice-versa.

- When there are gaps in the communication of model assumptions and uncertainty, the results of models are difficult to interpret for decision makers.

Why are we doing this? The Problem

- Means do not provide information about outcome uncertainty and therefore risk.
- Important for skewed distributions such as cases, hospitalizations, or deaths.
- Essentially, decision makers can be unaware of the risk associated with alternative policy options, and/or without tools to consider this risk.

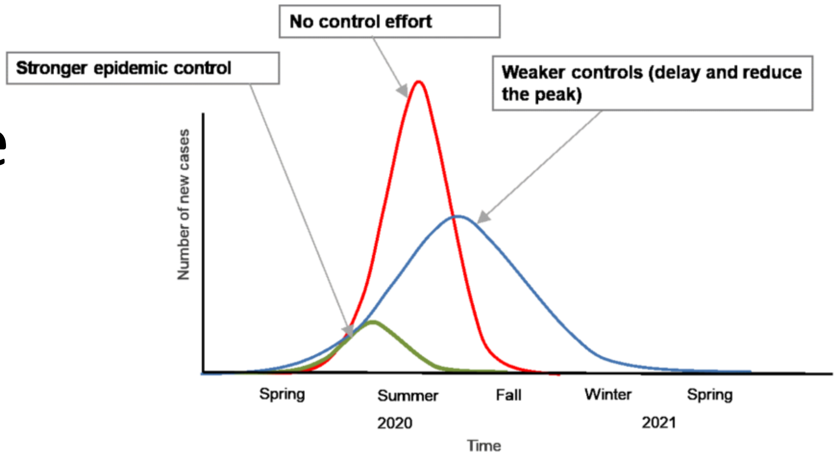

https://www.canada.ca/en/public-health/services/publications/diseases-conditions/covid-19-using-data-modelling-inform-public-health-action-april-28-2020.html

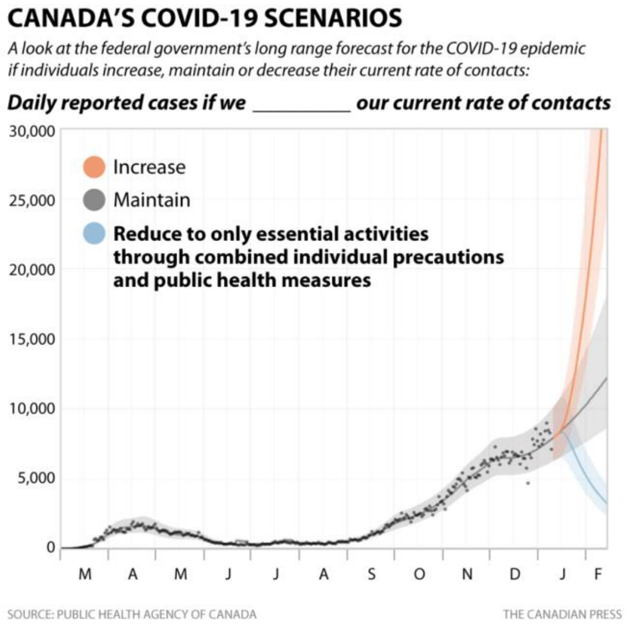

https://www.cbc.ca/news/politics/phac-modelling-covid19-1.5874530

What is Modeling Uncertainty?

- Structural: e.g., Susceptible – Infectious – Recovered OR Susceptible – Exposed – Infectious – Recovered?
- Parameters: Often these values include a 95% CI e.g., length of stay: 15 days 95% (6-18 days)
- Decision: What decision do we make if we see multiple policy options? Do we look at the mean or account for the other possible simulations? How do we then compare across policy options?

Decision Uncertainty Toolkit

https://bmjopen.bmj.com/content/11/9/e047227

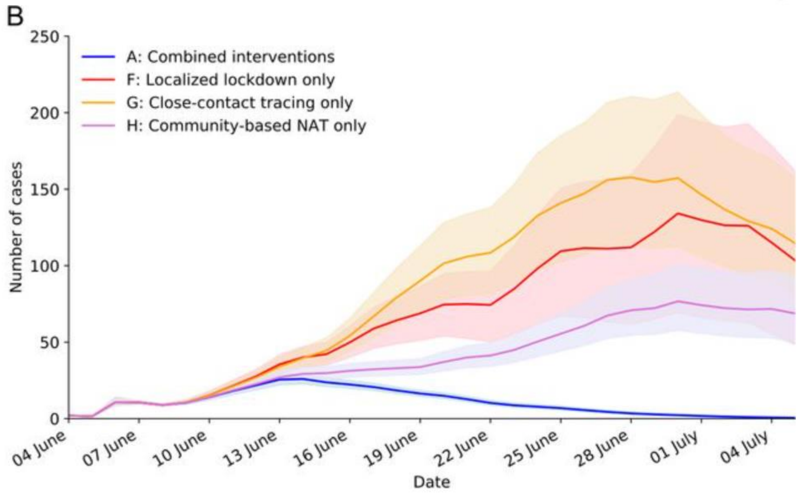

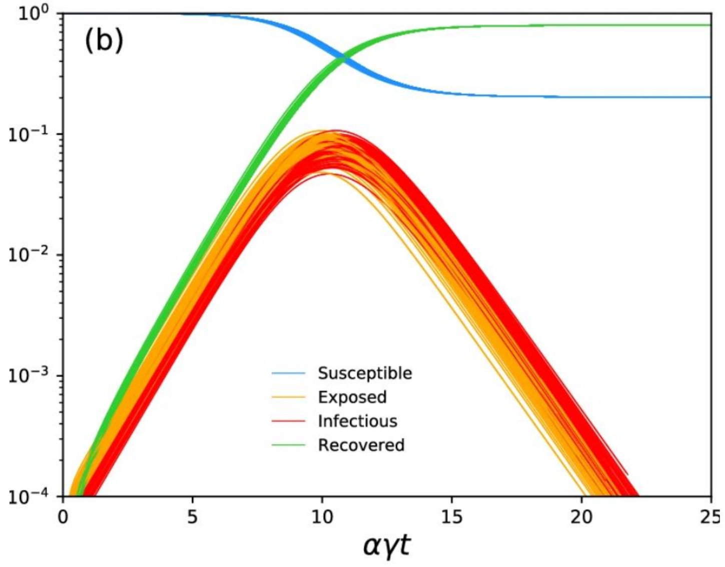

by $\alpha\gamma$. For illustration, the basic reproduction number has been set to
o $I_0 = 10^{-4}$. Each set of curves is generated using 100 random realisations
and 5 days for illustration.

https://www.nature.com/articles/s41598-020-76563-8/figures/1

Discussion Questions

- What has been your experience in communicating policy options to decision makers? Has there been challenges?

- What is your understanding of modelling uncertainty?

- What has been your experience communicating modeling results that include uncertainty to decision makers? Has there been challenges?
  - Was there questions around how to present 95% CI and/or decisions around policy?

- From your perspective, how do decision makers understand model uncertainty when interpreting results?

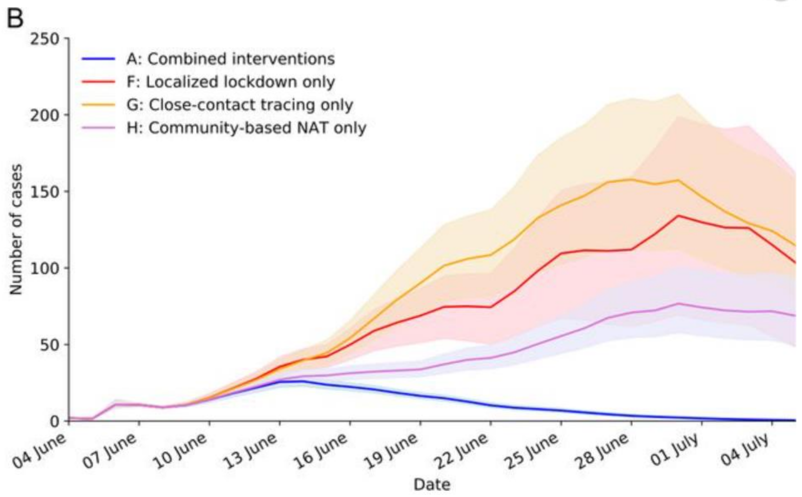

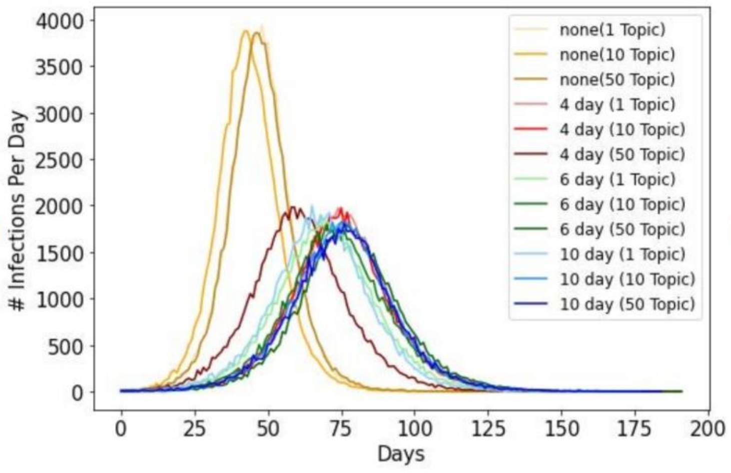

Disease Spread Simulation Results after Prescribing Interventions 7.3 Effect of Policy Interventions A variety of experiments are implemented to test the impact of various public health interventions on the spread of COVID-19 and the subsequent effect on the epidemic curve.

Fig. 3

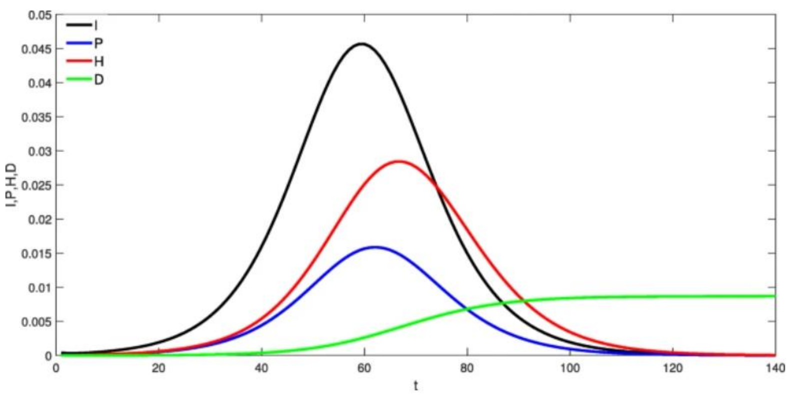

The epidemic dynamics of the model (Eq. (1)) with no interventions. The symptomatic infectious (I, black curve), severe infections (P, blue curve), hospitalized infectious (H, red curve) and dead (D, green curve) are shown in time

Decision Uncertainty Toolkit Components (25 min)

Decision Uncertainty Toolkit

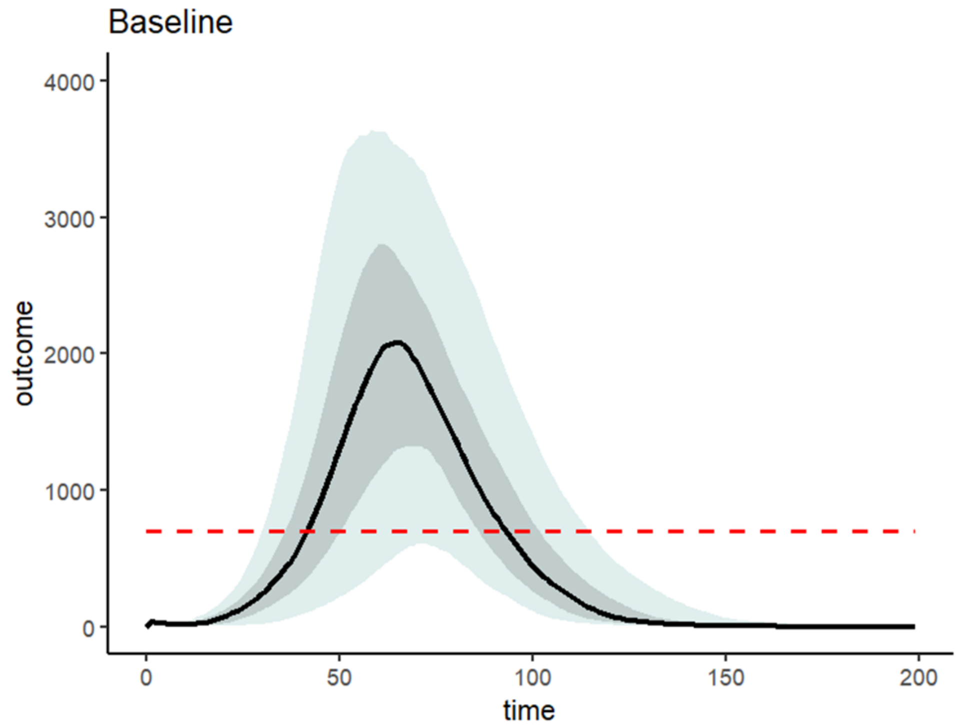

- Visualizations
- Risk Measure
- Descriptions (or Interpretations)

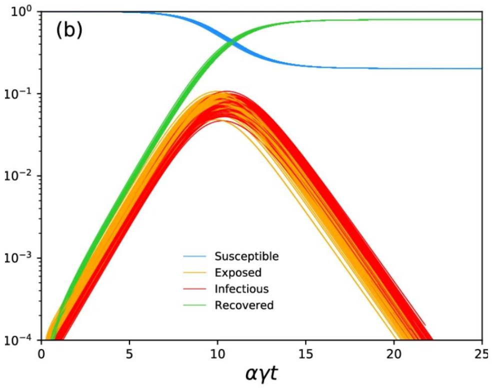

ɔy $\alpha\gamma$. For illustration, the basic reproduction number has been set to
o $I_0 = 10^{-4}$. Each set of curves is generated using 100 random realisations
and 5 days for illustration.

Risk measure formula

For each scenario the expected risk measure is calculated as follows:

$$Expected\ Risk = \begin{cases} \dfrac{\sum_{n=1}^{N} W_n \times \sum_{t=t_{min}}^{t_{max}} (\max(D_t, O_{nt}) - D_t)}{N} & if\ D_t\ is\ a\ maximum \\ \dfrac{\sum_{n=1}^{N} W_n \times \sum_{t=t_{min}}^{t_{max}} (D_t - \min(D_t, O_{nt}))}{N} & if\ D_t\ is\ a\ minimum \end{cases}$$

INSTITUTE OF HEALTH ECONOMICS ALBERTA CANADA

Part 1: Visualizations

Example output:

Fan plot

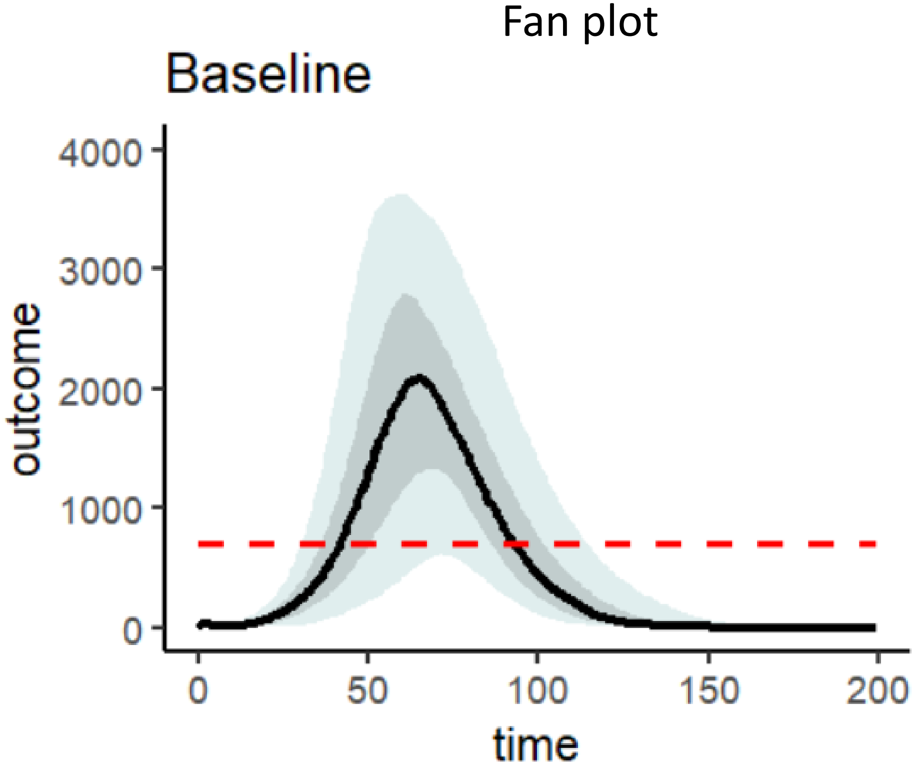

Example output:

Probability density plot

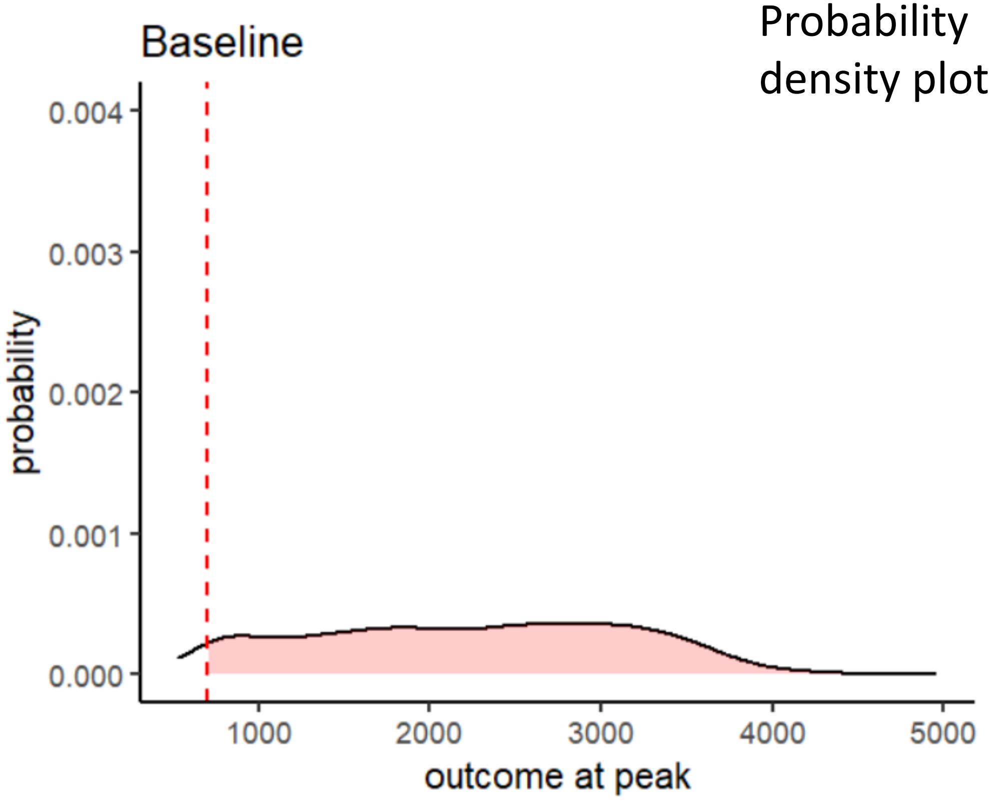

Part 2: Risk Measures I

Risk measure formula

For each scenario the expected risk measure is calculated as follows:

$$Expected\ Risk = \begin{cases} \dfrac{\sum_{n=1}^{N} W_n \times \sum_{t=t_{min}}^{t_{max}} (\max(D_t, O_{nt}) - D_t)}{N} & if\ D_t\ is\ a\ maximum \\ \dfrac{\sum_{n=1}^{N} W_n \times \sum_{t=t_{min}}^{t_{max}} (D_t - \min(D_t, O_{nt}))}{N} & if\ D_t\ is\ a\ minimum \end{cases}$$

Where:

- *n = 1…N* is the number of simulation runs
- $t = t_{min} \ldots t_{max}$ is the simulation time
- O_{nt} is the observed outcome for simulation run *n* at time *t*
- D_t is the decision threshold at time *t*
- W_n is the weight assigned to simulation run *n*

Note: *If Dt is a maximum decision threshold, the expected risk indicates the risk of* ***exceeding*** *the threshold value; If Dt is a minimum decision threshold, the expected risk indicated the risk of* ***falling under*** *the threshold value.*

- Work with decision makers to define policy thresholds that can be used to measure risk **across scenarios**
- Quantify distance from threshold and probability of exceeding the threshold (i.e., the first formula)
- Risk evaluated for a specified time interval

Part 2: Risk Measures II

Risk measure formula

For each scenario the expected risk measure is calculated as follows:

$$Expected\ Risk = \begin{cases} \frac{\sum_{n=1}^{N} W_n \times \sum_{t=t_{min}}^{t_{max}} (\max(D_t, O_{nt}) - D_t)}{N} & if\ D_t\ is\ a\ maximum \\ \frac{\sum_{n=1}^{N} W_n \times \sum_{t=t_{min}}^{t_{max}} (D_t - \min(D_t, O_{nt}))}{N} & if\ D_t\ is\ a\ minimum \end{cases}$$

Where:

- *n = 1…N* is the number of simulation runs
- $t = t_{min} \ldots t_{max}$ is the simulation time
- O_{nt} is the observed outcome for simulation run *n* at time *t*
- D_t is the decision threshold at time *t*
- W_n is the weight assigned to simulation run *n*

Note: *If Dt is a maximum decision threshold, the expected risk indicates the risk of* ***exceeding*** *the threshold value; If Dt is a minimum decision threshold, the expected risk indicated the risk of* ***falling under*** *the threshold value.*

- Interpretation of risk value is easier with a relative comparator (ex. what does a value of 370 mean?)
  - Define a 'baseline' comparator and calculate relative values
  - Ex. baseline risk is 500, scenario risk is 370
  - (370-500)/500 = -0.26, **so risk is reduced by 26%**

INSTITUTE OF HEALTH ECONOMICS
ALBERTA CANADA

Part 3: Descriptions

Descriptions of toolkit elements

- Descriptions of toolkit elements
- Descriptions of approaches to uncertainty
  - Standard descriptions and examples
  - Aim to use common or example language that has been supported by decision makers and modelers through engagement

From GitHub:

Standard description text for risk table

We recommend the following standard description for presenting the risk table above to decision makers:

The expected risk values in the first row of the table above captures the probability of the intervention surpassing the specified policy target (i.e., how likely it is), the magnitude of the exceedance from target, and the length of time the exceedance is likely to last. Higher risk values indicate a greater risk that the intervention will not meet the policy objective.

Interpretation of the risk value is more intuitive using a relative comparator. The policy risk impact in the second row of the table compares the risk associated with each intervention to the baseline scenario. The policy risk impact is interpreted as the percent change in risk relative to the baseline scenario. For example, the expected risk of exceeding the policy target in Intervention 1 is reduced by 90.0% relative to the baseline scenario.

Example

Example: Decision Scenario

Decision maker is selecting between multiple policies:

A. Baseline – do nothing
B. Intervention 1 – e.g., close schools
C. Intervention 2 – e.g., mandatory masking
D. Intervention 3 – e.g., close schools + mandatory masking

Policy target: keep hospitalizations under 700 (capacity maximum)

Note: All these graphs are synthetic simulations and used for illustration purposes only

Decision Uncertainty Toolkit Example Plots

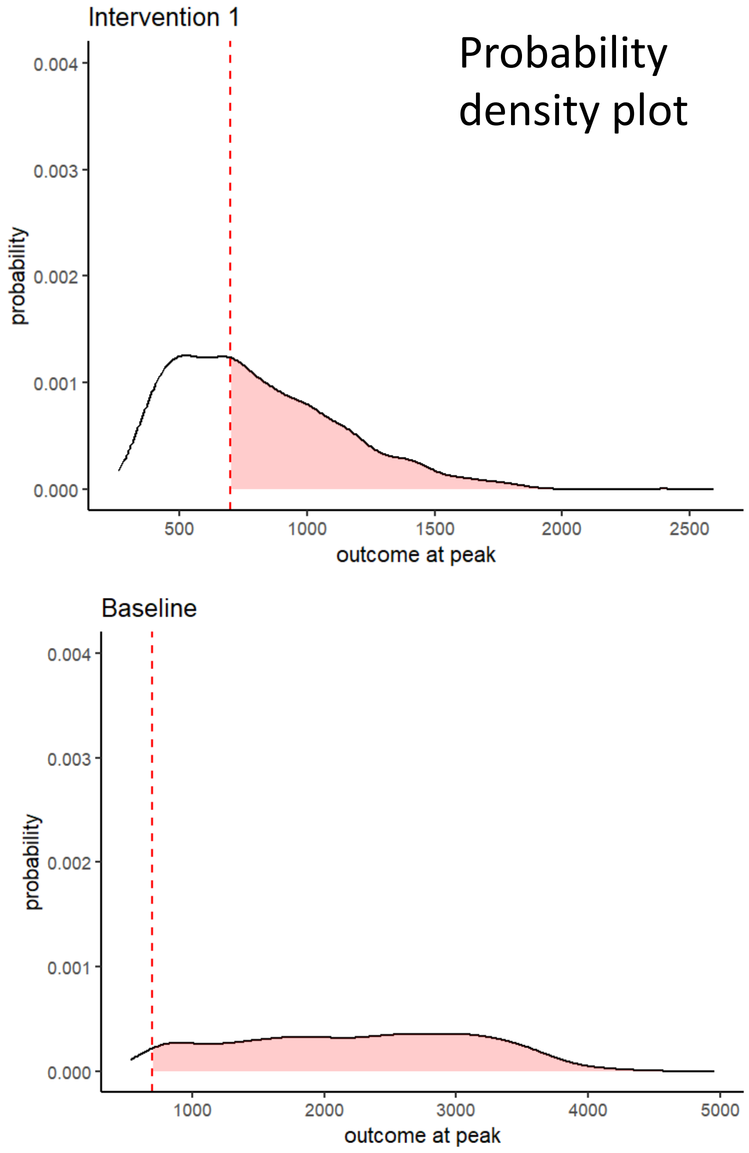

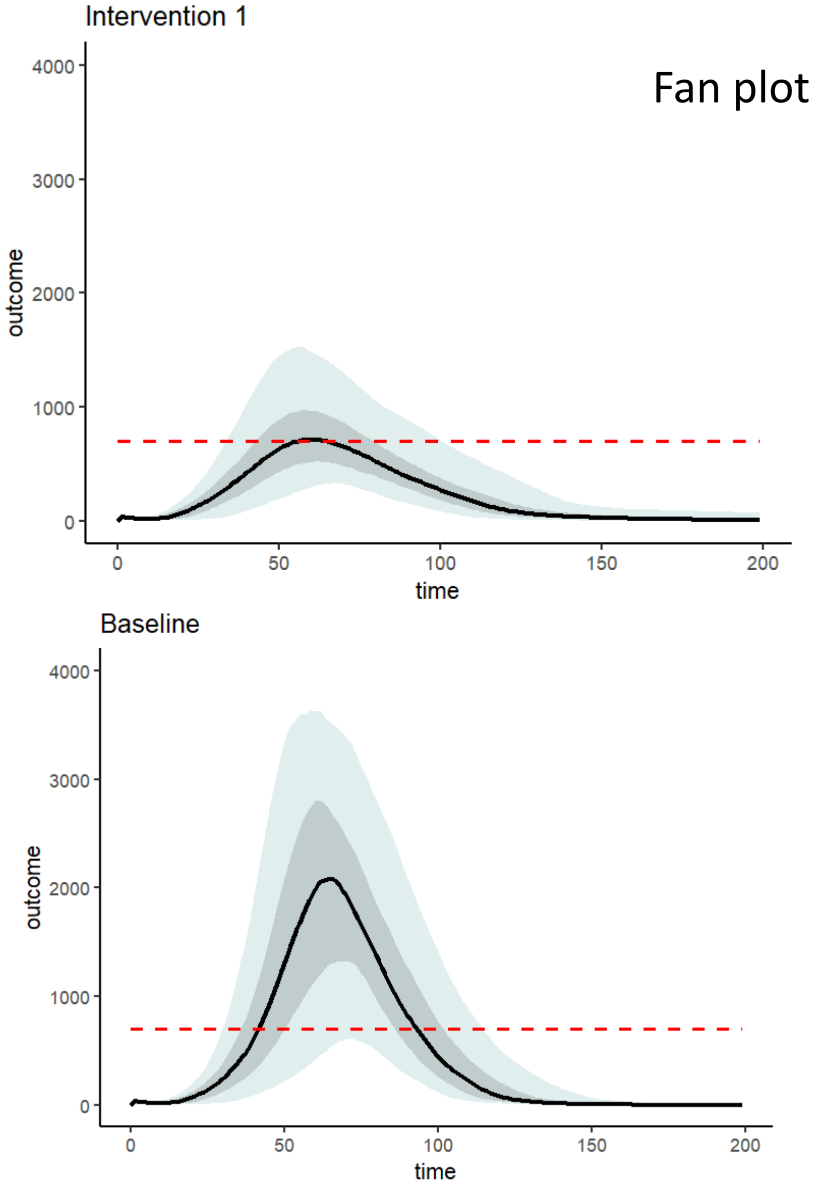

Decision Uncertainty Toolkit Example Plots

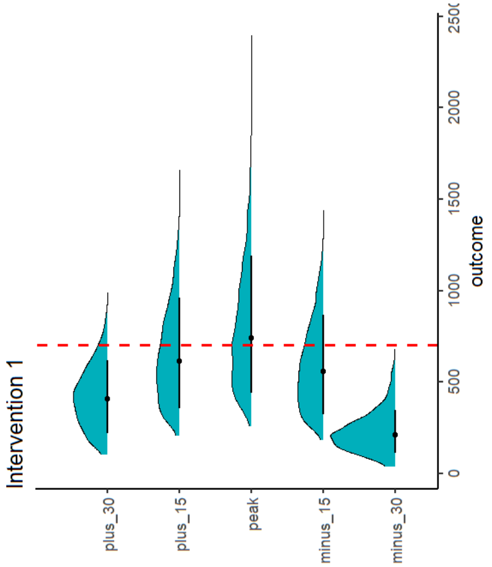

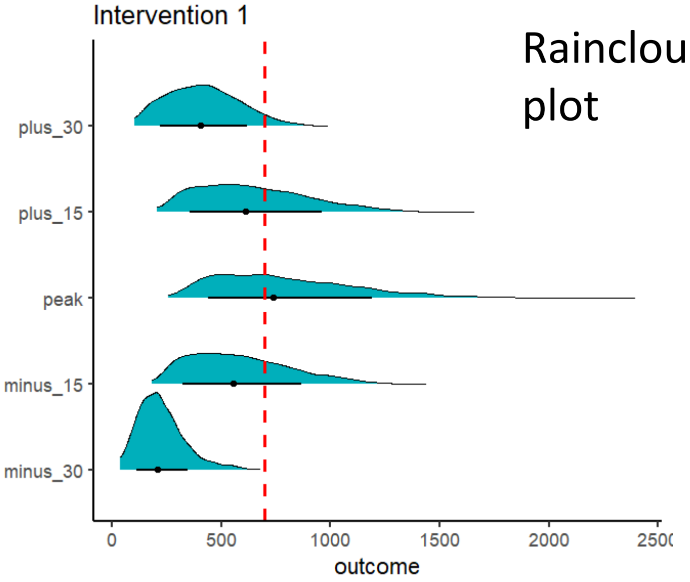

Raincloud plot

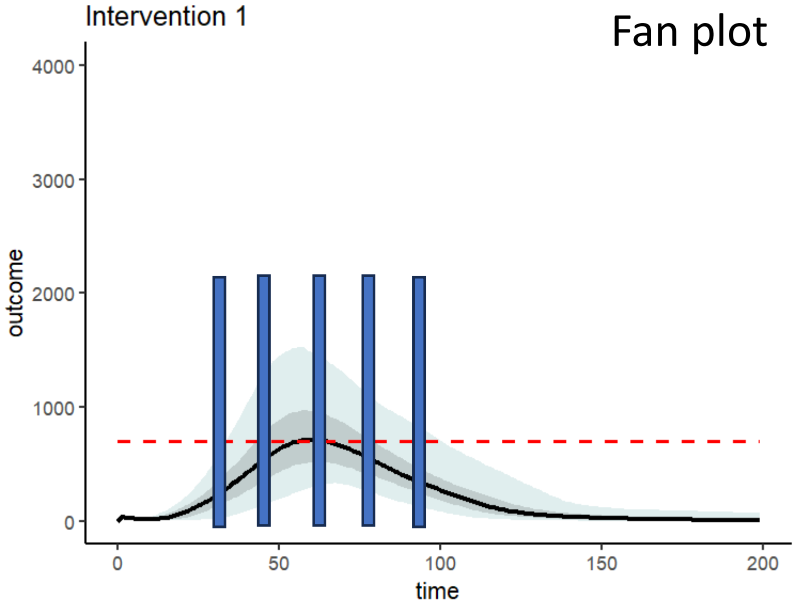

Fan plot

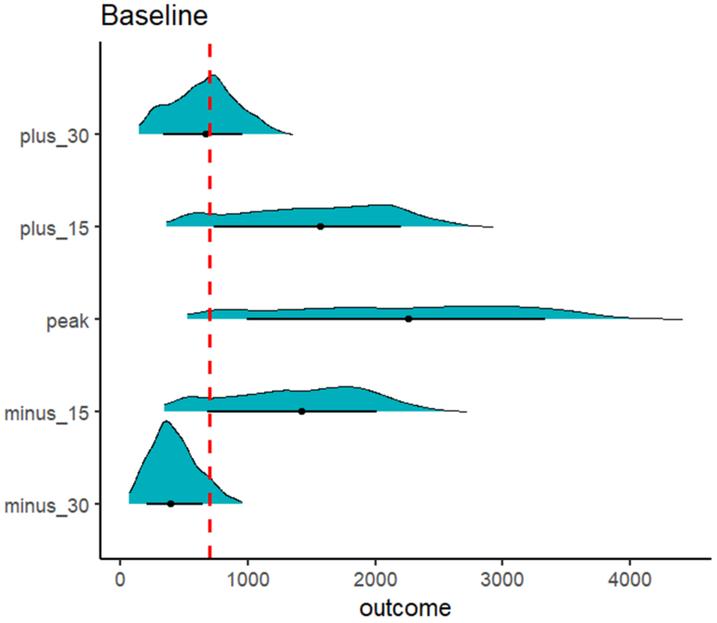

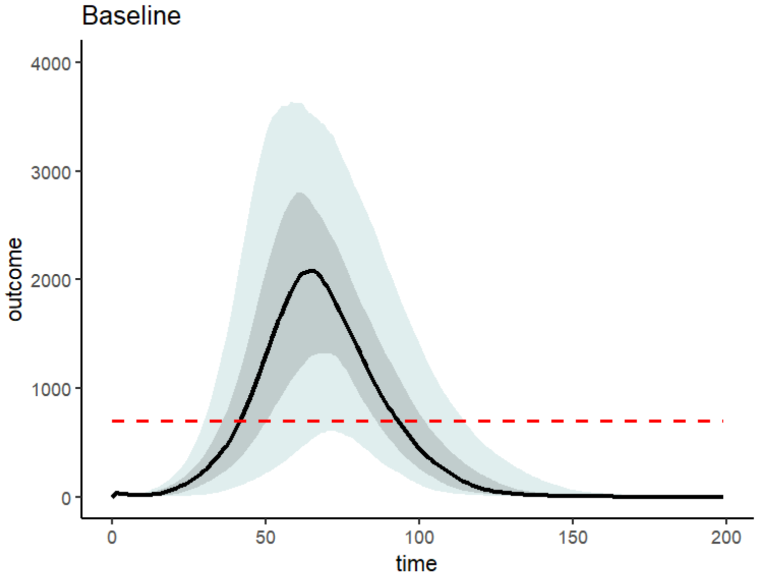

INSTITUTE OF HEALTH ECONOMICS
ALBERTA CANADA

Decision Uncertainty Toolkit: Expected Risk

	Baseline	Intervention 1	Intervention 2	Intervention 3
Expected risk	45,513	3,862	3,267	844
Policy risk impact	-	-91.5%	-92.8%	-98.1%

- Time range: 0 to 199 days
- Relative comparison to baseline model (no intervention)
- Interpretation of expected risk is relative to baseline
- e.g., for Intervention 1 - (3862 – 45,513)/45,513 = -91.5%

Risk tolerance

Decision Uncertainty Toolkit: Expected Risk

	Baseline	Intervention 1	Intervention 2	Intervention 3
Risk	45,513	3,862	3,267	844
Policy risk impact	-	-91.5%	-92.8%	-98.1%

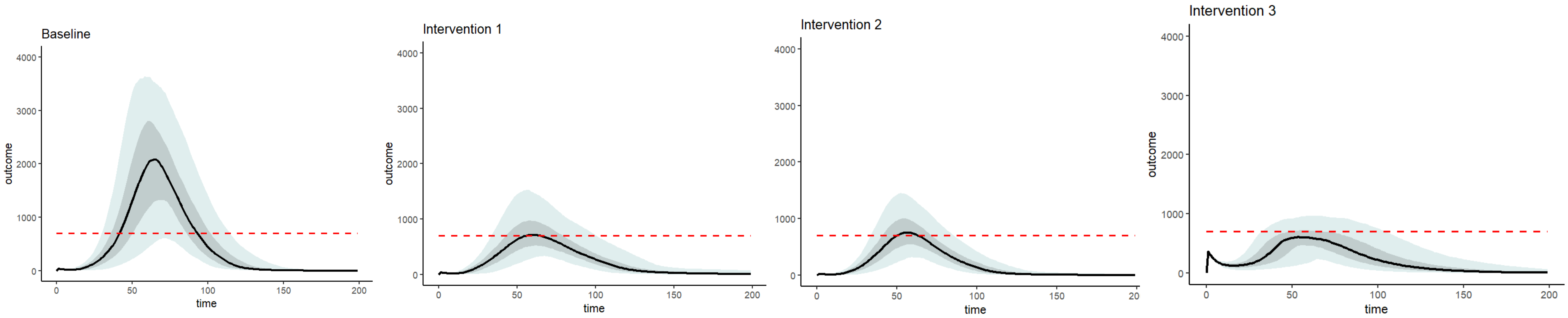

Discussion: Visualization and Risk Measure (15 min)

Fan Plot with Additional Information

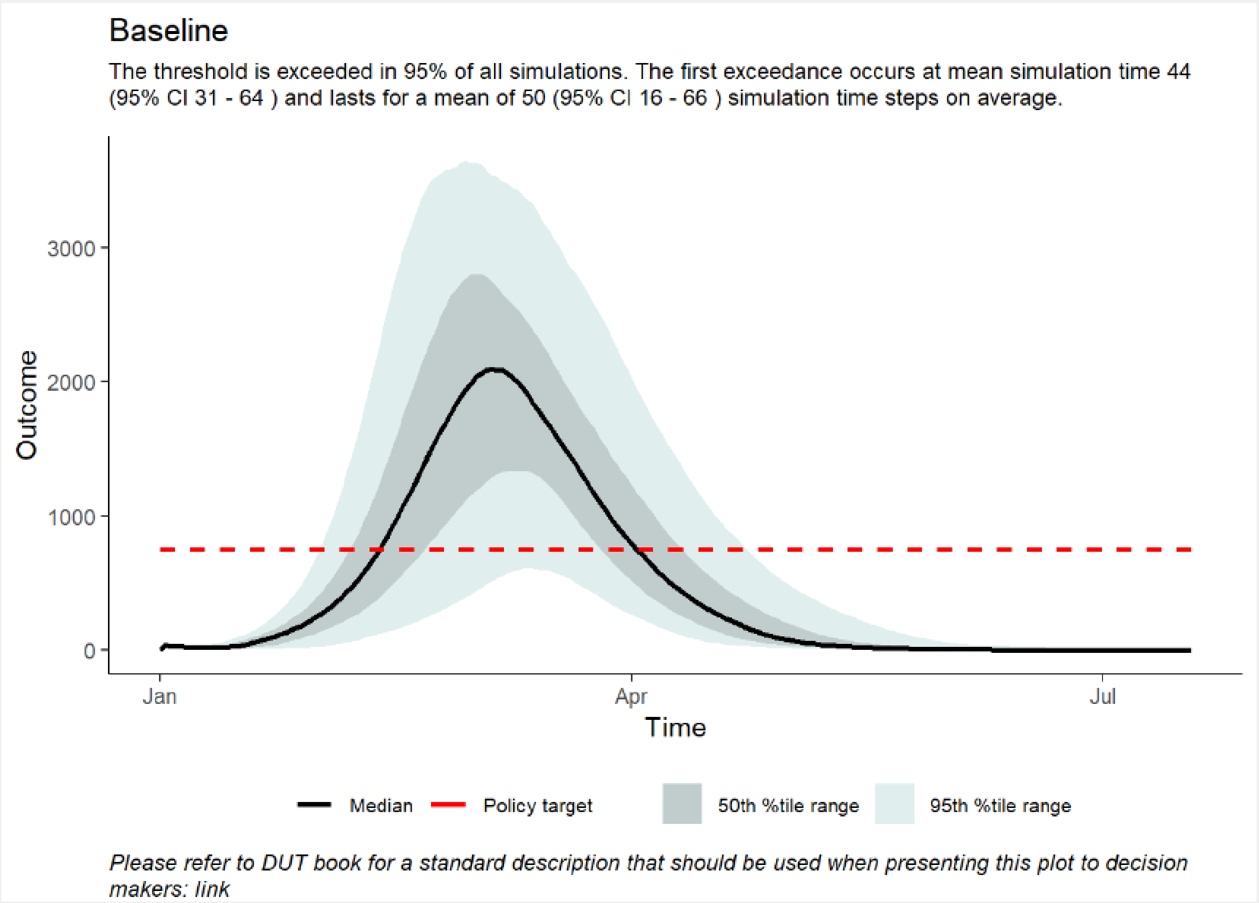

- What are your general thoughts about this figure i.e., could you show this type of result to a decision maker?
  - What do you like about this figure?
  - Anything we can improve on? Or add?

Fan Plot with Additional Information

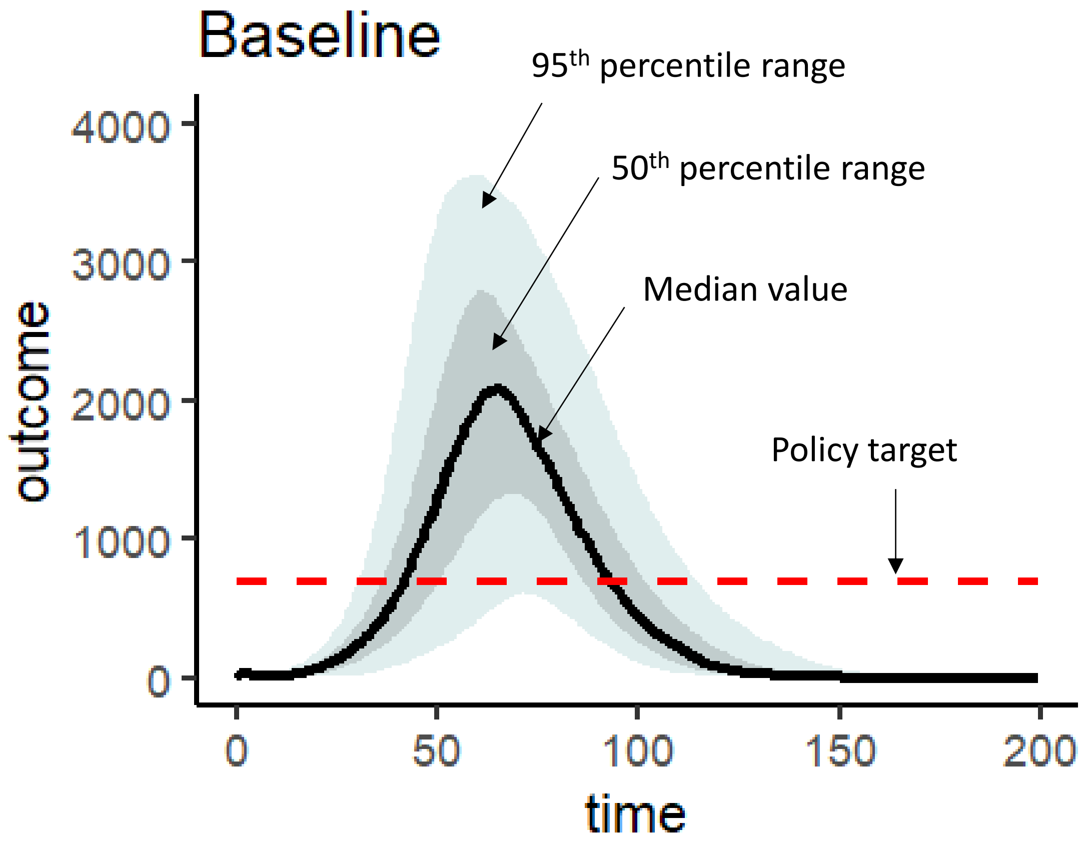

- What are your general thoughts about this figure in terms of annotating it this way?
  - What do you like about this figure?
  - Anything we can improve on? Or add?

Fan Plot with Policy Risk Measure

What are your general thoughts about these figures i.e., could you sho this type of result to a decision maker?

What do you like about this figure?

Anything we can improve on? Or add?

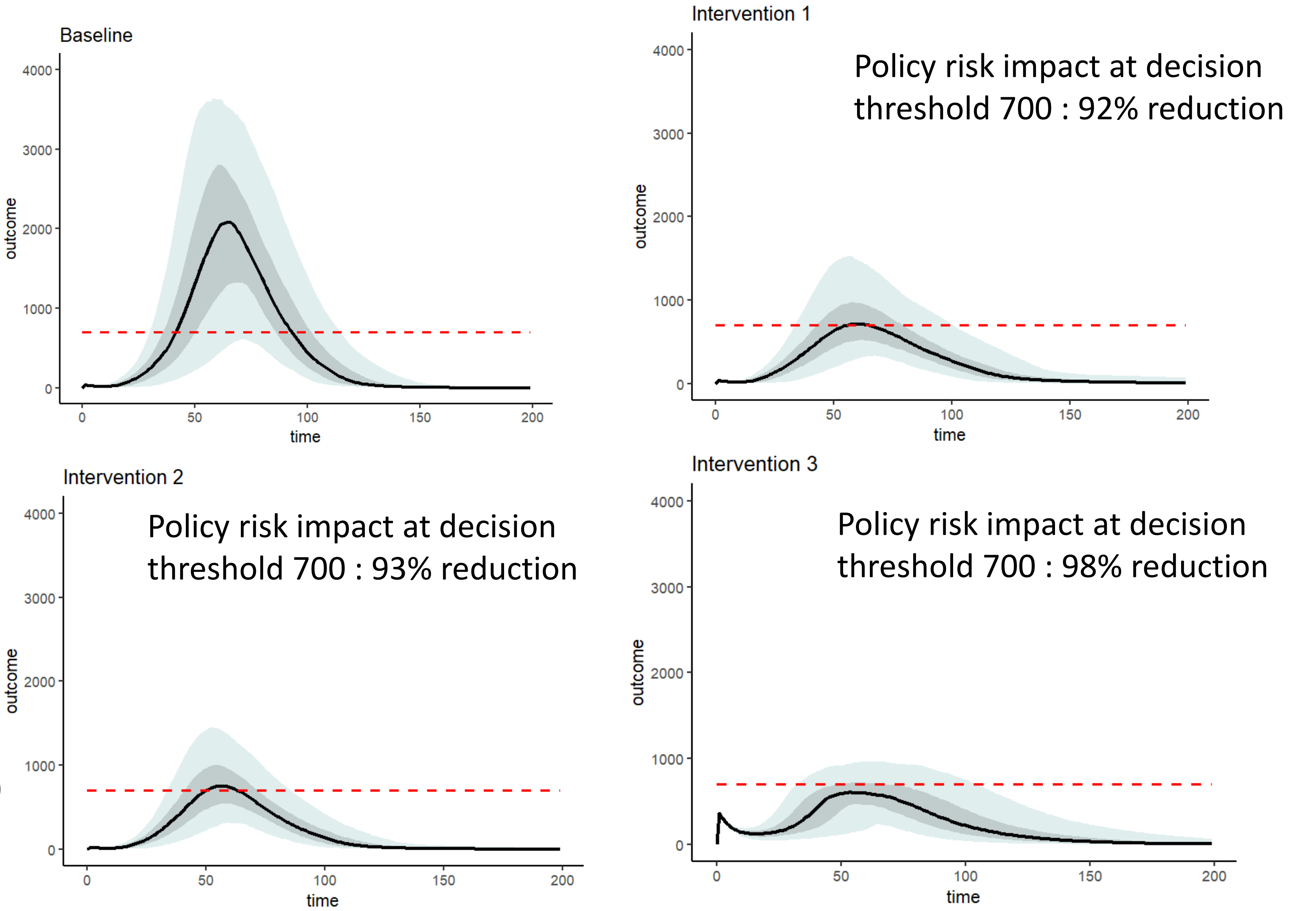

Decision Threshold = 700

Fan Plots with Additional Information

What are your general thoughts about these figures i.e., could you show this type of result to a decision maker?

What do you like about this figure?

Anything we can improve on? Or add?

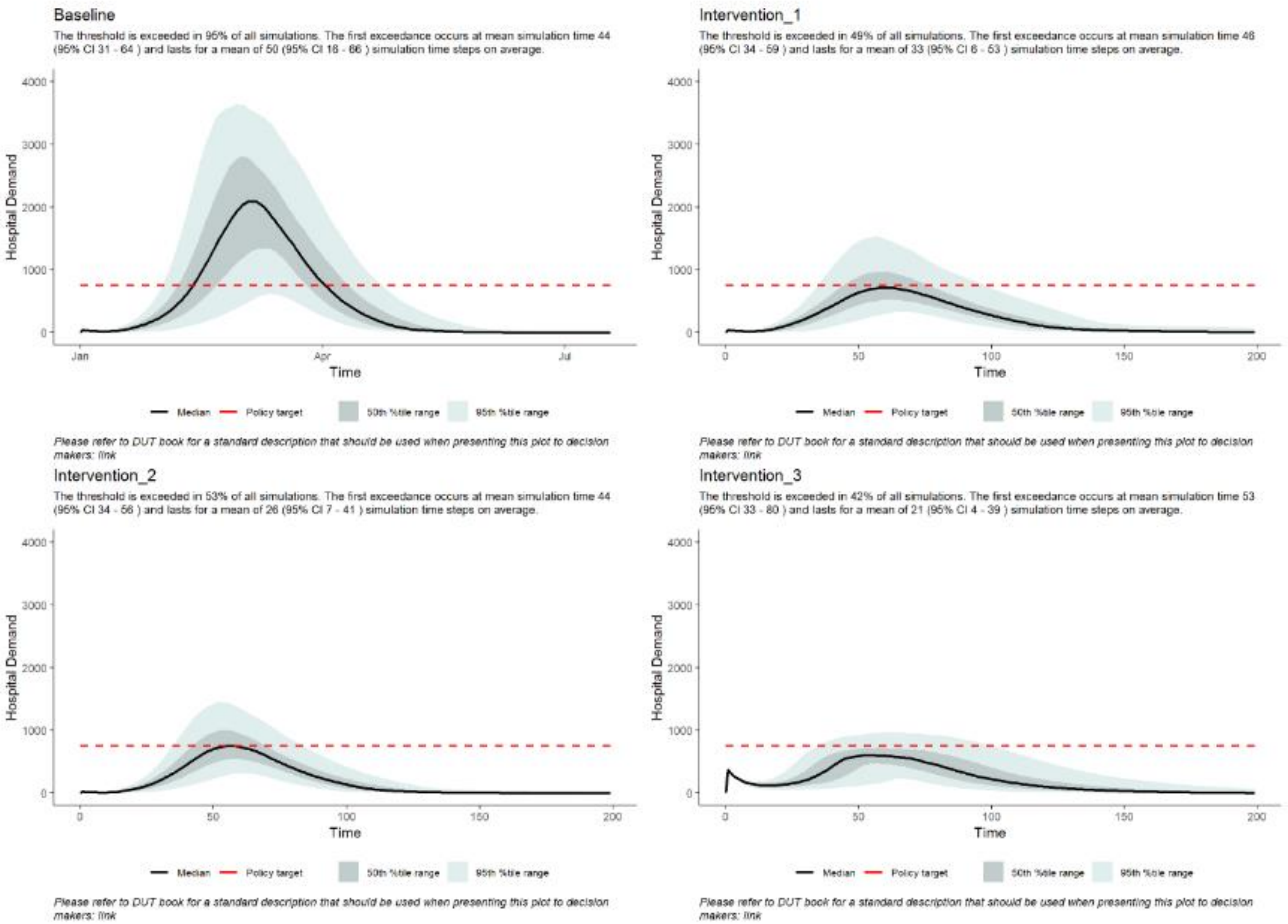

Probability Density Plot with Additional Information

- What are your general thoughts about this figure i.e., could you show this type of result to a decision maker?
  - What do you like about this figure?
  - Anything we can improve on? Or add?

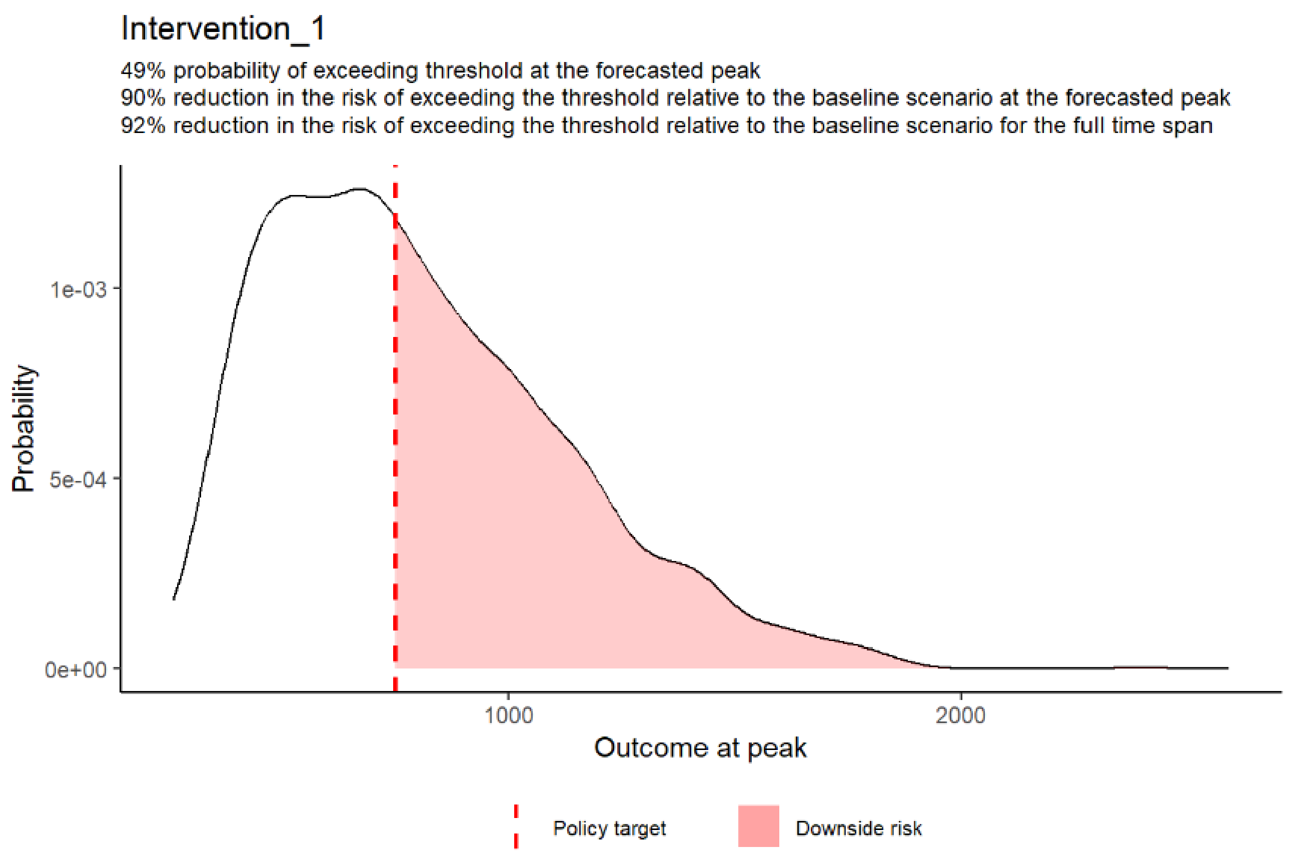

Please refer to DUT book for a standard description that should be used when presenting this plot to decision makers: link

INSTITUTE OF HEALTH ECONOMICS ALBERTA CANADA

Probability Density Plots with Additional Information

- What are your general thoughts about this figure i.e., could you show this type of result to a decision maker?
  - What do you like about this figure?
  - Anything we can improve on? Or add?

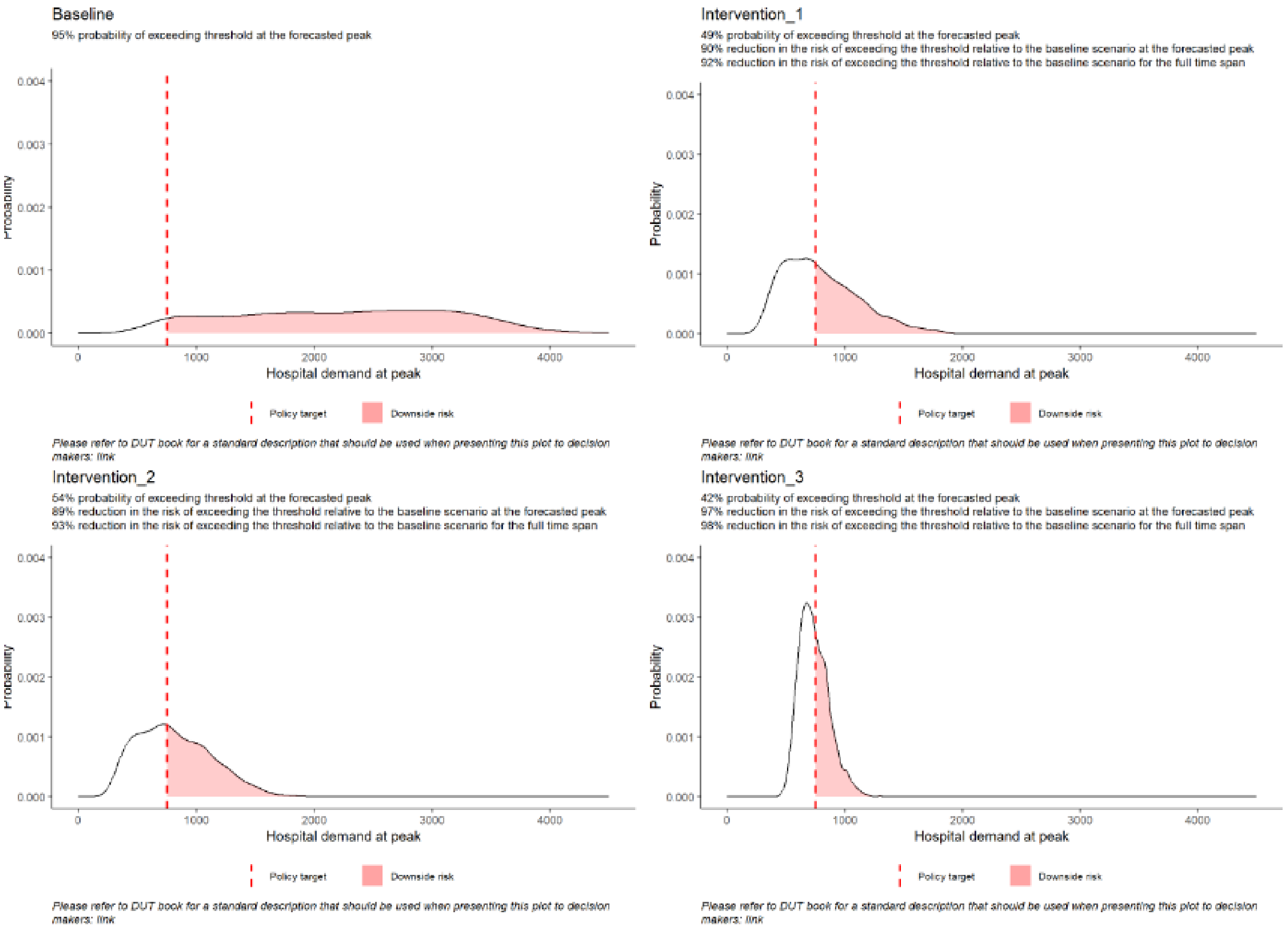

IHE INSTITUTE OF HEALTH ECONOMICS ALBERTA CANADA

Raincloud Plots

- What are your general thoughts about this figure i.e., could you show this type of result to a decision maker?
  - What do you like about this figure?
  - Anything we can improve on? Or add?

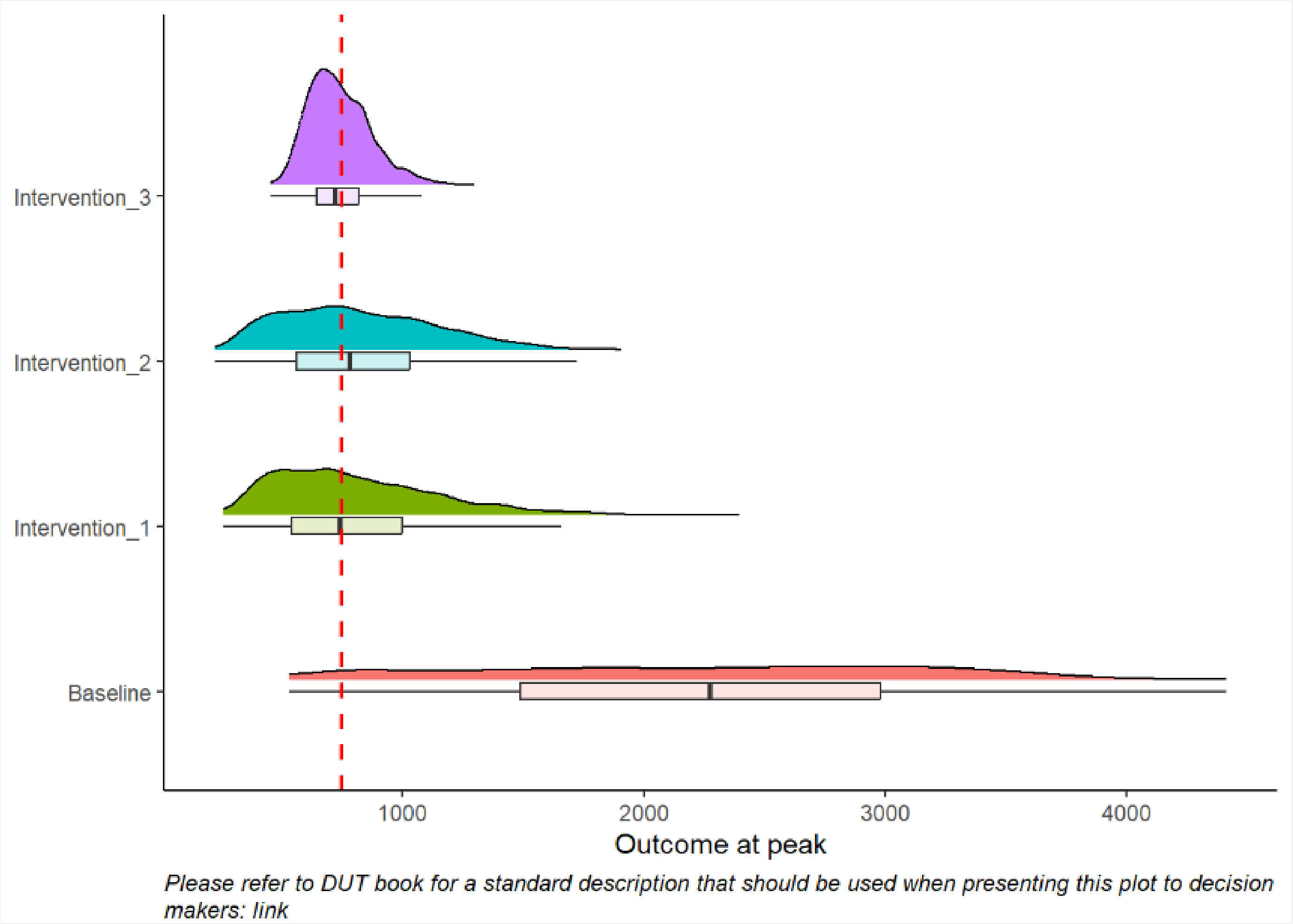

INSTITUTE OF HEALTH ECONOMICS
ALBERTA CANADA

Temporal Raincloud Plots

- What are your general thoughts about this figure i.e., could you show this type of result to a decision maker?
  - What do you like about this figure?
  - Anything we can improve on? Or add?

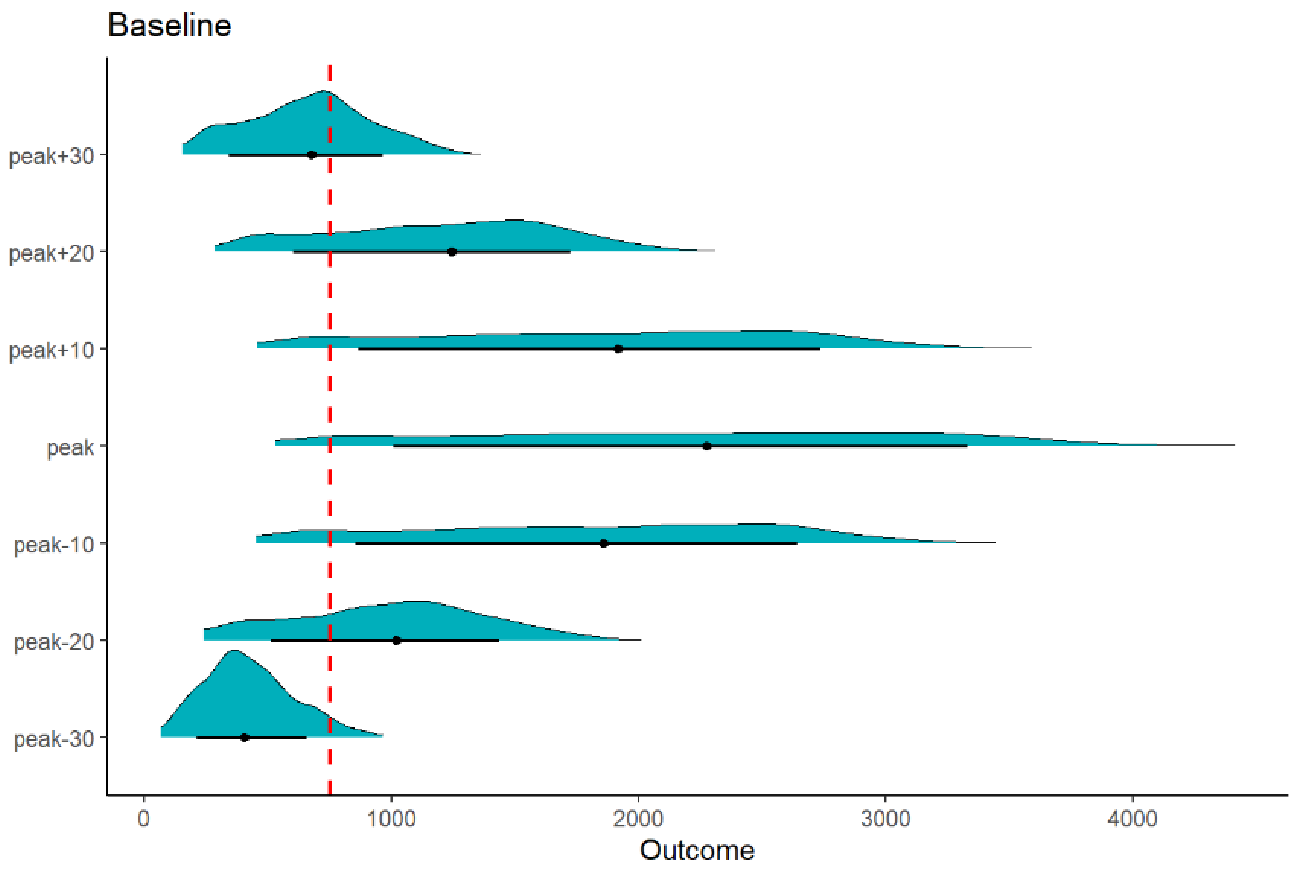

Please refer to DUT book for a standard description that should be used when presenting this plot to decision makers: link

Risk Measure

	Baseline	Intervention 1	Intervention 2	Intervention 3
Risk	45513	3,862	3,267	844
Policy risk impact	-	-91.5%	-92.8%	-98.1%

What are your thoughts about the Policy Risk Impact Value?

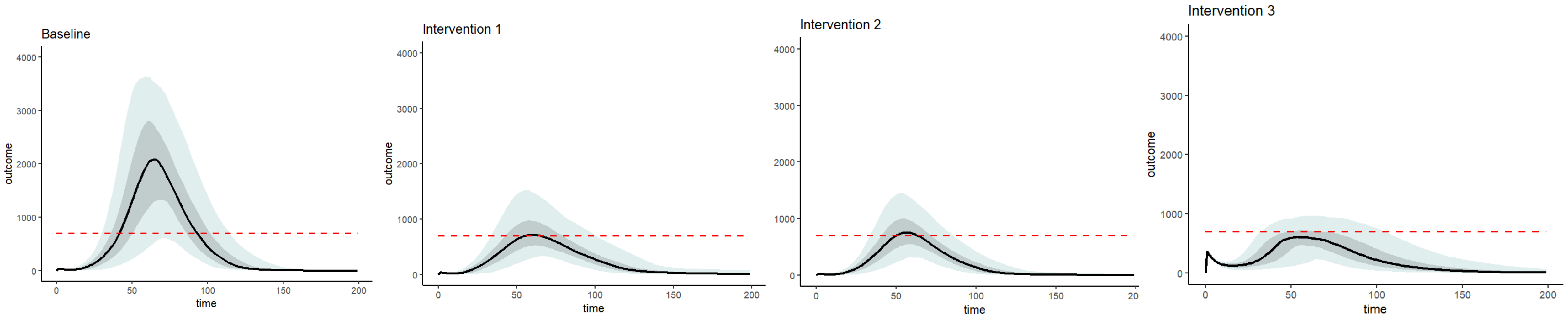

Discussion Questions

- In terms of visualizations, what types of visualizations/measures do you use to communicate uncertainty with models? Do you see something that could be useful from this toolkit and why?

- Can you see using the risk measure to describe decision uncertainty if presenting multiple policy options? Would you use this on its own or with a visualization?

- What are your thoughts about risk tolerance of decision makers and the role this might play in the risk measure?

Discussion: Standard Descriptions (15 min)

Decision Uncertainty Toolkit: Fan Plots

Standard description

These graphs visually displays the uncertainty surrounding the scenario's **probability** of *exceeding*[9] the specified policy target. They indicate not only the degree of uncertainty but also provides insight into the **magnitude** by which the intervention is likely to *exceed*[10] the target (through percentile shading) and the anticipated **duration** of the *exceedance*[11].

The **magnitude** and the **length of time** that the shaded areas *extends beyond*[12] the policy target (dashed red line) signifies the risk that the scenario will not achieve the policy objective. More shaded area *above*[13] the dashed red line for longer periods indicates a higher risk of not achieving the policy objective.

Standard description bullet points

These graph visually display:

- The uncertainty surrounding the scenario's **probability** of *exceeding*[14] the specified policy target.
- They also provides insight into the **magnitude** by which the intervention is likely to *exceed*[15] the target (through percentile shading) and the anticipated **duration** of the *exceedance*[16].
- The **magnitude** and the **length of time** that the shaded areas *extends beyond*[17] the policy target (dashed red line) signifies the risk that the scenario will not achieve the policy objective.
- More shaded area *above*[18] the dashed red line for longer periods indicates a higher risk of not achieving the policy objective.

- What are your thoughts about attempting to standardize descriptions and interpretations?
- How do you find the wording (including bullet point version)? Is this something that can be used to communicate to decision makers?

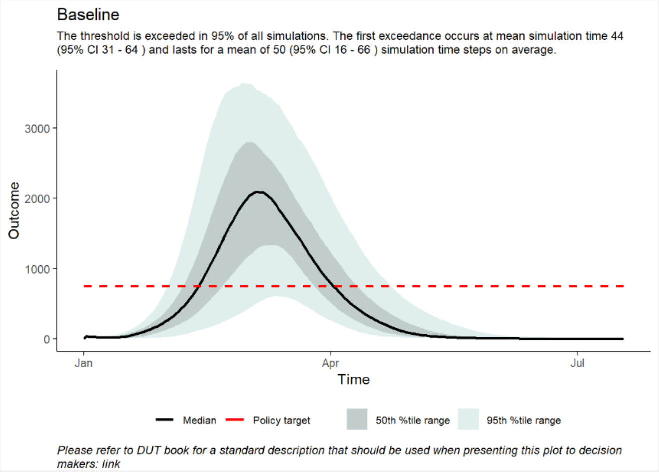

Decision Uncertainty Toolkit: Expected Risk

	Baseline	Intervention 1	Intervention 2	Intervention 3
Expected risk	45513	3,862	3,267	844
Policy risk impact	-	-91.5%	-92.8%	-98.1%

Standard description

The expected risk values in the first row of the table above capture the **probability** of the scenario *exceeding*[1] the specified policy target (i.e., how likely it is), the **magnitude** of the *exceedance*[2] from the target, and the **length of time** the *exceedance*[3] is likely to last. Higher risk values indicate a greater risk that the scenario will not achieve the policy objective.

Interpretation of the risk value is more intuitive using a relative comparator. The policy risk impact in the second row of the table compares the risk associated with each intervention to the baseline scenario. The policy risk impact is interpreted as the percent change in risk relative to the baseline scenario. For example, the expected risk of *exceeding*[4] the policy target in Intervention 1 is reduced by 91.5% relative to the baseline scenario.

Standard description bullet points

The expected risk values in the first row of the table above captures:

- The **probability** of the scenario *exceeding*[5] the specified policy target (i.e., how likely it is).
- The **magnitude** of the *exceedance*[6] from the target.
- The **length of time** the *exceedance*[7] is likely to last.
- Higher risk values indicate a greater risk that the scenario will not achieve the policy objective.

The policy risk impact in the second row of the table:

- Compares the risk associated with each intervention to the baseline scenario.
- It is interpreted as the percent change in risk relative to the baseline scenario.
- For example, the expected risk of *exceeding*[8] the policy target in Intervention 1 is reduced by 91.5% relative to the baseline scenario.

Thoughts?

Decision Uncertainty Toolkit: Risk shading

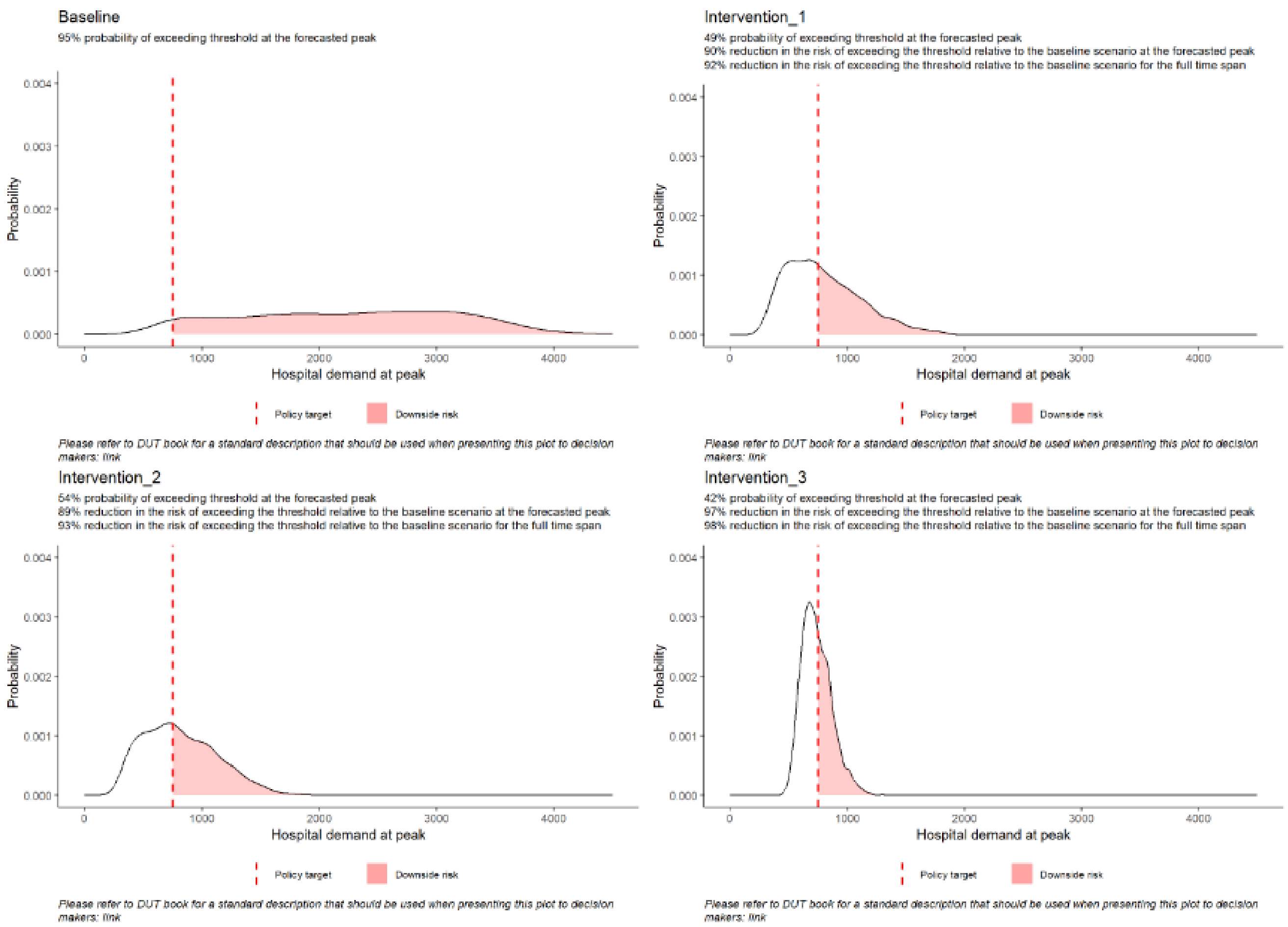

Standard description

These probability density graphs show the distribution of the *highest*[19] forecasted *outcome*[20] (i.e., the *peak*[21]). The red dashed line indicates the policy target. The shaded area indicates how likely it is that the *outcome*[22] at its forecasted *peak*[23] will *exceed*[24] the policy target, or simply, the amount of downside risk. A larger shaded area means more downside risk.

Standard description bullet points

These probability density graphs show:

- The distribution of the *highest*[25] forecasted *outcome*[26] (i.e., the *peak*[27]).
- The red dashed line indicates the policy target.
- The shaded area indicates how likely it is that the *outcome*[28] at its forecasted *peak*[29] will *exceed*[30] the policy target (i.e, the amount of downside risk).
- A larger shaded area means more downside risk.

Thoughts?

Decision Uncertainty Toolkit: Temporal plots

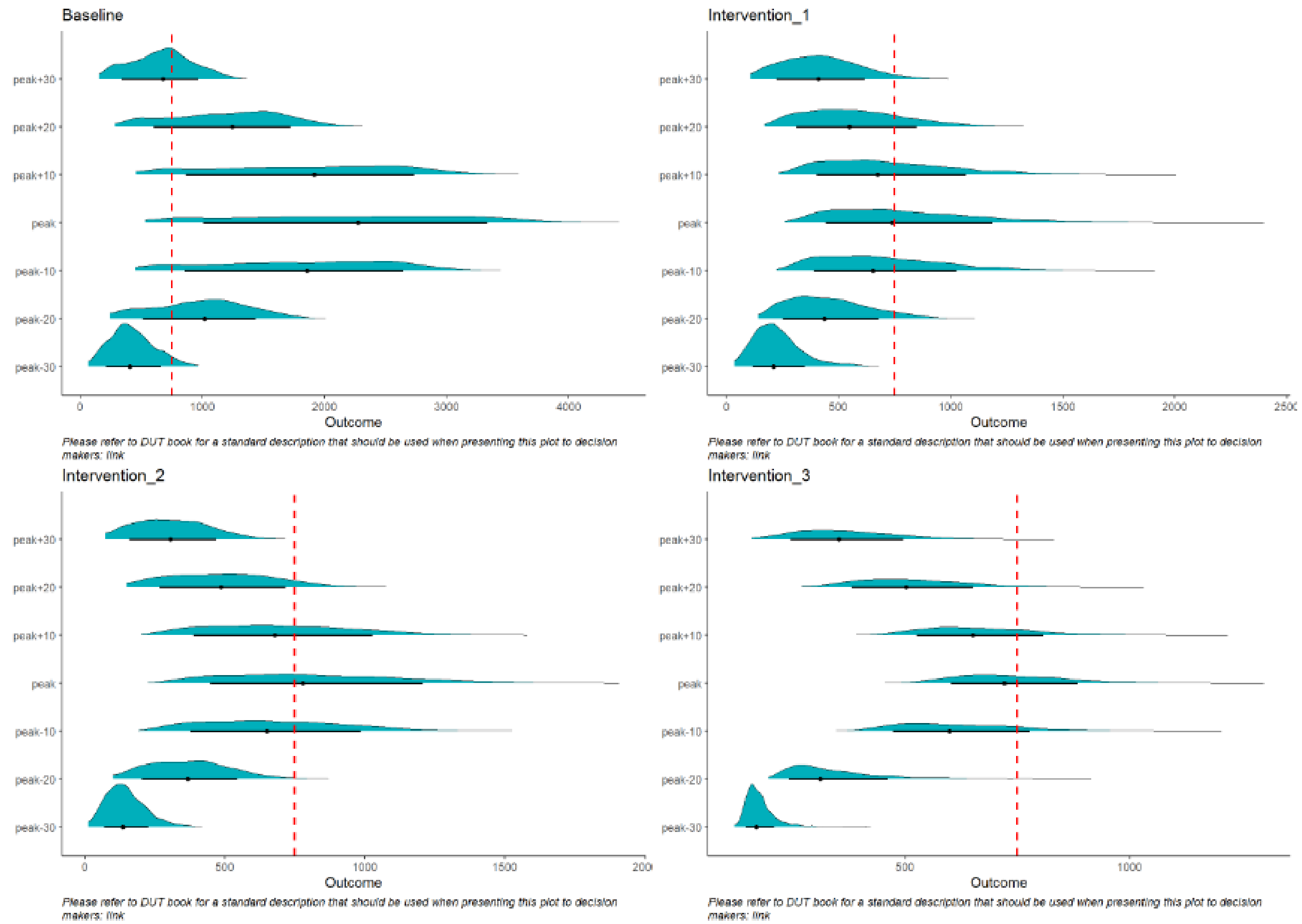

6.1.4.1 **Standard description**

These graph visually illustrates how uncertainty changes over time by showing the distributions of the forecasted *outcome*[45] over time. The distribution in the center of the plot represents the *peak*[46] *outcome*[47]. The distributions above and below show the outcome at different time points relative to the *peak*[48], both forward (above) and backward (below) in time.

The red dashed line indicates the policy target. A greater area to the *right*[49] of this line means a higher chance of the *outcome*[50] *exceeding*[51] the policy target at the specified time. Scenarios where the area to the *right*[52] of the policy target is more quickly reduced as you move away from the *peak*[53] result in a shorter time frame of higher uncertainty.

Standard description bullet points

These graphs visually illustrates how uncertainty changes over time:

- The distribution in the center of the plot represents the *peak*[54] *outcome*[55].
- The distributions above and below show the outcome at different time points relative to the *peak*[56], both forward (above) and backward (below) in time.
- The red dashed line indicates the policy target.
- A greater area to the *right*[57] of this line means a higher chance of the *outcome*[58] *exceeding*[59] the policy target at the specified time.
- Scenarios where the area to the *right*[60] of the policy target is more quickly reduced as you move away from the *peak*[61] result in a shorter time frame of higher uncertainty.

Thoughts?

Discussion: Communication of Model Uncertainty (15 min)

Discussion

- For communicating model uncertainty, what communication techniques worked and did not work in the past?
- In terms of exporting of figures and texts, would a feature to export to PowerPoint and/or Word be helpful? Are there other suggestions in terms of streamlining outputs and use?
- Any other questions?

Wrap Up (5 min)

Next Steps:

- Incorporating feedback from workshops to improve the overall toolkit
- Publishing this work and the materials in GitHub
- Exploring the feasibility to create a R package from the codes generated in this project and different outputs i.e., export feature to PowerPoint and Word that includes standard descriptions

Thank-you

mvarughese@ihe.ca
mwiggins@ihe.ca
noak@ihe.ca

SM3. Example: Presentation of Expected Risk Across Multiple Outcomes

The table below illustrates the display Expected Risk values side by side for two different outcomes (hospital demand and overall mortality) for two policy alternatives and also with multiple thresholds for hospital demand.

By examining the values in each cell of Table A1, decision–makers can directly compare the expected risk value under Policy A versus Policy B and observe how this value changes when adjusting thresholds or outcome. This granular view supports transparent trade-off analysis without presupposing that a single composite objective on the part of the decision maker. Future work could develop methods to elicit stakeholder-specific weights to aggregate these risks into a unified value to support decision making.

Table SM1. Example with multiple outcomes

Measure / Threshold	Policy A	Policy B
**Daily Hospital Demand (Threshold = 750 beds)**	0.32	0.43
**Daily Hospital Demand (Threshold = 1 000 beds)**	0.18	0.25
**Daily Mortality (Threshold = 10 deaths)**	0.12	0.08

Note: These figures are hypothetical and are not generated with the same code and data files as those reported elsewhere in the manuscript.
